# Clopidogrel versus aspirin monotherapy following dual antiplatelet therapy after percutaneous coronary intervention: an updated meta-analysis of 162,829 patients

**DOI:** 10.1007/s00228-025-03942-3

**Published:** 2026-02-06

**Authors:** Mohammed A. Elbahloul, Ahmed Mansour, Amr Galal, Walaa M. Moawad, Manar Khaled, Ahmed Wahdan Kasem, Eman E. Labeeb, Islam Y. Elgendy

**Affiliations:** 1 Faculty of Medicine, Kafr El-Shaikh University, Kafr El Sheikh, Egypt; 2 Faculty of Medicine, Al-Azhar University, Cairo, Egypt; 3 Faculty of Medicine, Fayoum University, El Fayoum, Egypt; 4 Division of Cardiovascular Medicine, Gill Heart and Vascular Institute, University of Kentucky, Lexington, KY USA

**Keywords:** Clopidogrel, Aspirin, Dual antiplatelet therapy, Percutaneous coronary intervention, Major adverse cardiovascular events, Bleeding

## Abstract

**Background:**

Dual antiplatelet therapy (DAPT) following percutaneous coronary intervention (PCI) remains a cornerstone for preventing ischemic events; however, the optimal long-term single antiplatelet therapy after completion of DAPT remains unclear. We aimed to compare clopidogrel and aspirin monotherapy after completion of the standard duration of DAPT.

**Methods:**

A systematic search was conducted on PubMed, Scopus, Cochrane Library, and Web of Science from inception to April 2025. We included randomized clinical trials and observational studies that compared aspirin versus clopidogrel monotherapy after completion of standard-duration DAPT. The primary and co-primary outcomes were major adverse cardiovascular events (MACE) and major bleeding, respectively. Using random-effects models, outcomes were expressed as risk ratios (RR) or hazard ratios (HR) with 95% confidence intervals (CI).

**Results:**

Ten studies, comprising 162,829 patients, were included. Clopidogrel was significantly associated with a lower risk of MACE (HR: 0.72, 95% CI: 0.66–0.79) and net adverse clinical events (NACE) (RR: 0.86, 95% CI: 0.73–0.99) at a weighted mean follow-up of 3.2 years. Major bleeding showed no significant difference between clopidogrel and aspirin (RR: 0.85, 95% CI: 0.60–1.21). Moreover, there was no difference between clopidogrel and aspirin in all-cause mortality, myocardial infarction, revascularization, stroke, or all bleeding.

**Conclusion:**

Among patients who underwent PCI, clopidogrel monotherapy after standard DAPT was associated with a lower incidence of MACE and NACE compared with aspirin monotherapy, without increasing the bleeding risk.

**Supplementary Information:**

The online version contains supplementary material available at 10.1007/s00228-025-03942-3.

## Introduction

The extension of antiplatelet therapy after completing standard dual antiplatelet therapy (DAPT) duration for patients post-percutaneous coronary intervention (PCI) is integral to reducing lifelong cardiovascular outcomes [1]. The 2025 AHA/ACC guidelines for management of acute coronary syndrome (ACS) recommend DAPT comprising aspirin and a P2Y12 inhibitor for 12 months [2]. Meanwhile, the 2024 ESC guidelines on chronic coronary syndromes (CCS) recommend a standard DAPT duration of 6 months after PCI [3]. However, data on the long-term antiplatelet therapy after standard DAPT is not well established. Recent guidelines recommended shorter DAPT durations followed by P2Y12 inhibitor monotherapy in ACS patients [4]. The TWILIGHT trial and comprehensive network meta-analysis support 1–3 months of DAPT in ACS patients to reduce bleeding without increasing ischemic risk [5, 6].

Long-term aspirin monotherapy after completion of standard DAPT has traditionally been the standard for secondary prevention in PCI patients, evidenced by several pivotal trials and meta-analyses [7–10]. While aspirin monotherapy has long been the standard for secondary prevention after DAPT, its residual risk of recurrent cardiovascular events remains clinically substantial. Additionally, aspirin is associated with a well-documented risk of gastrointestinal intolerance and bleeding, which can limit adherence and safety in some patients [10]. Consequently, clopidogrel has been highlighted as a promising alternative, consistent with the direction of the 2024 ESC guidelines [3, 11].

The HOST-EXAM trial [12, 13], which was the first large-scale study, demonstrated the superiority of clopidogrel over aspirin in reducing major adverse cardiovascular events (MACE) [14]. Then, these findings were further supported by the SMART-CHOICE 3 trial, which reported a significant reduction in MACE with clopidogrel, though the trial specifically targeted high-risk ischemic populations [15]. In contrast, the STOPDAPT-2 trial, which included a broader, lower-risk population, showed only a borderline, non-significant ischemic benefit [9]. Similarly, the recent STOPDAPT-3 trial reported no significant differences between clopidogrel and aspirin monotherapy in terms of cardiovascular outcomes [10].

Given the inconsistencies observed across key randomized controlled trials (RCTs) and the ongoing uncertainty regarding optimal antiplatelet monotherapy after standard DAPT, we conducted a comprehensive systematic review and meta-analysis comparing clopidogrel versus aspirin in PCI-treated patients to inform evidence-based clinical practice.

## Methods

Our systematic review was followed the guidelines of the Cochrane Handbook for Systematic Reviews of Interventions and meta-analysis, and according to the Preferred Reporting Items for Systematic Reviews and Meta-Analyses guidelines (PRISMA) [16, 17] and registered in PROSPERO ID: CRD420251129020.

### Search strategy and screening

A comprehensive literature search was performed through the PubMed, Scopus, Web of Science, and Cochrane databases, from inspection up to April 12th, 2025, using the following terms: “clopidogrel” AND “aspirin” AND “percutaneous coronary intervention” AND “monotherapy” AND “long-term prevention”. The detailed search strategy was illustrated in Supplementary Table 1. We used the EndNote software (Clarivate Analytics, Philadelphia, USA) for removing duplicates, followed by importing into the Rayyan database for screening. Four independent authors conducted blinded screening of all articles through two steps: initially by reviewing titles and abstracts, followed by full-text screening. Any conflict was resolved through discussion. We also performed hand-searching of reference lists of included studies to capture any studies not retrieved through database searches.

## Eligibility criteria

We included both RCTs and observational studies of patients who underwent PCI and completed standard-duration DAPT, irrespective of the duration, to maximize available evidence. The intervention was clopidogrel monotherapy, while the comparator was aspirin monotherapy. We excluded reviews, case series, case reports, non-English Studies, and or studies that used other P2Y12 inhibitors.

Outcomes:

The primary and co-primary outcomes were major adverse cardiovascular events (MACE) and major bleeding, as defined per the individual studies. Secondary outcomes included net adverse clinical events (NACE) as defined per the individual studies, all-cause mortality, cardiovascular mortality, myocardial infarction (MI), stent thrombosis, any revascularization, target vessel revascularization (TVR), target lesion revascularization (TLR), stroke (including ischemic and hemorrhagic subtypes), any bleeding, major bleeding, gastrointestinal bleeding, and intracranial bleeding. The definitions of outcomes and inclusion and exclusion criteria per study were illustrated in Supplementary Tables 2 and 5.

## Data extraction

Data were extracted to a specified data extraction sheet by four independent authors. The extracted data included: study design, country, total sample size, DAPT regimen after PCI, duration of DAPT regimen, follow-up duration, baseline characteristics of the included population (age, sex, BMI, diabetes, hypertension, dyslipidemia, current smoking, heart failure, chronic kidney disease (CKD), stroke, previous coronary artery bypass grafting, previous PCI, and outcomes of interest. Any disagreement among authors was resolved through discussion.

## Quality assessment

We assessed the RCTs using the updated Cochrane Risk of Bias 2 (ROB 2) tool [18]. Studies were categorized into low risk of bias, high risk of bias, or some concerns. while the observational studies were assessed using the Newcastle–Ottawa Scale (NOS) [19]. Every question in each domain gets one or zero stars, except comparability gets two or zero stars. The authors’ decisions were classified as: good quality (≥ 7 stars), fair quality (5–6 stars), or low quality (≤ 4 stars). Any conflict was resolved through discussion.

### Statistical analysis

All analyses were conducted using R Statistical Software (version 4.4.3). For studies that reported only event counts without time-to-event data, we calculated risk ratios (RRs) with 95% confidence intervals for dichotomous outcomes. For studies providing Kaplan–Meier curves, we extracted hazard ratios (HRs) to capture time-to-event information. Since RR estimates can be affected by differences in follow-up duration across studies, we additionally performed an HR-based analysis for the primary outcome (MACCE) to offer a more robust time-to-event perspective. We used random-effects models to account for the anticipated heterogeneity in study design, follow-up duration, and outcomes definition. Prespecified subgroup analyses were performed based on study design, clinical presentation, high bleeding risk, diabetes mellitus (DM), complex PCI, CKD, proton pump inhibitor (PPI) use, multivessel disease, P2Y12 inhibitor used before randomization, drug-eluting stent (DES) generation, and DAPT duration. We adjusted the p-values for subgroup interactions using the Benjamini–Hochberg false discovery rate (BH-FDR) method to minimize false-positive findings [20].

We assessed statistical heterogeneity among studies using I² statistic. An I² value of 50% or higher indicated high heterogeneity, 25% to 50% indicated moderate heterogeneity, and 25% or below indicated low heterogeneity [21]. We performed leave-one-out sensitivity analyses by sequentially excluding individual studies to assess the robustness of the evidence and ensure the overall results were not altered. Publication bias was assessed visually using funnel plots and statistically using Egger’s test [22]. Univariate meta-regression was performed to explore the effect of baseline characteristics, including: mean age, mean BMI, mean left ventricular ejection fraction (LVEF), male, hypertension, dyslipidemia, history of stroke, current smokers, and previous PCI. An E-value analysis was performed to assess whether unmeasured confounders could explain the observed significant association [23].

We used Kaplan-Meier curves (KM) to report the cumulative incidence of MACE rates following the curve approach and Guyot method [24, 25]. KM curves were extracted from published KM using WebPlotDigitizer software to obtain time-to-event coordinates, and then the proposed algorithm was applied to reconstruct approximate individual patient data (IPD) through “IPDfromKM” package [21]. The reconstructed IPDs were merged into a single dataset using ggplot2”, “survival”, “survminer”, and “coxph” packages, KM curve, the Cox proportional hazard ratio, and the time-dependent hazard ratio were obtained.

## Results

### Search results and study selection

**Fig. 1:**
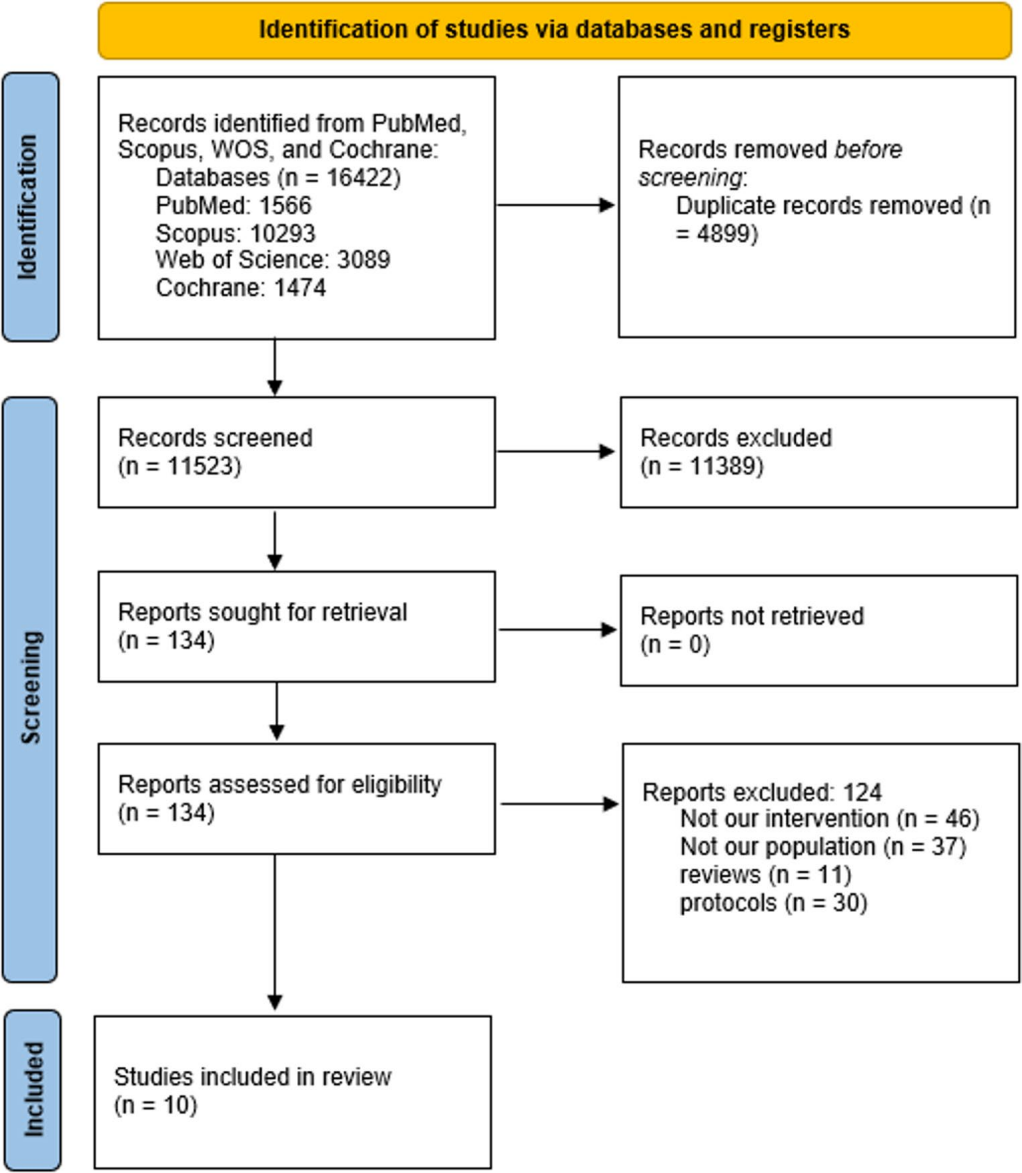
PRISMA flow diagram

The literature search initially identified 16,422 records through database searching. After removing 4,899 duplicates, 11,389 records were excluded based on title and abstract screening. A further 124 records were excluded following full-text review. Finally, 10 studies fulfilled the eligibility criteria and were included in the meta-analysis (Fig. 1).

## Characteristics of included studies

A total of 10 studies [9, 10, 12, 13, 15, 26–31] including 162,829 participants (80,613 in the clopidogrel monotherapy group and 81,434 in the aspirin monotherapy group). Of which, 4 were RCTs and 6 were observational studies. One study was available only as an abstract [26]. All studies were conducted in East Asia: 6 studies in South Korea, 2 in Japan, and 2 in China. The mean age was 70.7 years in the clopidogrel group and 69.1 years in the aspirin group. The weighted mean follow-up duration was 3.2 years. Standard DAPT duration ranged from 1 to 18 months. The characteristics of the included studies were summarized in Table 1. Baseline patient characteristics for both groups are detailed in Supplementary Table 3.

**Table 1:** Summary of the included studies

Study ID	Study design	Country	Total sample size	DAPT Regimen After PCI	Duration of DAPT regimen	Follow-up duration	Clopidogrel monotherapy dose	Aspirin monotherapy dose	PCI indications
Choi et al. 2025 (SMART-CHOICE 3)	Multicentre, prospective, open-label RCT	South Korea	5506	Aspirin + clopidogrel (61.8% in clopidogrel group and 62.8% in aspirin group), Aspirin + prasugrel (11% in clopidogrel group and 12.4% in aspirin group), Aspirin + ticagrelor (24.8% in clopidogrel group and 27.1% in aspirin group).	at least 12 months for myocardial infarction patients and at least 6 months for other PCI indications before switching to monotherapy	2.3 years	75 mg/day	100 mg/day	coronary artery disease (either AMI or other symptomatic CAD
Watanabe et al. 2024 (STOPDAPT-3)	Multicentre, open-label, adjudicator-blinded, RCT	Japan	5962	Aspirin and prasugrel (Aspirin group), Prasugrel monotherapy for 1 month, followed by clopidogrel monotherapy (Clopidogrel group).	1 month	1 year	75 mg/day	81–100 mg/day	AMI
Watanabe et al. 2024 (STOPDAPT-2)	Multicentre, open-label, adjudicator-blinded, RCT	Japan	3005	Patients received either clopidogrel 75 mg or prasugrel 3.75 mg	1 month for clopidogrel group, and 12 months for aspirin group.	5 years	75 mg/day	81–200 mg/day	AMI
Chung-Ang et al. 2024	population-based cohort	Korea	133,343	N.A	At least 6 m	Median 3.3 years (IQR: 1.3–6.2 years)	N.A	N.A	coronary artery disease
Lan et al. 2024	Retrospective, single-center Cohort study	China	1055	Aspirin 100 mg and clopidogrel 75 mg per day.	12 months	Mean 25 months (SD: 8.4 months)	75 mg/day	100 mg/day	coronary artery disease
Koo et al. 2021, Kang et al. 2024 (HOST-EXAM)	prospective multicentre, RCT	Korea	5438	mainly aspirin plus clopidogrel	6–18 months	5.8 years	(75 mg once daily)	(100 mg once daily)	AMI
Sim et al. 2019	a prospective, open, online multi-center data cohort	Korea	1819	Aspirin and a P2Y12 inhibitor	at least 12 months	2 Years	(75 mg once daily)	(100 mg once daily)	AMI
Park et al. 2016	Retrospective Cohort Study	Korea	3243	DAPT with aspirin and a P2Y12 receptor inhibitor	12-month	3 years	(75 mg once daily)	(100 mg once daily)	stable coronary artery disease or acute coronary syndrome
Zhuang et al. 2014	Retrospective Cohort Study	China	755	DAPT with aspirin (100 mg/d) and clopidogrel (75 mg/d)	12 months	3 years	(25, 75 mg once daily)	(100 mg once daily)	coronary artery disease
Jang et al. 2025	Retrospective Cohort Study	Korea	2703	DAPT with aspirin (100 mg/d) and clopidogrel (75 mg/d)	12 months	1 Years	(75 mg once daily)	(100 mg once daily)	coronary artery disease

*AMI* Acute Myocardial Infarction, *DAPT* Dual Antiplatelet Therapy, *IQR* Interquartile Range, *PCI* Percutaneous Coronary Intervention, *RCT* Randomized Clinical Trial, *SD* Standard Deviation

### Risk of bias assessment

Overall, all RCTs were judged to have some concerns driven by their open-label design, which may have introduced performance bias (Supplementary Fig. 1). For observational studies, 5 studies were scored between 7 and 9 stars and were rated as good quality, and one study scored 6 stars and was rated as fair quality (Supplementary Table 4).

### Primary outcome

**Fig. 2:**
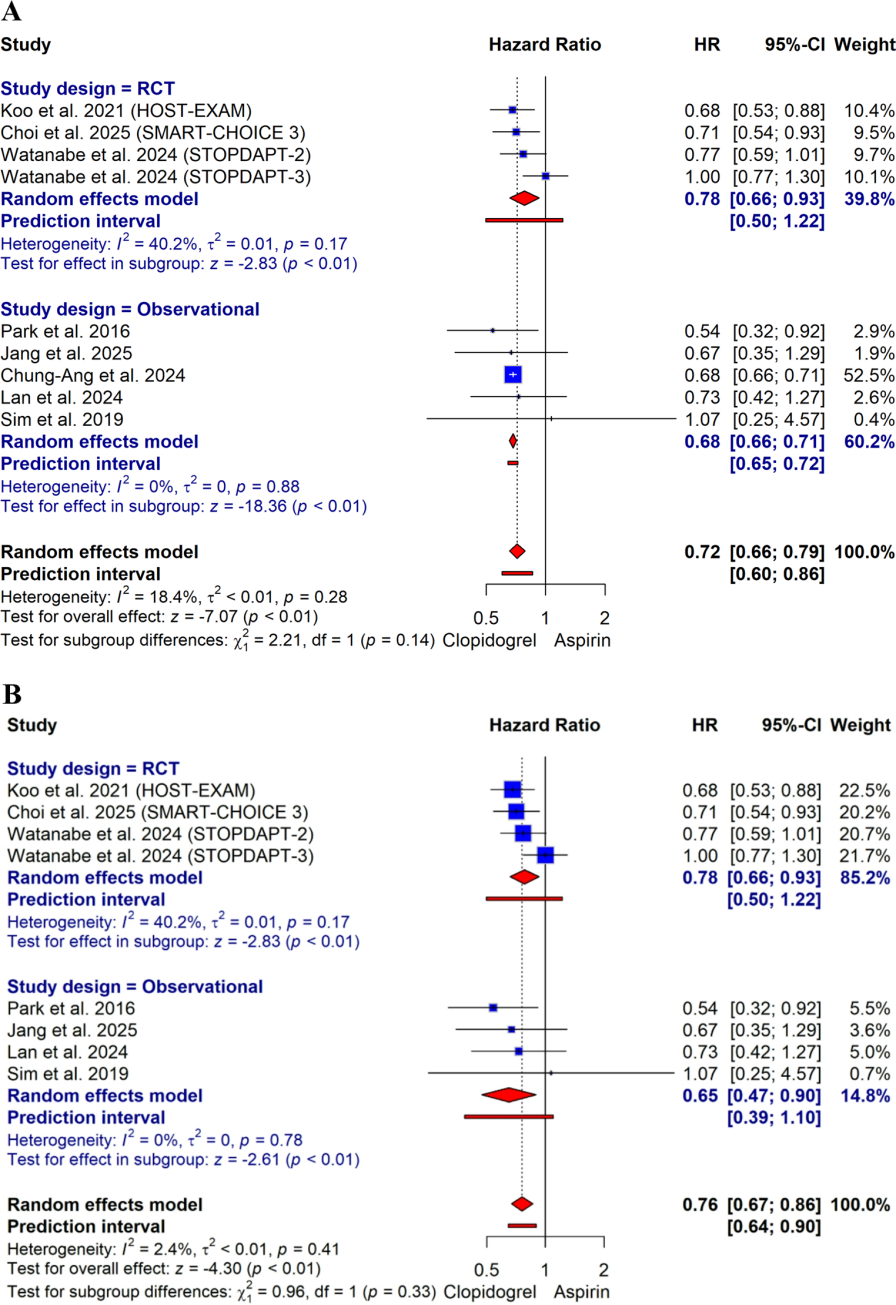
**A** Forest plot of major adverse cardiovascular events (MACE) based on hazard ratio (HR) estimates. **B** Sensitivity analysis for MACE excluding Chung-Ang et al

The primary outcome was reported in all included studies. The definition of MACE used in each study is illustrated in Supplementary Table 2**.** The incidence of MACE was 3.1% in the clopidogrel monotherapy group and 4.4% in the aspirin monotherapy group. Clopidogrel monotherapy was associated with a significant reduction in the risk of MACE compared to aspirin monotherapy (HR: 0.72, 95% CI: 0.66–0.79; *p* < 0.01; with low heterogeneity I² = 18.4%, *p* = 0.28; Fig. 2A). The prediction interval for this outcome was (0.60–0.86), and the number needed to treat (NNT) was 77. The E-value for the point estimate was 2.12, and the E-value for the confidence interval ranged from 1.85 to 2.40.

**Fig. 3:**
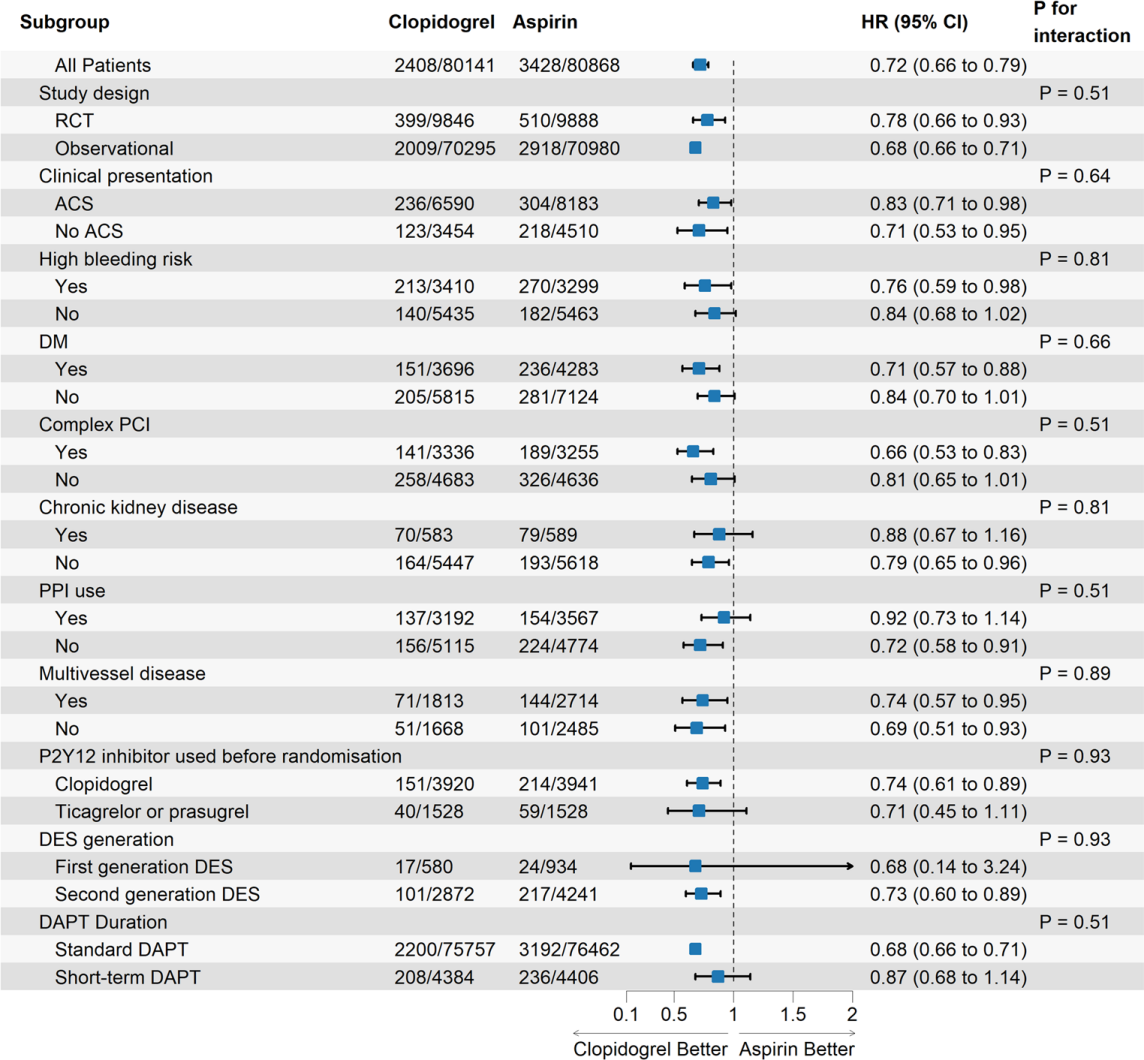
subgroup analysis plot for major adverse cardiovascular events (MACE)

A sensitivity analysis excluding the abstract-only study by Chung-Ang et al. showed a consistent result (HR: 0.76, 95% CI: 0.67–0.86; with low heterogeneity I² = 2.4%, *p* = 0.41; Fig. 2B). A secondary analysis based on risk ratio estimates also demonstrated a significant reduction in MACE (RR: 0.79, 95% CI: 0.70–0.90; with high heterogeneity I² = 56.3%, *p* = 0.01; Supplementary Fig. 2). Subgroup analyses were conducted to assess the consistency of the treatment effect (Fig. 3). Subgroup analyses showed no significant interaction was detected, indicating that the treatment effect of clopidogrel is consistent across populations based on study design, clinical presentation, high bleeding risk, diabetes mellitus, complex PCI, chronic kidney disease, PPI use, multivessel disease, P2Y12 inhibitor use prior to randomization, and DES generation.

**Fig. 4:**
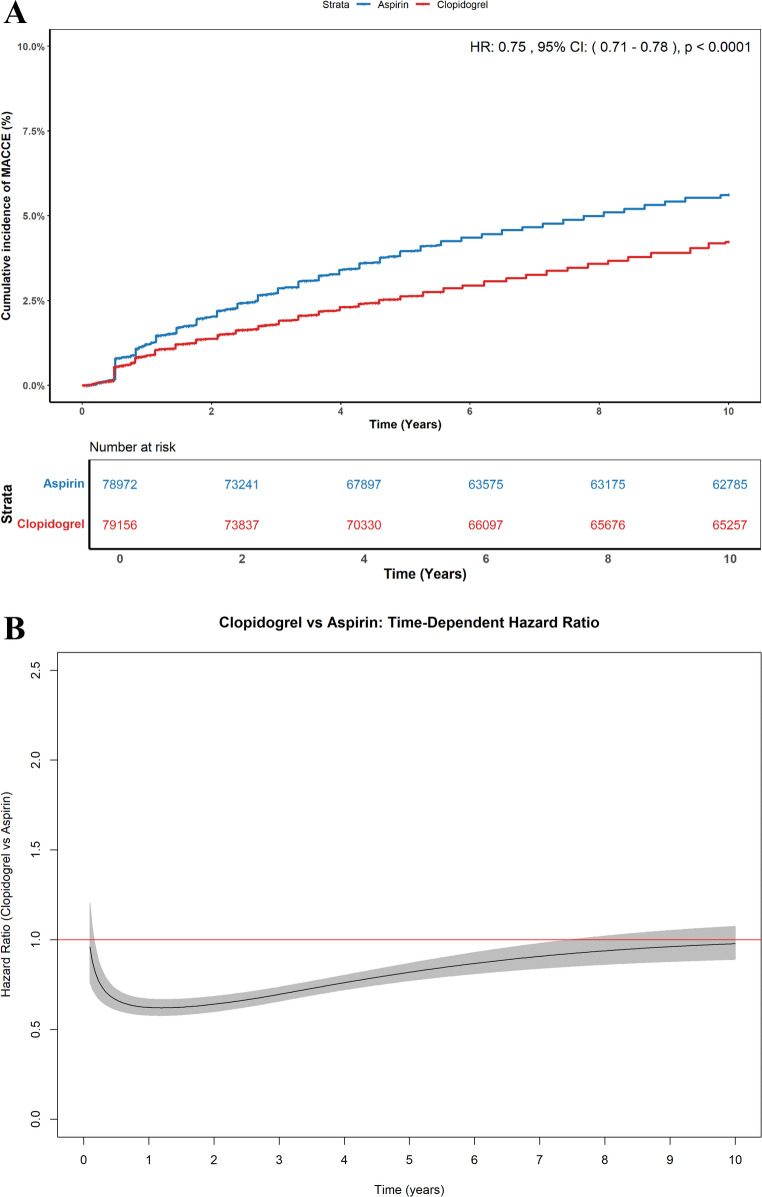
**A** Pooled Kaplan-Meier curve showing cumulative incidence of MACE between Clopidogrel and Aspirin groups. **B **Hazard ratio for major adverse cardiovascular events (MACE) over time

Seven studies were included in Kaplan-Meier curves for MACE. Clopidogrel monotherapy was associated with a lower risk of MACE compared to aspirin monotherapy over a 10-year follow-up period (HR = 0.75, 95% CI: 0.71–0.78; *p* < 0.0001) **(**Fig. 4A**)**. The cumulative incidence was 4.3% in the clopidogrel group and 5.6% in the aspirin group. Visual assessment of the Schoenfeld residuals plot and the log-log survival curves suggested a violation of the proportional hazard assumption over time (Supplementary Figs. 3–4). A time-dependent Hazard Ratio analysis demonstrated that the HR for clopidogrel versus aspirin was variable over time; the benefit of clopidogrel appeared more pronounced in earlier years and tended to attenuate in later years, although the HR generally remained below 1.0 **(**Fig. 4B**)**.

### Co-primary outcome

**Fig. 5:**
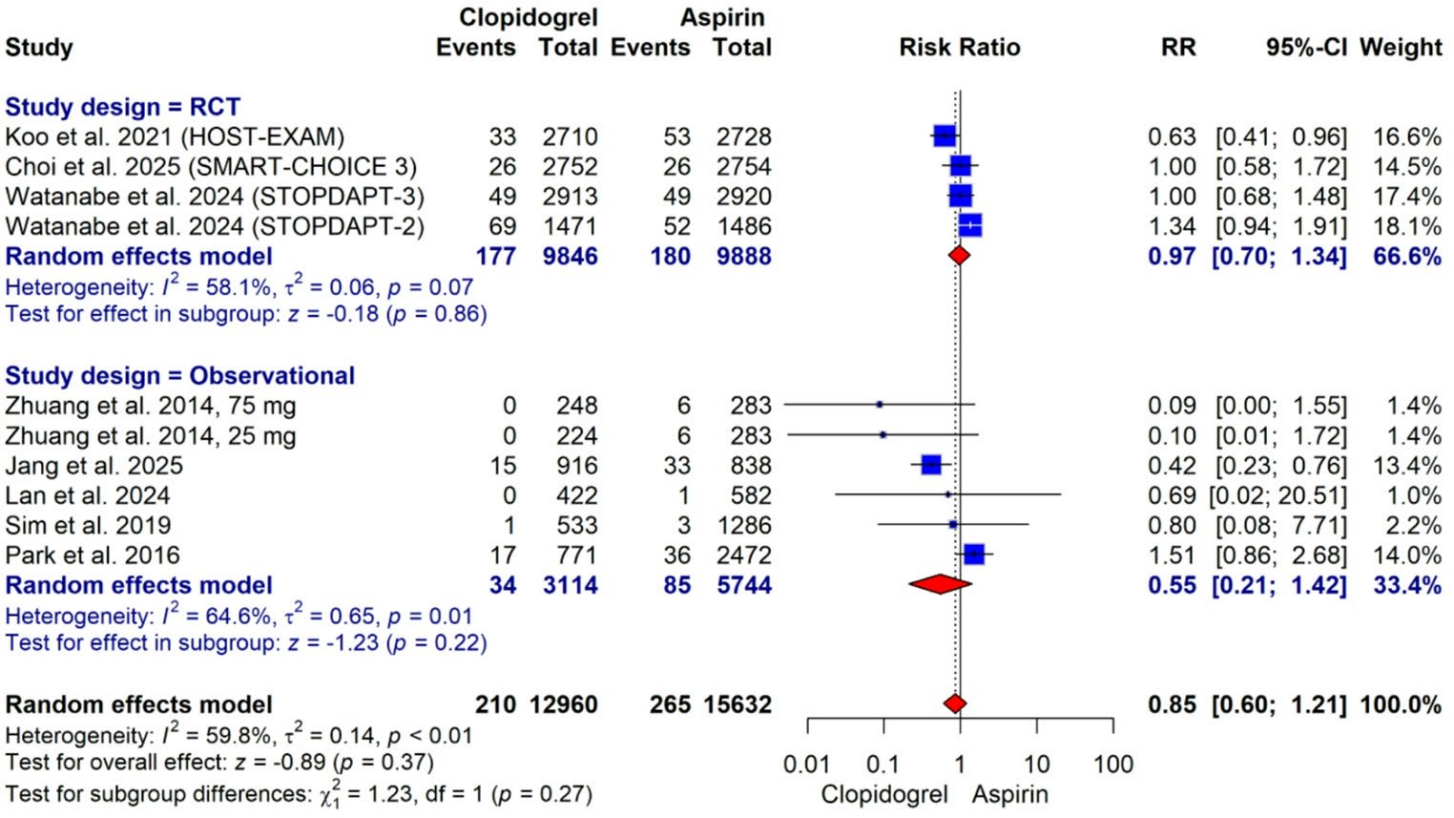
Forest plot for major bleeding

The incidence of major bleeding was 1.6% in the clopidogrel monotherapy group and 1.7% in the aspirin monotherapy group. No statistically significant difference was observed between the groups in major bleeding (RR: 0.85, 95% CI: 0.60–1.21; *p* = 0.37; with high heterogeneity I² = 59.8%, *p* < 0.01; Fig. 5).

### Secondary outcomes

A significant reduction favoring clopidogrel monotherapy was observed for NACE (RR: 0.86, 95% CI: 0.73–1.00.73.00; *p* = 0.04; I² = 49.3%, *p* = 0.07) (Supplementary Fig. 5). No statistically significant differences were observed between groups for other secondary outcomes, including all-cause mortality (RR: 0.90, 95% CI: 0.71–1.14; *p* = 0.39; I² = 63.7%, *p* < 0.01), and cardiovascular mortality (RR: 0.91, 95% CI: 0.75–1.12; *p* = 0.38; I² = 9.3%, *p* = 0.36), MI (1.7 vs. 1.9, RR: 0.84, 95% CI: 0.69–1.03; *p* = 0.10; I² = 22.1%, *p* = 0.24), stent thrombosis (RR: 0.64, 95% CI: 0.38–1.07; *p* = 0.09; I² = 0.0%, *p* = 0.74), any revascularization (RR: 0.97, 95% CI: 0.85–1.10; *p* = 0.62; I² = 0.0%, *p* = 0.57), TLR (RR: 0.93, 95% CI: 0.75–1.15; *p* = 0.50; I² = 0.0%, *p* = 0.40), TVR (RR: 0.80, 95% CI: 0.61–1.05; *p* = 0.11; I² = 0.0%, *p* = 0.99), stroke (RR: 0.78, 95% CI: 0.60–1.01; *p* = 0.06; I² = 26.2%, *p* = 0.23), ischemic stroke (RR: 0.78, 95% CI: 0.58–1.04; *p* = 0.09; I² = 0.0%, *p* = 0.62), hemorrhagic stroke (RR: 0.72, 95% CI: 0.29–1.79; *p* = 0.48; I² = 53.9%, *p* = 0.09), all bleeding (RR: 0.77, 95% CI: 0.59–1.02; *p* = 0.07; I² = 83.3%, *p* < 0.01), gastrointestinal bleeding (RR: 0.99, 95% CI: 0.76–1.30; *p* = 0.96; I² = 33.8%, *p* = 0.18), intracranial bleeding (RR: 1.03, 95% CI: 0.60–1.77; *p* = 0.90; I² = 45.6%, *p* = 0.14) (Table 2, Supplementary Figs. 6–18).

**Table 2:** Outcome summary

Outcome	Number of included studies	Incidence % (Clopidogrel)	Incidence% (Aspirin)	RR [95% CI]	*P*-value (Overall Effect)	I² (Overall)	RFI^¶^
MACE	10	3.1%	4.4%	0.79 [0.70; 0.90]	< 0.01	56.3%	N.A^*^
NACE	7	5.6%	6.2%	0.86 [0.73; 1.00]	0.04	49.3%	N.A
All-cause mortality	8	3.3%	3.6%	0.90 [0.71; 1.14]	0.39	63.7%	32
Cardiovascular mortality	8	1.8%	1.9%	0.91 [0.75; 1.12]	0.38	9.3%	31
MI	9	1.7%	1.9%	0.84 [0.69; 1.03]	0.10	22.1%	7
Stent thrombosis	6	0.2%	0.34%	0.64 [0.38; 1.07]	0.09	0.0%	2
Any revascularization	6	4.0%	3.9%	0.97 [0.85; 1.10]	0.62	0.0%	47
TLR	4	1.7%	1.8%	0.93 [0.75; 1.15]	0.50	0.0%	25
TVR	4	1.4%	1.6%	0.80 [0.61–1.05]	0.11	0.0%	16
Stroke	7	1.2%	1.5%	0.78 [0.60; 1.01]	0.06	26.2%	1
Ischemic stroke	5	0.8%	0.9%	0.78 [0.58; 1.04]	0.09	0.0%	12
Haemorrhagic stroke	4	0.2%	0.3%	0.72 [0.29; 1.79]	0.48	53.9%	4
All bleeding	7	2.2%	2.6%	0.77 [0.59; 1.02]	0.07	85.3%	1
Major bleeding	9	1.6%	1.7%	0.85 [0.60; 1.21]	0.37	59.8%	37
Gastrointestinal bleeding	6	1.6%	1.5%	0.99 [0.76; 1.30]	0.96	33.8%	28
Intracranial bleeding	4	0.7%	0.6%	1.03 [0.60; 1.77]	0.90	45.6%	17

*CI* Confidence Interval, *MACE *Major Adverse Cardiovascular Events, *MI*: Myocardial Infarction, *N.A *Not Applicable, *NACE *Net Adverse Clinical Events, *RR *Risk Ratio, *RFI *Reverse Fragility Index, *TLR *Target lesion revascularization, *TVR *Target Vessel Revascularization

The Reverse Fragility Index (RFI) quantifies the robustness of non−significant results by calculating the minimum number of event−status changes required to convert a non−significant finding into a statistically significant one

* Not Applicable because those outcomes were statistically significant, and RFI is only applied to non−significant results

### Publication bias and sensitivity analysis

The publication bias of MACE was assessed. Visual inspection of the funnel plot (Supplementary Fig. 19) suggested symmetry and Egger’s test yielded a p-value of 0.32, indicating no potential publication bias. Trim and fill analysis identified 2 potentially missing studies (Supplementary Fig. 20). Adjusted effect size remained statistically significant (HR: 0.69; 95% CI: 0.61–0.78), supporting the robustness of the overall findings.

Regarding major bleeding, visual inspection of the funnel plot showed asymmetry, indicating potential publication bias (Supplementary Fig. 21). Although Egger’s test was non-significant (*p* = 0.19), trim-and-fill analysis imputed 5 potentially missing studies. The adjusted effect size remained non-significant (RR: 1.18; 95% CI: 0.79–1.76) (Supplementary Fig. 22).

Moreover, leave-one-out sensitivity analysis showed that the outcomes of MACE, major bleeding, all-cause mortality, cardiovascular mortality, stent thrombosis, any revascularization, TVR, TLR, haemorrhagic stroke, gastrointestinal bleeding, and intracranial bleeding were robust and not influenced by any individual study (Supplementary Figs. 23–33). However, MI, stroke, Ischaemic stroke, and all bleeding were sensitive to specific studies; omitting these studies revealed a significant difference favoring clopidogrel over aspirin (Supplementary Figs. 34–37). Additionally, the effect estimate of NACE was unstable and was highly influenced by individual studies (Supplementary Fig. 38).

### Meta-regression

Meta-regression analysis showed a significant association between MACE and mean age (coefficient: 0.04, *p* < 0.01), Hypertension (coefficient: 0.02, *p* = 0.04), and LVEF (coefficient: −0.07, *p* = 0.04) (Supplementary Table 4, Supplementary Fig. 39–41).

## Discussion

In this comprehensive meta-analysis of 10 studies involving 162,829 patients, we assessed the efficacy of clopidogrel monotherapy compared to aspirin monotherapy following DAPT in patients undergoing PCI. We found that clopidogrel was associated with a 21% relative risk reduction in MACE over a mean follow-up of 3.2 years without an increased risk of major bleeding. These findings were consistent across several subgroup analyses, including RCTs vs. observational studies. However, there were no significant differences between the two groups in terms of secondary outcomes, including all-cause mortality, cardiovascular mortality, stent thrombosis, MI, and stroke (including ischemic and hemorrhagic stroke).

Although DAPT is commonly administered for the first year after PCI, single antiplatelet therapy usually represents the principal strategy for long-term secondary prevention. In the 2025 ACC/AHA/ACEP/NAEMSP/SCAI and 2023 ESC guidelines for the management of patients with ACS, the default antiplatelet regimen following PCI involves administering DAPT with aspirin and a P2Y₁₂ inhibitor for a minimum of 12 months [2, 32].

Our findings should be interpreted within the ongoing clinical debate between extended DAPT and transition to long-term single antiplatelet therapy, which requires balancing ischemic and bleeding risks. Recent trials and updated guidelines support shorter DAPT durations (1–3 months) followed by P2Y12 inhibitor monotherapy, as demonstrated by TWILIGHT and confirmed in recent meta-analyses [4–6]. This aspirin-free strategy is now recommended for ACS patients [4]. While these studies support transition to monotherapy, our analysis addresses the next clinical question, therapy selection, and suggests that clopidogrel is a more effective option than aspirin for long-term secondary prevention of MACE in the patient populations included in this study.

Recent evidence, including Piccolo et al., showed that a personalized, risk score-guided duration of DAPT after PCI (ranging from 3 to 24 months) significantly lowers NACE, with benefit observed in high ischemic risk patients compared to standard 12-month DAPT, mainly due to fewer MI and revascularizations, without increased bleeding events [33]. Meanwhile, switching to monotherapy after an initial DAPT duration can decrease bleeding without significantly increasing MACE in lower-risk groups [34]. Consistent with our findings, the SMART-CHOICE 3 trial [15] showed that clopidogrel monotherapy was superior to aspirin in reducing MACE among high-risk patients following PCI. The HOST-EXAM trial [12, 13] aligned with these results in a broader cohort without limitations based on lesion complexity or clinical risk. However, SMART-CHOICE 3 reported higher cardiovascular mortality and non-fatal MI rates. Conversely, the STOPDAPT-3 trial reported no significant differences in MACE outcomes between clopidogrel and aspirin [10]. A key reason for this contrasting finding was the difference in antiplatelet strategies preceding monotherapy. In HOST-EXAM and SMART-CHOICE 3, patients underwent standard DAPT for about 12 months before transitioning to monotherapy. Conversely, STOPDAPT 3 trial employed a short-term, intensified approach with potent P2Y₁₂ inhibition, aspirin plus prasugrel for one month in the aspirin arm, and prasugrel monotherapy in the clopidogrel arm. The initial combination regimen in the aspirin group likely conferred a sustained reduction in risk, thereby attenuating observable differences between the groups during the maintenance phase [9, 10]. Consequently, our pooled analysis, appropriately weighted to reflect the larger sample sizes of long-DAPT trials and observational studies, reinforces that the clinical advantage of clopidogrel is most pronounced and generalizable in the typical setting of patients who have completed a standard 6 to 12-month course of DAPT.

The observed advantage of clopidogrel over aspirin in this analysis can be attributed to several factors and mechanistically supported explanations. Firstly, the P2Y₁₂ receptor serves as a key regulator of hemostasis, amplifying platelet activation in response to various agonists such as thromboxane A₂ [35, 36]. Furthermore, among patients with coronary stents who completed at least 6 months of DAPT, a randomized crossover study demonstrated that clopidogrel monotherapy resulted in more pronounced platelet suppression and attenuated coagulation markers relative to aspirin monotherapy [37]. Clopidogrel has additionally demonstrated anti-inflammatory properties, notably by suppressing leukocyte activity and diminishing platelet–leukocyte interactions [38]. Such anti-inflammatory properties may play a role in the observed reduction of MACE associated with clopidogrel monotherapy. Secondly, the current analysis found no statistically significant difference between the groups in terms of major or clinically relevant bleeding risk. This analysis yields findings that are concordant with previous observational data, interventional studies, and aggregated meta-analyses [10, 15, 29, 39]. This observed similarity in gastrointestinal bleeding rates may be explained by both a reduced number of high-bleeding-risk patients and a relatively high prevalence of PPI use across the cohort. This result was consistent with that observed in the CAPRIE trial [40]. Taken together, these findings suggested that clopidogrel monotherapy is a promising option in the stabilized period following PCI.

An important clinical consideration with clopidogrel monotherapy is the impact of CYP2C19 gene polymorphisms, which can affect clopidogrel metabolism and potentially increase the risk of thrombotic events in poor metabolizers. However, our findings provide reassurance regarding this concern as no significant differences were observed between clopidogrel and aspirin in the incidence of MI or stent thrombosis during follow-up. These results suggest that, even in populations where CYP2C19 polymorphism prevalence may be higher, clopidogrel monotherapy remains a safe and effective option in the stabilized period following PCI [41, 42].

To test the consistency of our findings, we conducted interaction analyses for all subgroups. The treatment effect of clopidogrel was consistent across almost all patient populations, with the interaction tests being non-significant. This suggests that the observed benefit of clopidogrel is broadly generalizable across subgroups. Our subgroup analysis showed no significant differences between PPI use and the beneficial effect of clopidogrel, a finding strongly supported by the 2025 ACS guidelines [2]. The guidelines give a Class 1 A recommendation for PPI use in patients at risk of gastrointestinal bleeding. PPI prescription is often not a random event but represents a marker for patients with an elevated baseline risk of gastrointestinal complications, such as a history of peptic ulcer disease or prior gastrointestinal bleeding. Our pooled data confirms this at a meta-analytic level, demonstrating that the superiority of clopidogrel over aspirin is maintained even in the large proportion of patients who are appropriately prescribed concomitant PPI therapy for gastroprotection. Clopidogrel and certain PPIs share metabolic pathways via CYP2C19, raising concerns about drug–drug interactions. A double-blind RCT demonstrated that omeprazole did not elevate ischemic event rates relative to placebo in patients on clopidogrel but was effective in minimizing gastrointestinal bleeding [43]. Furthermore, post hoc evaluations from multiple RCTs have shown that the concomitant use of clinically necessary PPI with clopidogrel does not elevate ischemic risk [44]. Importantly, the therapeutic impact of ticagrelor and prasugrel is not significantly influenced by the concomitant administration of PPI [44, 45]. Thus, PPIs are recommended in ACS patients at high bleeding risk receiving DAPT or oral anticoagulants [2].

A key finding of our meta-analysis is the statistically significant reduction in the primary composite outcome of MACE and the secondary composite outcome of NACE with clopidogrel monotherapy, despite the individual components not reaching statistical significance in the main analysis. This well-described phenomenon is attributable to the increased statistical power of combining multiple, relatively infrequent endpoints.

The significant benefit in MACE was primarily driven by the consistent and clinically meaningful trends towards reducing both MI (16% relative risk reduction) and stroke (22% relative risk reduction). The effect was confirmed to be statistically significant within our subgroup analysis of RCTs in MI (RR: 0.67, 95% CI: 0.53–0.85). While these overall individual ischemic endpoints did not achieve statistical significance in their analysis, their cumulative effect was substantial enough to demonstrate a clear overall benefit for the composite outcome. This interpretation is strongly supported by our leave-one-out sensitivity analysis, in which the benefit of clopidogrel on MI and stroke became statistically significant after removing certain influential studies, indicating that the primary finding is robust. Furthermore, the significant reduction in subgroup analysis of MI regarding the RCTs that represent the highest quality of evidence in our analysis, comprising a large sample of patients (19,734), minimizes the potential for confounding inherent in observational studies.

In our subgroup analysis based on PCI complexity, the treatment effect of clopidogrel was consistent across patients with and without complex PCI, with no significant interaction detected for the primary outcome of MACE. This finding warrants discussion in the context of a recent meta-analysis by Oliva et al. [46], which also evaluated the role of antiplatelet therapy stratified by PCI complexity. In their analysis of five randomized trials, Oliva and colleagues reported a significant treatment interaction, demonstrating that P2Y12 inhibitor monotherapy after a short 1- to 3-month course of DAPT significantly reduced the risk of MI in patients with complex PCI when compared to standard ≥ 12-month DAPT.

Several key methodological differences between our analyses likely explain this discrepancy. Firstly, the comparator arms were different: our meta-analysis compares two monotherapies (clopidogrel vs. aspirin) for long-term maintenance, whereas the Oliva et al. analysis compared a P2Y12 inhibitor monotherapy strategy against a more potent standard DAPT regimen [46]. Consequently, their findings support an early aspirin withdrawal strategy, demonstrating a clinical benefit driven primarily by a reduction in bleeding without an ischemic consequence in the complex PCI population. In contrast, our study addresses the separate clinical question of selecting the optimal long-term monotherapy therapy for secondary prevention after a standard course of DAPT has already been completed. Furthermore, the analysis by Oliva et al. included trials of potent P2Y12 inhibitors like ticagrelor, while our study focused on clopidogrel, and this difference in therapy potency could also contribute to their observed interaction. Thus, the findings of our meta-analyses should be seen as complementary, addressing different but equally important clinical questions in the management of patients after PCI.

A large IPD meta-analysis of seven RCTs with several patients was 29,000, further supporting our findings, demonstrating that clopidogrel monotherapy reduced MACE compared to aspirin. While the IPD analysis provides high internal validity and focuses on a broad population with established coronary artery disease (CAD), our study specifically targeted patients undergoing PCI, incorporating a substantially larger and more heterogeneous cohort (> 162,000 patients), including observational data.

An examination of the Kaplan-Meier curve for the primary composite outcome MACE revealed a clinically meaningful pattern of benefit for clopidogrel. The survival curves for the clopidogrel and aspirin groups began to separate early, within the first year of follow-up, and this divergence continued to widen consistently throughout the entire follow-up period. This observation is significant for two reasons. First, the early separation suggests that the therapeutic advantage of clopidogrel over aspirin is not a delayed effect but manifests relatively soon after the switch to monotherapy. Second, the continuous and steady divergence, rather than a sudden change, indicates a persistent and cumulative benefit of clopidogrel in preventing long-term prevention of MACE over several years. This pattern of early and sustained separation, like that observed in landmark trials like HOST-EXAM and SMART-CHOICE 3 [12, 15], strengthens the validity of our main result and implies that the 21% risk decrease reflects a consistent clinical impact.

In our meta-regression results, the coefficient of 0.04 reflects a 4% relative increase in the risk ratio for MACE per one-year increment in mean age, indicating a progressive elevation in cardiovascular risk with advancing age, independent of antiplatelet therapy [47].

### Strengths and limitations

The major strength of this meta-analysis was the comprehensive subgroup analysis across multiple clinically relevant factors that have not been addressed in previous meta-analyses [14, 39, 48–51]. Our findings have important clinical relevance. While both the 2023 AHA/ACC chronic coronary disease guidelines and 2024 ESC chronic coronary syndrome guidelines recommend antiplatelet monotherapy as standard care following DAPT completion, neither provides a definitive recommendation favoring either aspirin or clopidogrel between aspirin versus clopidogrel for long-term maintenance therapy [3, 52]. This meta-analysis fills an evidence gap in the chronic maintenance phase, supporting clopidogrel as a more effective option than aspirin.

Furthermore, this meta-analysis exclusively included studies in which patients were treated with clopidogrel or aspirin monotherapy only after completion of a standard course of DAPT following PCI. In contrast, the IPD meta-analysis by Giacoppo et al. [8], which had to statistically manage dissimilar baseline risks arising from varied DAPT durations across trials, also included other P2Y₁₂ inhibitors (ticagrelor or prasugrel). Focusing only on clopidogrel versus aspirin initiated post-DAPT, our analysis offers a more robust and clinically relevant comparison for this population’s most common secondary prevention.

Finally, a notable strength of our analysis is the inclusion of extended long-term follow-up data, with Kaplan–Meier cumulative incidence curves extending up to 10 years. This prolonged observation period enables a more robust evaluation of the sustained efficacy and safety of clopidogrel monotherapy, including the detection of late-occurring events and potential attenuation of therapeutic benefit over time, factors that were not sufficiently addressed in previous meta-analyses [48].

Although our findings are supported by multiple analyses, some limitations should be noted. The main one is the moderate-to-high heterogeneity in certain outcomes, arising from variations in study design, as both RCTs and observational studies were included. This may introduce selection bias from residual confounding. The inclusion of observational data was necessary, as the limited number of RCTs alone would have reduced statistical power and generalizability. Importantly, subgroup analysis of RCTs showed results consistent with the main findings, reinforcing the robustness of our conclusions.

Heterogeneity also stemmed from differences in patient populations and clinical settings—ranging from ACS to stable CAD—across diverse healthcare systems and follow-up periods (12 months to > 5 years). Another source was the variation in DAPT duration before monotherapy, from short-term (1–6 months) to standard-term (≥ 6–12 months).

Multiple subgroup analyses, sensitivity analysis, and meta-regression were employed to address these sources of heterogeneity, such statistical approaches can only partially adjust for underlying heterogeneity. As such, while our findings consistently favor clopidogrel in the primary outcome, the estimated effect size should be interpreted as an average across diverse clinical scenarios, rather than a definitive effect applicable to all subgroups.

Moreover, another primary limitation of this meta-analysis is its geographic scope, which impacts the generalizability of the findings. All included studies were conducted exclusively in East Asian populations. This is important due to known differences in pharmacogenomics, such as the higher prevalence of CYP2C19 loss-of-function alleles that affect clopidogrel metabolism, and potential variations in clinical practice patterns [53]. Consequently, clinical practice patterns may differ, including variations in the typical patient risk profiles, the prevalence of PPI co-prescription, and preferences in stent platforms. Therefore, while our findings provide robust evidence for the comparison of clopidogrel and aspirin monotherapy in East Asian patients (6 from South Korea, 2 from Japan, and 2 from China), resulting in limited ethnic representativeness and caution is strongly warranted when extrapolating these conclusions to non-East Asian populations, such as Western cohorts, where the pharmacogenomic and clinical context may differ substantially. Therefore, caution is strongly warranted when extending our conclusions to non-East Asian populations such as Western cohorts. Moreover, women were underrepresented across the included trials, comprising only 18–27% of the participants. This limits the transportability of our findings, as sex-specific differences in thrombotic/bleeding risk profiles and potential variations in treatment responses to antiplatelet agents cannot be confidently excluded. Also, numerous subgroups and meta-regression analyses are susceptible to false-positive findings. we adjusted the p-values for interaction tests using the BH-FDR method. Despite this statistical correction, these analyses should be considered exploratory.

Furthermore, the lack of patient-level data precluded granular analyses on the potential subgroups who might drive the most benefit from clopidogrel monotherapy. A methodological limitation of this meta-analysis is the synthesis of time-to-event data. For the primary outcome (MACE), HRs were used to account for the time-dependent nature of events. However, not all studies reported HRs for secondary outcomes. To avoid reporting bias from excluding these data, RRs were used instead. Pooling RRs from studies with different follow-up durations may not fully reflect time-to-event patterns and could increase heterogeneity; thus, these findings should be interpreted cautiously.

## Conclusion

Clopidogrel monotherapy following standard DAPT was associated with a reduced risk of MACE compared to aspirin monotherapy. This benefit was not accompanied by a statistically significant increase in the risk of all-cause mortality and major bleeding. Validating these findings in large-scale trials that include more geographically and ethnically diverse patient populations remains a crucial area for future research.

## Supplementary Information

Below is the link to the electronic supplementary material.

Supplementary Material 1

## Data Availability

All data are available and attached.

## Abbreviations

ACS: Acute Coronary Syndrome
BMI: body mass index
CI: Confidence Interval
CKD: Chronic Kidney Disease
DAPT: Dual Antiplatelet Therapy
DM: Diabetes mellitus
DES: Drug-Eluting Stent
HR: Hazard Ratio
LVEF: left ventricular ejection fraction
MACE: Major Adverse Cardiovascular Events
MI: Myocardial Infarction
NACE: Net Adverse Clinical Events
NSTEMI: Non–Non-ST-Elevation Myocardial Infarction
PCI: Percutaneous Coronary Intervention
PPI: Proton Pump Inhibitors
P2Y12: P2Y12 Inhibitor (a class of antiplatelet drugs)
RCTs: Randomized Clinical Trials
RR: Risk Ratio
RFI: Reverse Fragility Index
SAPT: Single Antiplatelet Therapy
STEMI: ST-Elevation Myocardial Infarction
TLR: Target Lesion Revascularization
TVR: Target Vessel Revascularization
