# Supplementary material for: Clopidogrel versus aspirin monotherapy following dual antiplatelet therapy after percutaneous coronary intervention: an updated meta-analysis of 162,829 patients
Source: Eur J Clin Pharmacol. 2026 Feb 6;82(3):64. doi: 10.1007/s00228-025-03942-3 (PMC12881014; doi:10.1007/s00228-025-03942-3)
Supplement: Supplementary file 1 — Supplementary Material 1 [file 228_2025_3942_MOESM1_ESM.docx]

**Contents**

[Supplementary Figure 1: Risk of bias assessment of included studies using RoB2: 3](#_Toc210624672)

[Supplementary Figure 2: Forest plot of major adverse cardiovascular events (MACE) based on risk ratio (RR) estimates 4](#_Toc210624673)

[Supplementary Figure 3: Schoenfeld residuals plot 5](#_Toc210624674)

[Supplementary Figure 4: log-log curve 6](#_Toc210624675)

[Supplementary Figure 5: Forest plot for net adverse clinical events (NACE) 7](#_Toc210624676)

[Supplementary Figure 6: Forest plot for all-cause mortality 8](#_Toc210624677)

[Supplementary Figure 7: Forest plot for cardiovascular mortality 9](#_Toc210624678)

[Supplementary Figure 8: Forest plot for myocardial infarction (MI) 10](#_Toc210624679)

[Supplementary Figure 9: Forest plot for stent thrombosis 11](#_Toc210624680)

[Supplementary Figure 10: Forest plot for any revascularization 12](#_Toc210624681)

[Supplementary Figure 11: Forest plot for target lesion revascularization (TLR) 13](#_Toc210624682)

[Supplementary Figure 12: Forest plot for target vessel revascularization (TVR) 14](#_Toc210624683)

[Supplementary Figure 13: Forest plot for stroke 15](#_Toc210624684)

[Supplementary Figure 14: Forest plot for ischemic stroke 16](#_Toc210624685)

[Supplementary Figure 15: Forest plot for hemorrhagic stroke 17](#_Toc210624686)

[Supplementary Figure 16: Forest plot for all bleeding 17](#_Toc210624687)

[Supplementary Figure 17: Forest plot for gastrointestinal bleeding 18](#_Toc210624688)

[Supplementary Figure 18: Forest plot for intracranial bleeding 19](#_Toc210624689)

[Supplementary Figure 19: Funnel plot for MACE 20](#_Toc210624690)

[Supplementary Figure 20: Trim and fill plot for MACE 21](#_Toc210624691)

[Supplementary Figure 21: Funnel plot for major bleeding 22](#_Toc210624692)

[Supplementary Figure 22: Trim and fill plot for major bleeding 23](#_Toc210624693)

[Supplementary Figure 23: leave-one-out sensitivity analysis for major adverse cardiovascular events (MACE) 24](#_Toc210624694)

[Supplementary Figure 24: leave-one-out sensitivity analysis for major bleeding 25](#_Toc210624695)

[Supplementary Figure 25: leave-one-out sensitivity analysis for all-cause mortality 26](#_Toc210624696)

[Supplementary Figure 26: leave-one-out sensitivity analysis for cardiovascular mortality 27](#_Toc210624697)

[Supplementary Figure 27: leave-one-out sensitivity analysis for stent thrombosis 28](#_Toc210624698)

[Supplementary Figure 28: leave-one-out sensitivity analysis for any revascularization 29](#_Toc210624699)

[Supplementary Figure 29: leave-one-out sensitivity analysis for TVR 30](#_Toc210624700)

[Supplementary Figure 30: leave-one-out sensitivity analysis for TLR 31](#_Toc210624701)

[Supplementary Figure 31: leave-one-out sensitivity analysis for haemorrhagic stroke 32](#_Toc210624702)

[Supplementary Figure 32: leave-one-out sensitivity analysis for gastrointestinal bleeding 33](#_Toc210624703)

[Supplementary Figure 33: leave-one-out sensitivity analysis for intracranial bleeding 34](#_Toc210624704)

[Supplementary Figure 34: leave-one-out sensitivity analysis for MI 35](#_Toc210624705)

[Supplementary Figure 35: leave-one-out sensitivity analysis for stroke 36](#_Toc210624706)

[Supplementary Figure 36: leave-one-out sensitivity analysis for Ischaemic stroke 37](#_Toc210624707)

[Supplementary Figure 37: leave-one-out sensitivity analysis for all bleeding 38](#_Toc210624708)

[Supplementary Figure 38: leave-one-out sensitivity analysis for NACE 39](#_Toc210624709)

[Supplementary Figure 39: Bubble plot of meta-regression of significant covariate (Age) on MACE 40](#_Toc210624710)

[Supplementary Figure 40: Bubble plot of meta-regression of significant covariate (Hypertension) on MACE 41](#_Toc210624711)

[Supplementary Figure 41: Bubble plot of meta-regression of significant covariate (LVEF) on MACE 42](#_Toc210624712)

[Supplementary Table 1: Search strategy for each database 43](#_Toc210624713)

[Supplementary Table 2: Outcome definitions, and inclusion and exclusion criteria for each study 45](#_Toc210624714)

[Supplementary Table 3A: Baseline patient characteristics of included studies 52](#_Toc210624715)

[Supplementary Table 3B: Baseline patient characteristics of included studies 55](#_Toc210624716)

[Supplementary Table 4: meta-regression of major adverse cardiovascular events (MACE) 58](#_Toc210624717)

## Supplementary Figure 1: Risk of bias assessment of included studies using RoB2:

**A) Quality assessment according to risk of bias for each study.**


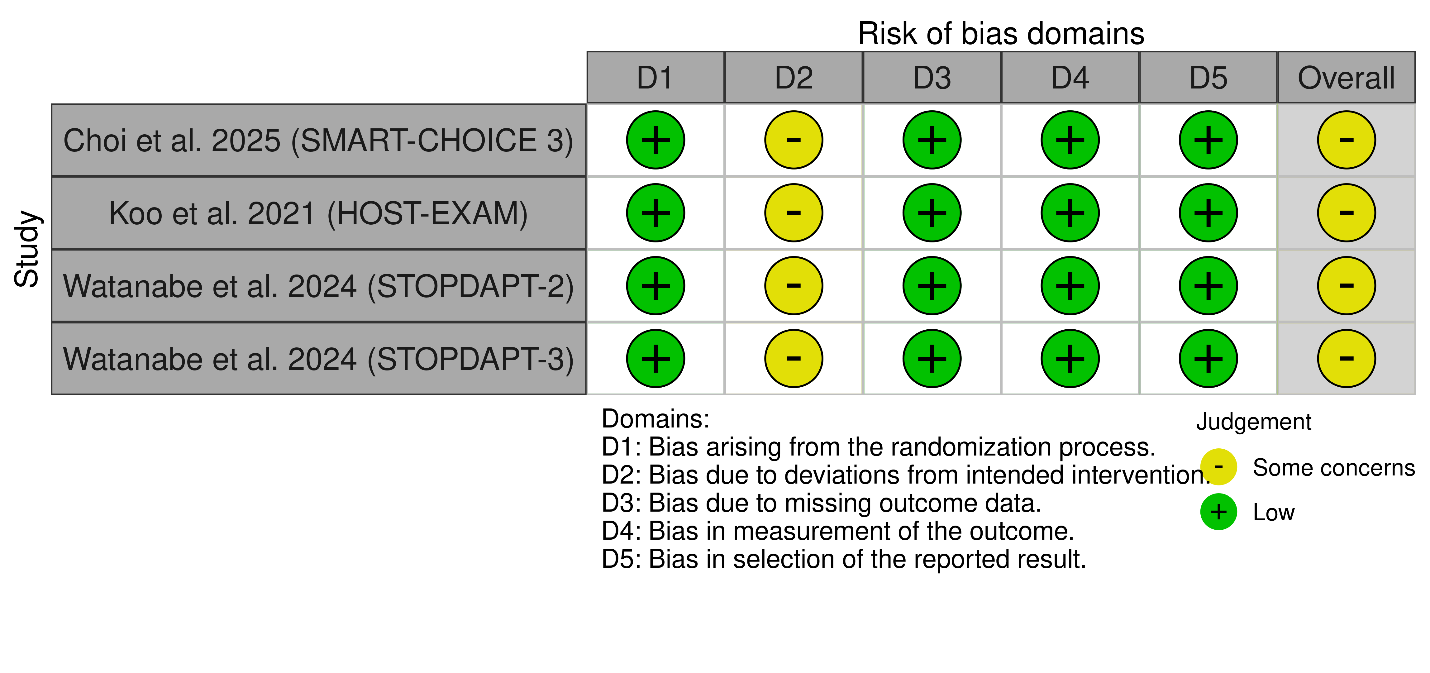


**B) Quality assessment according to risk of bias as percentage (intention to treat).**


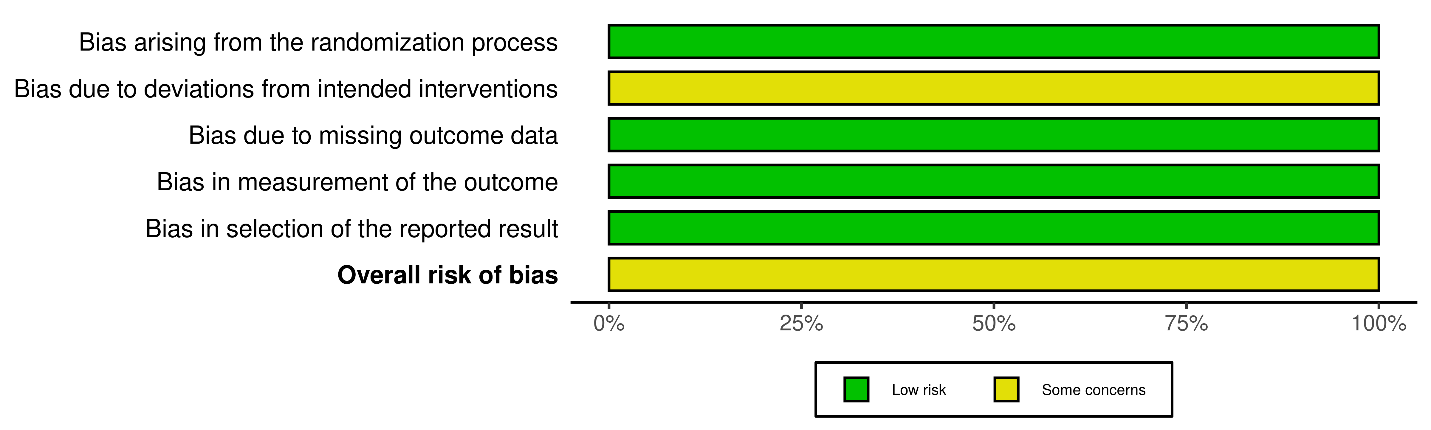


## Supplementary Figure 2: Forest plot of major adverse cardiovascular events (MACE) based on risk ratio (RR) estimates


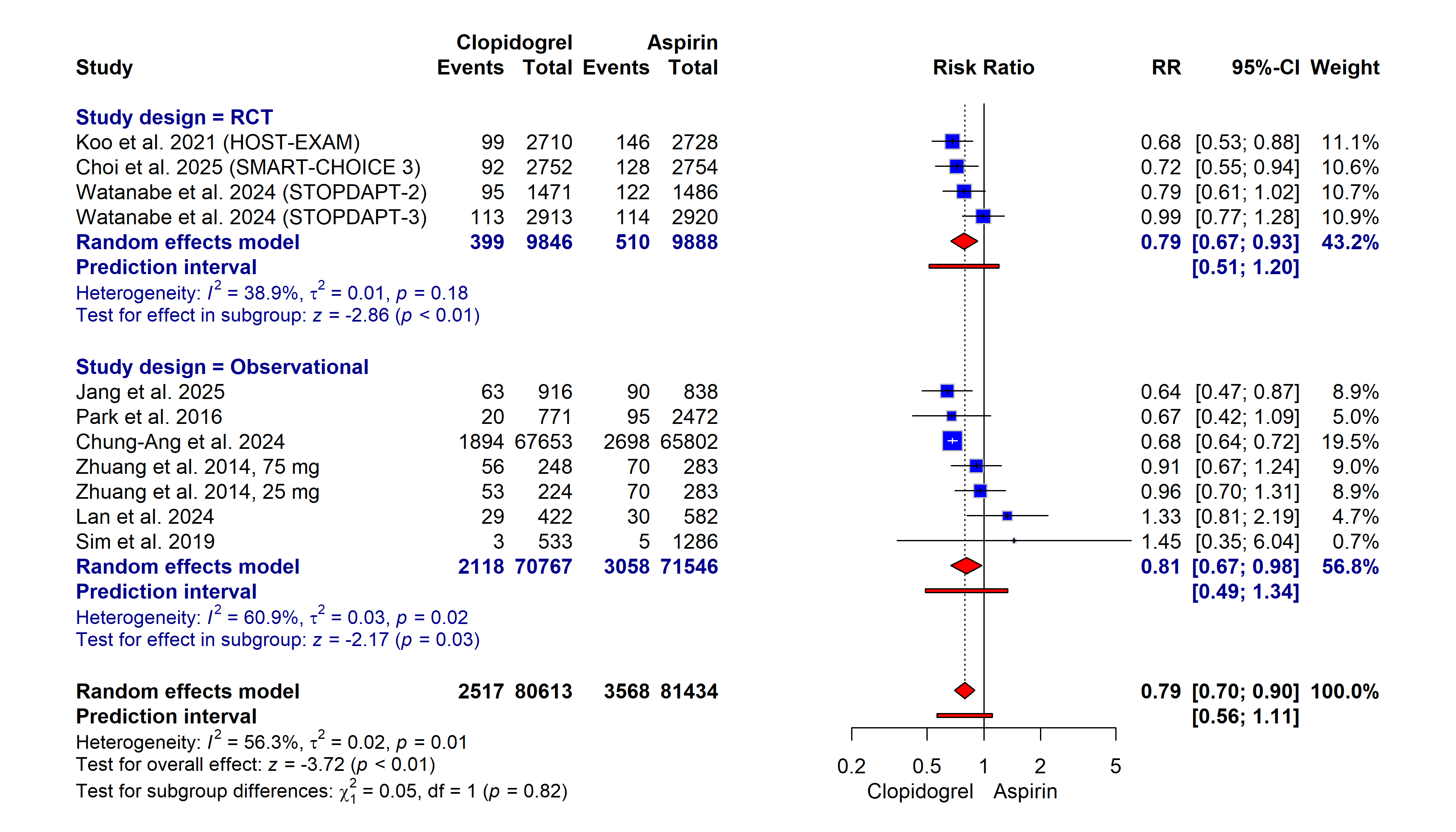


## Supplementary Figure 3: Schoenfeld residuals plot


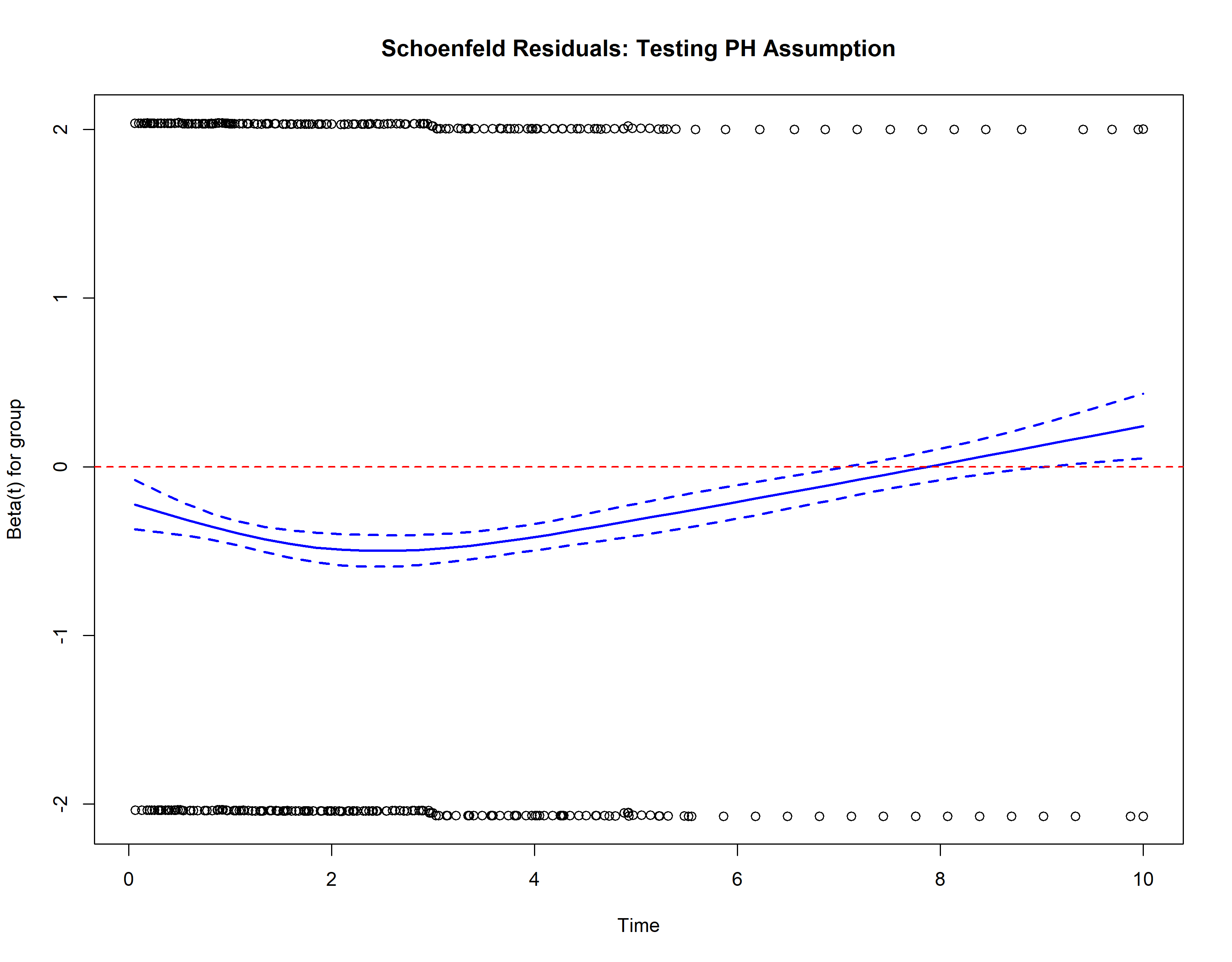


## Supplementary Figure 4: log-log curve


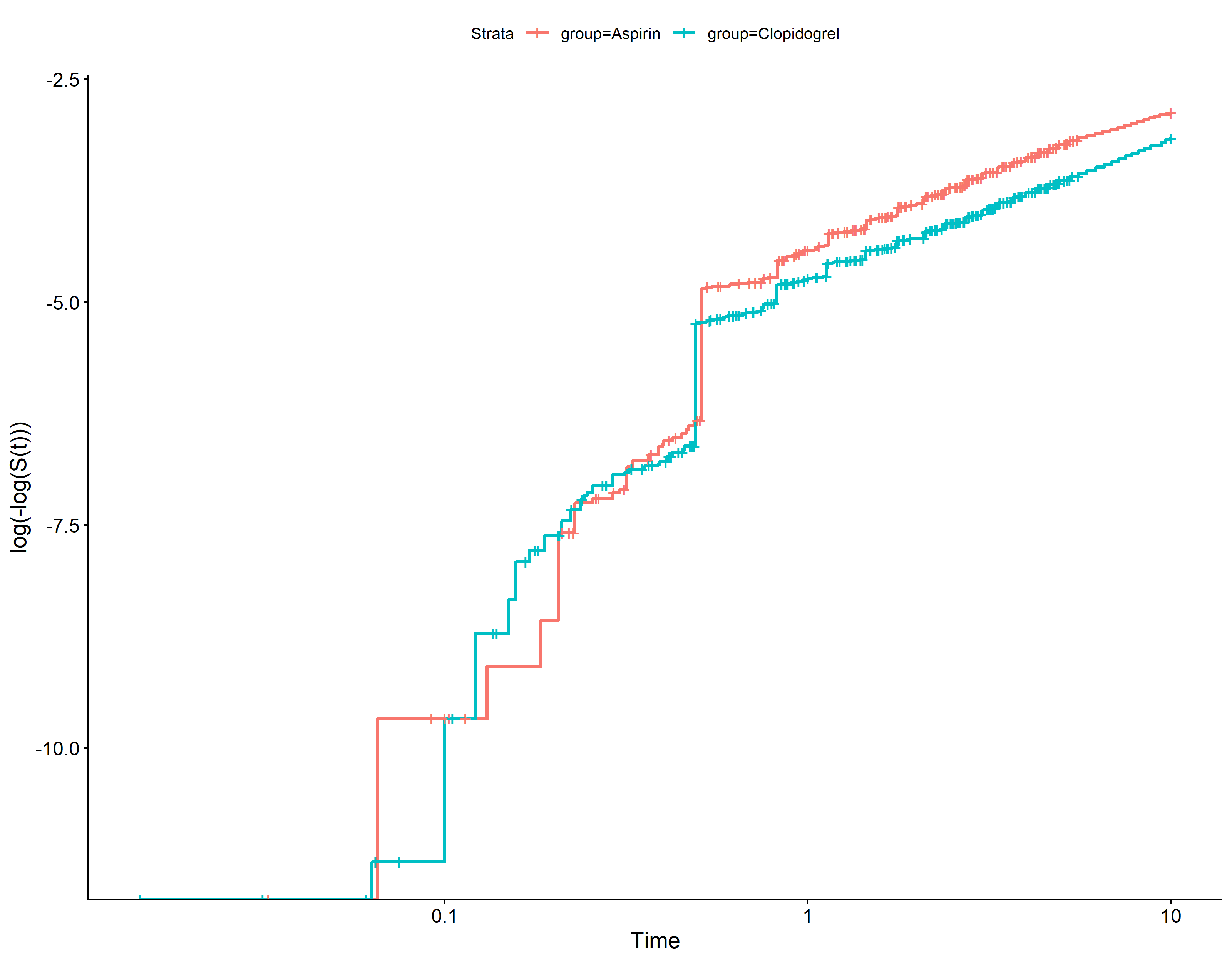


## Supplementary Figure 5: Forest plot for net adverse clinical events (NACE)


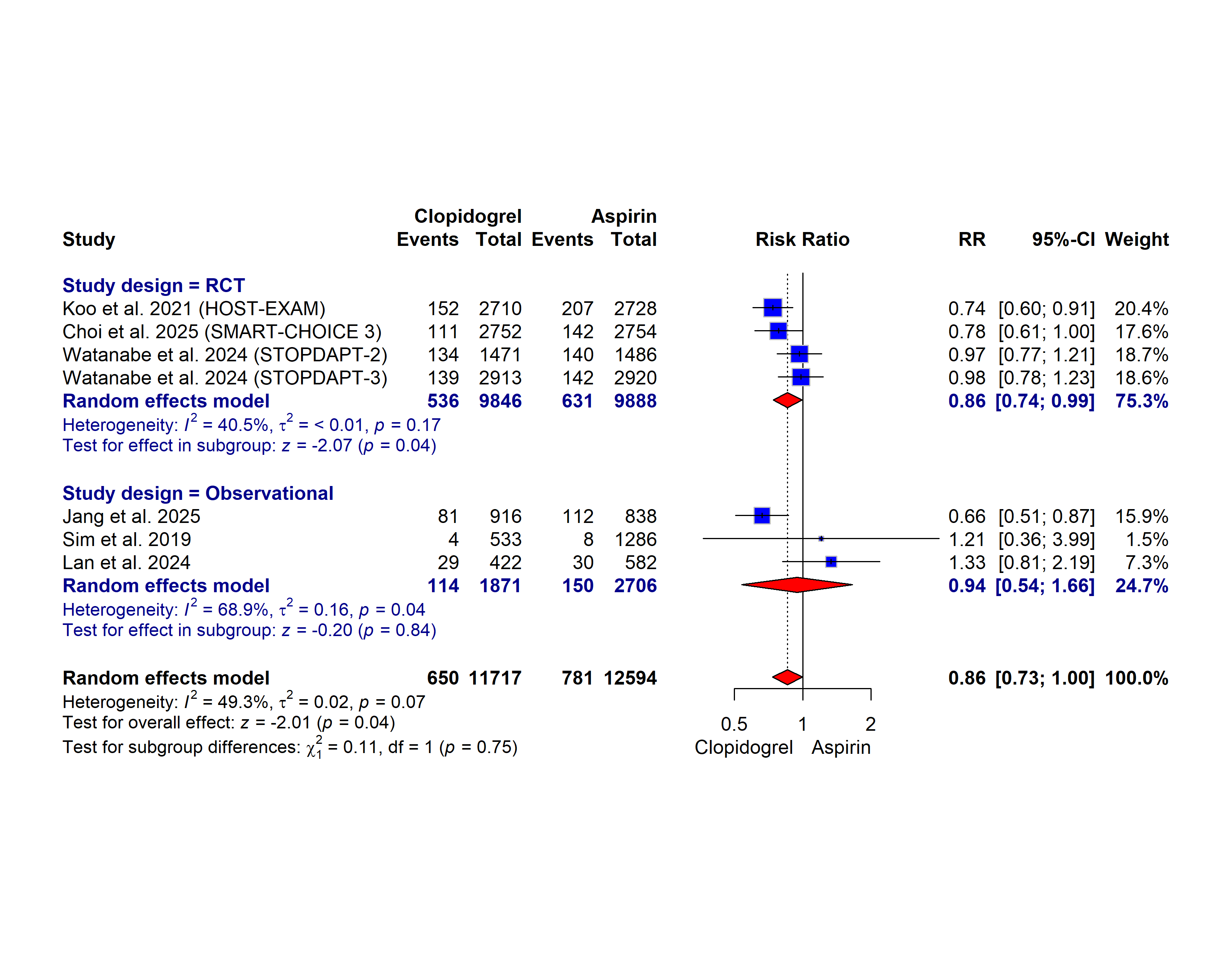


## Supplementary Figure 6: Forest plot for all-cause mortality


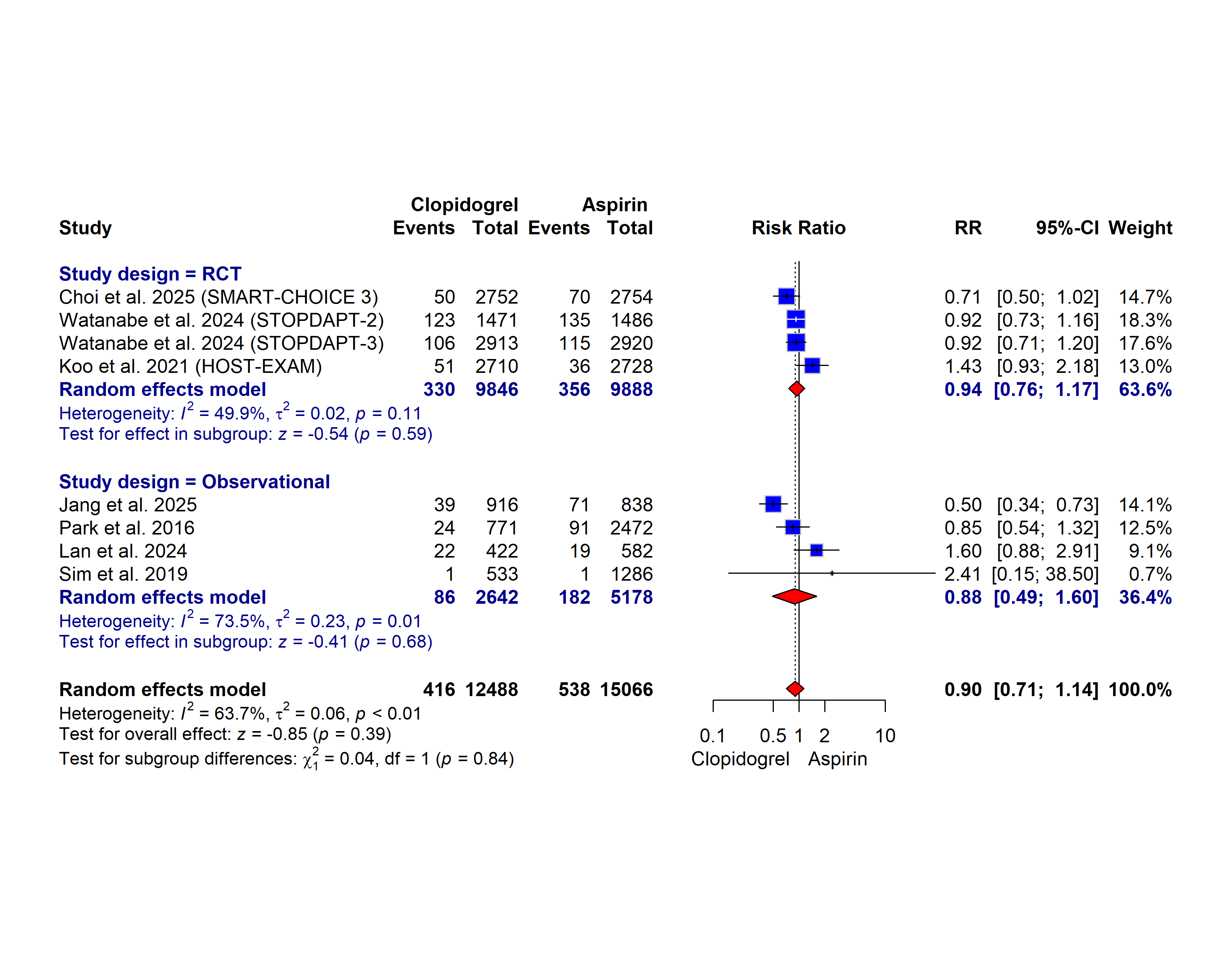


## Supplementary Figure 7: Forest plot for cardiovascular mortality


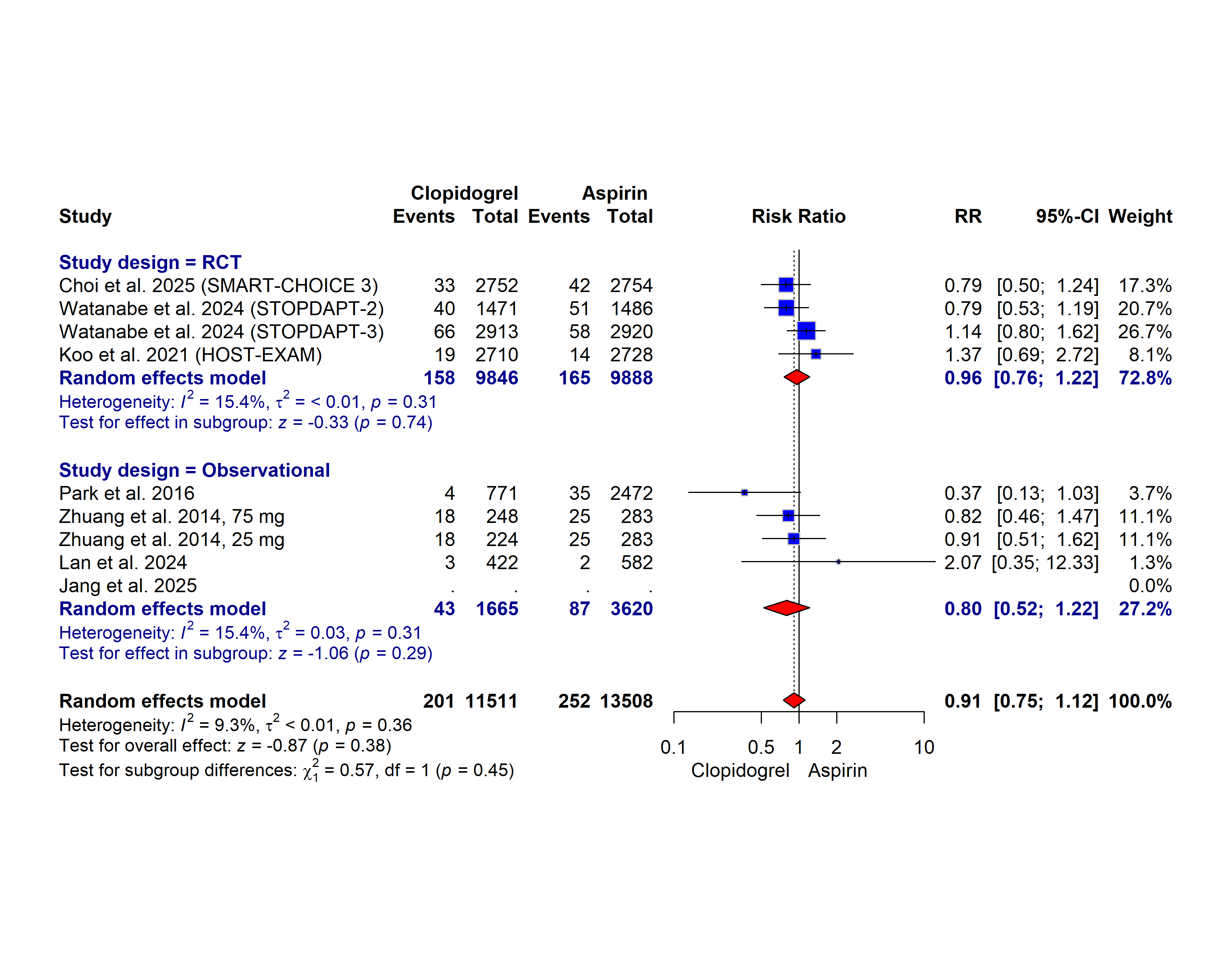


## Supplementary Figure 8: Forest plot for myocardial infarction (MI)


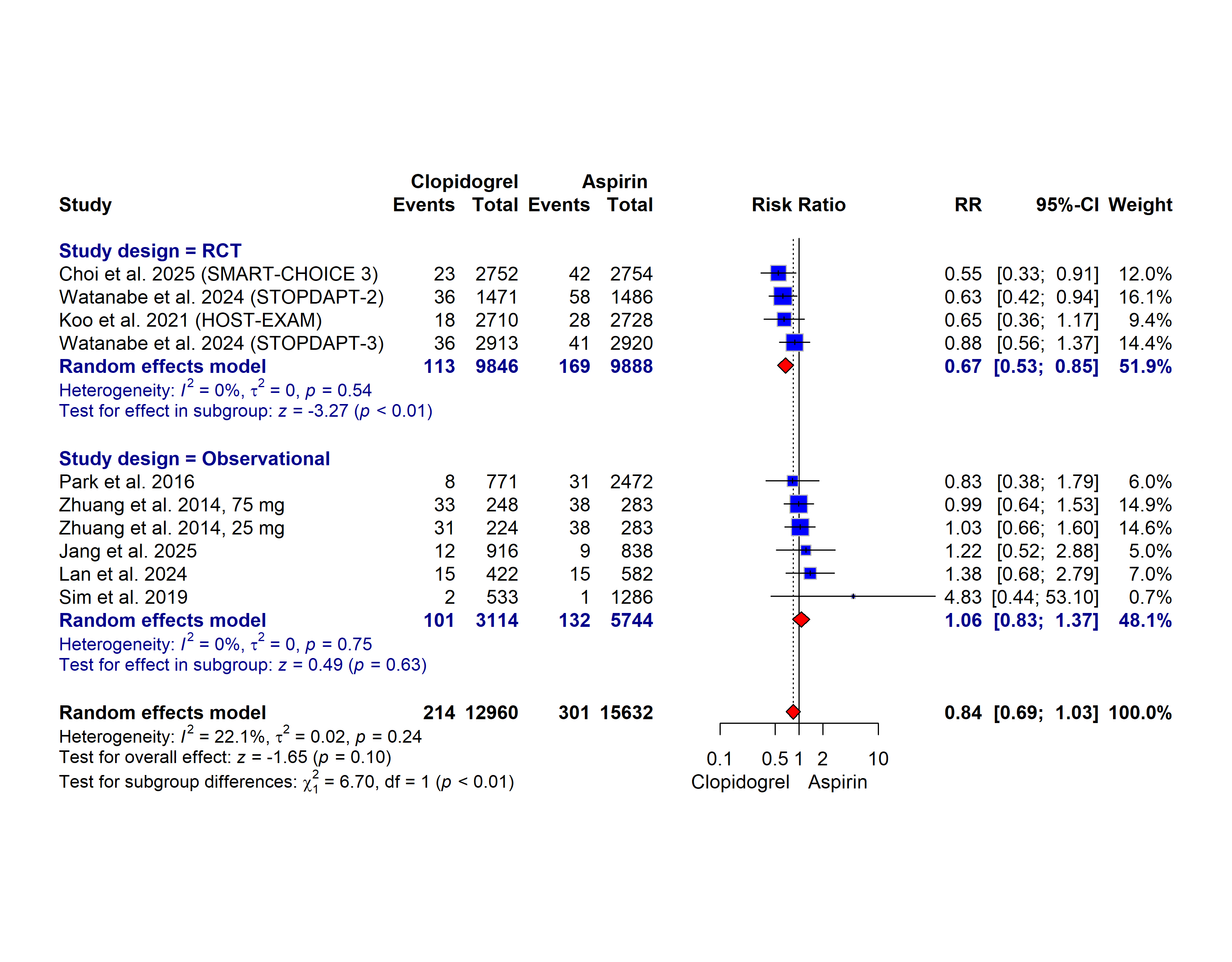


## Supplementary Figure 9: Forest plot for stent thrombosis


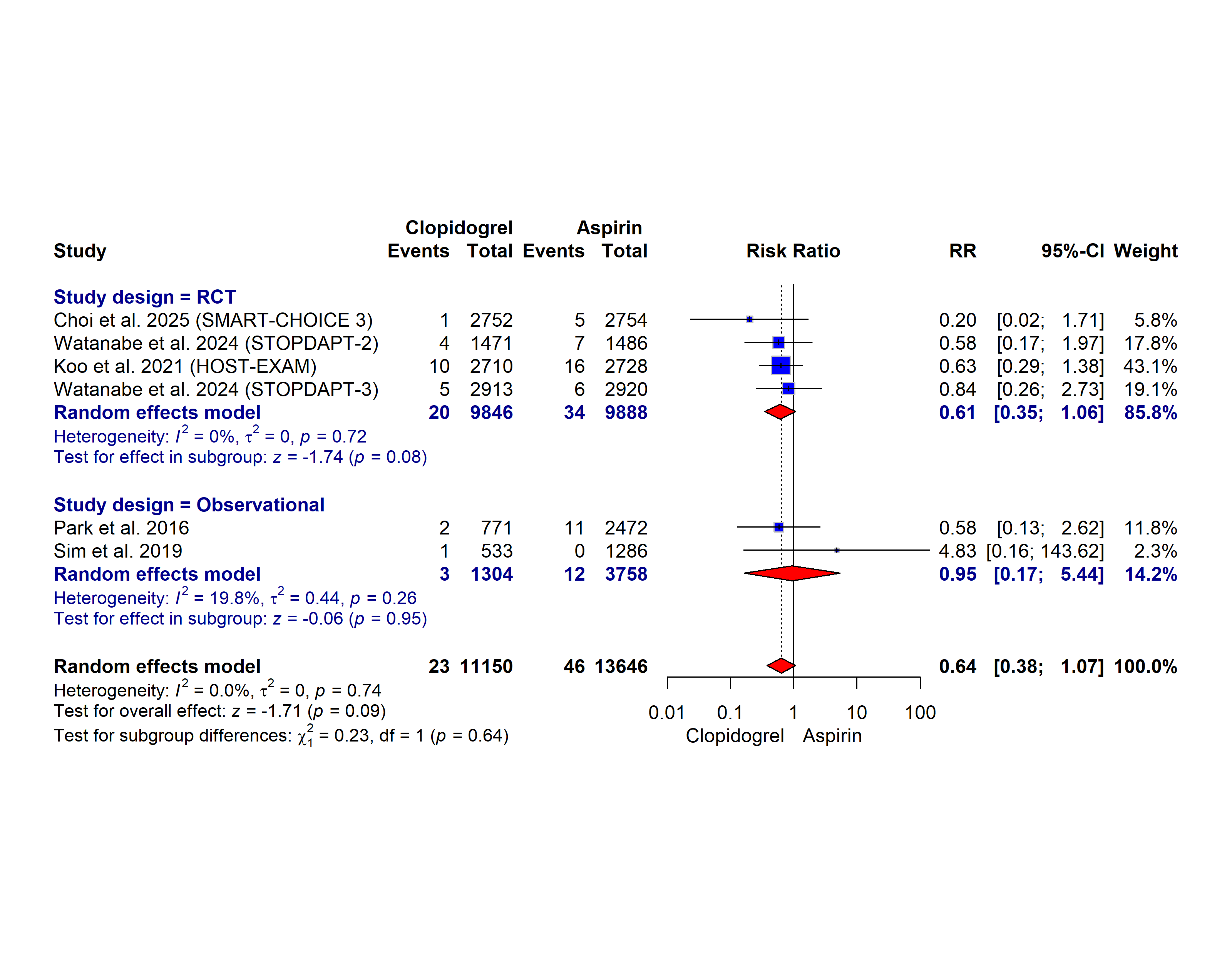


## Supplementary Figure 10: Forest plot for any revascularization


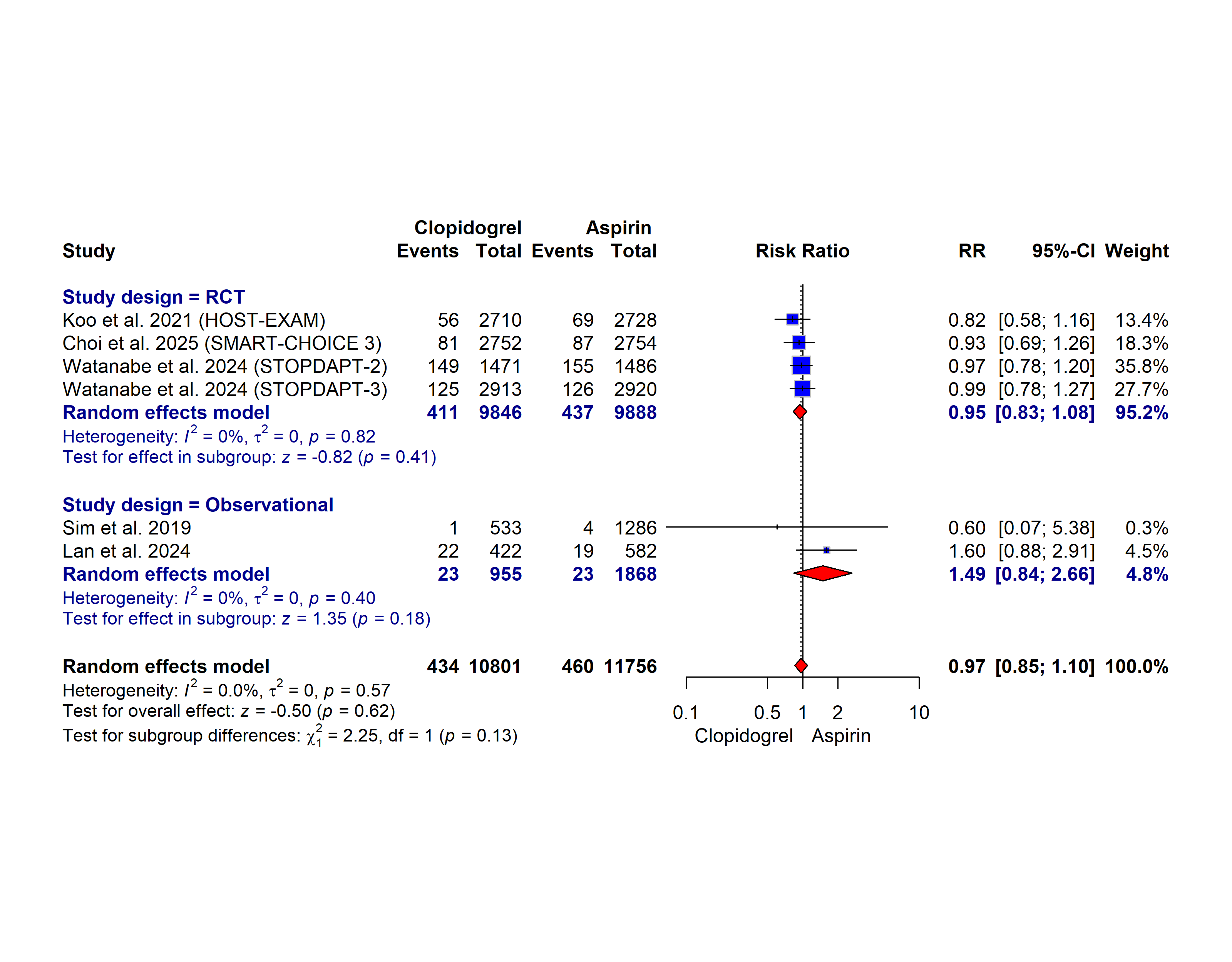


## Supplementary Figure 11: Forest plot for target lesion revascularization (TLR)


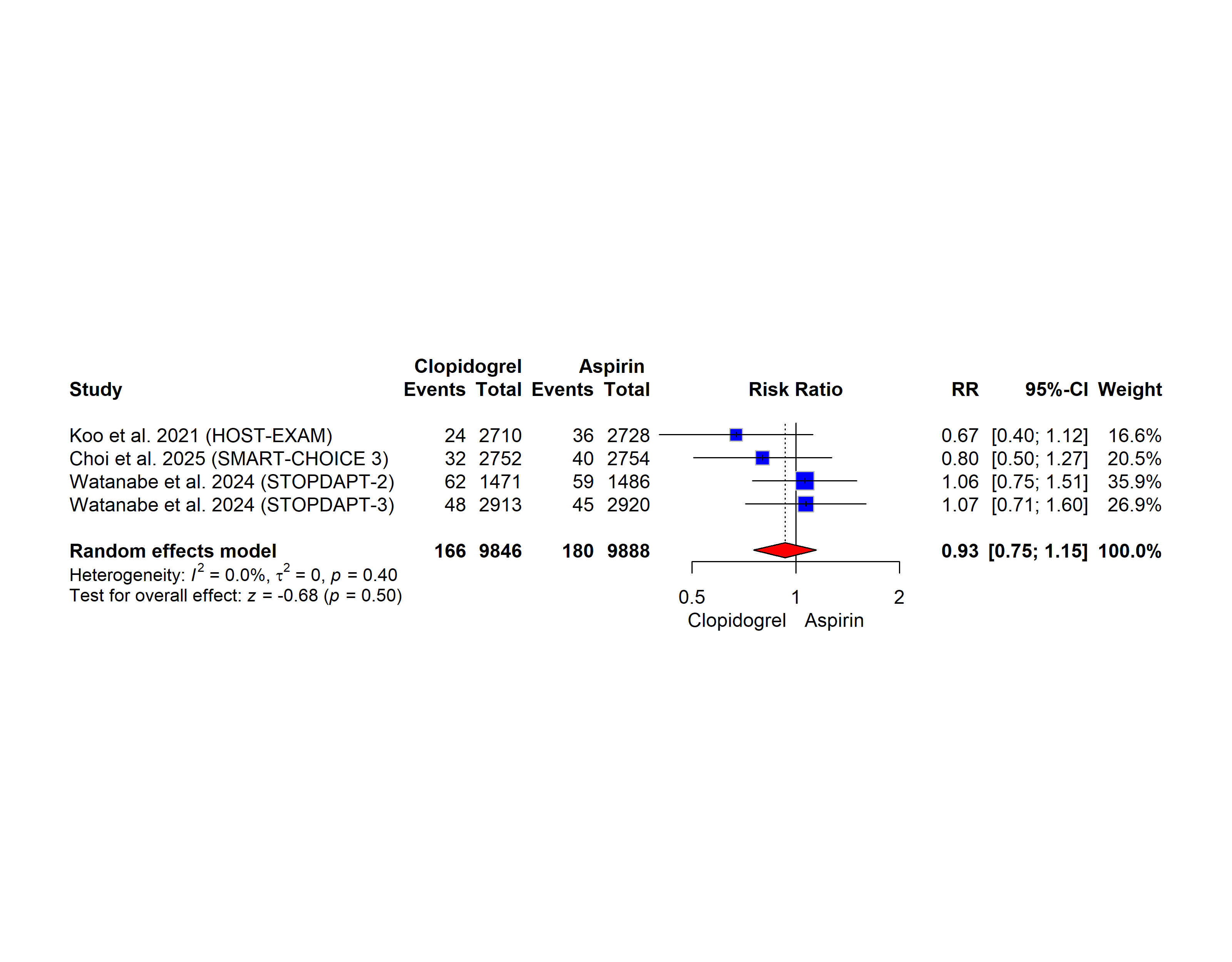


## Supplementary Figure 12: Forest plot for target vessel revascularization (TVR)


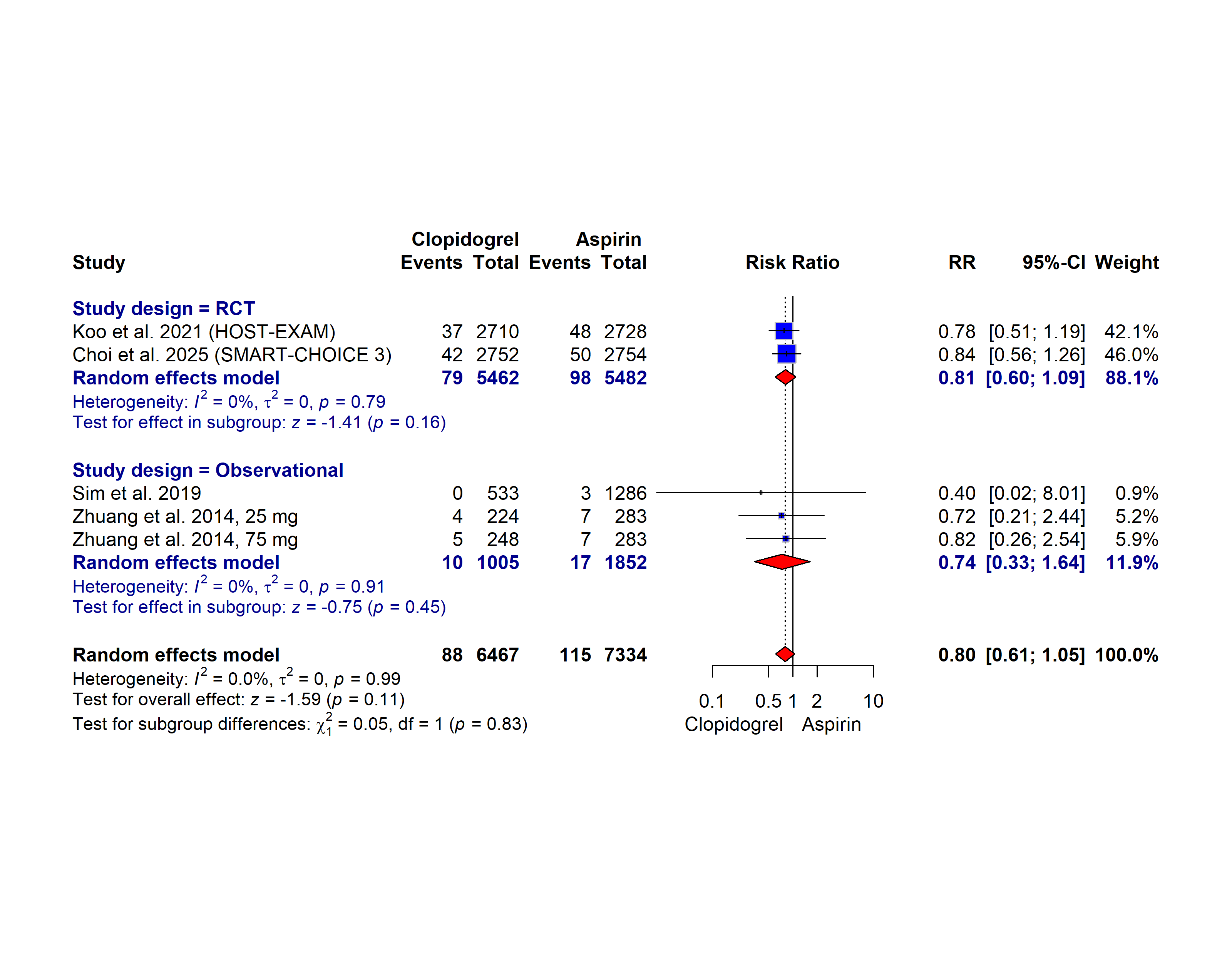


## Supplementary Figure 13: Forest plot for stroke


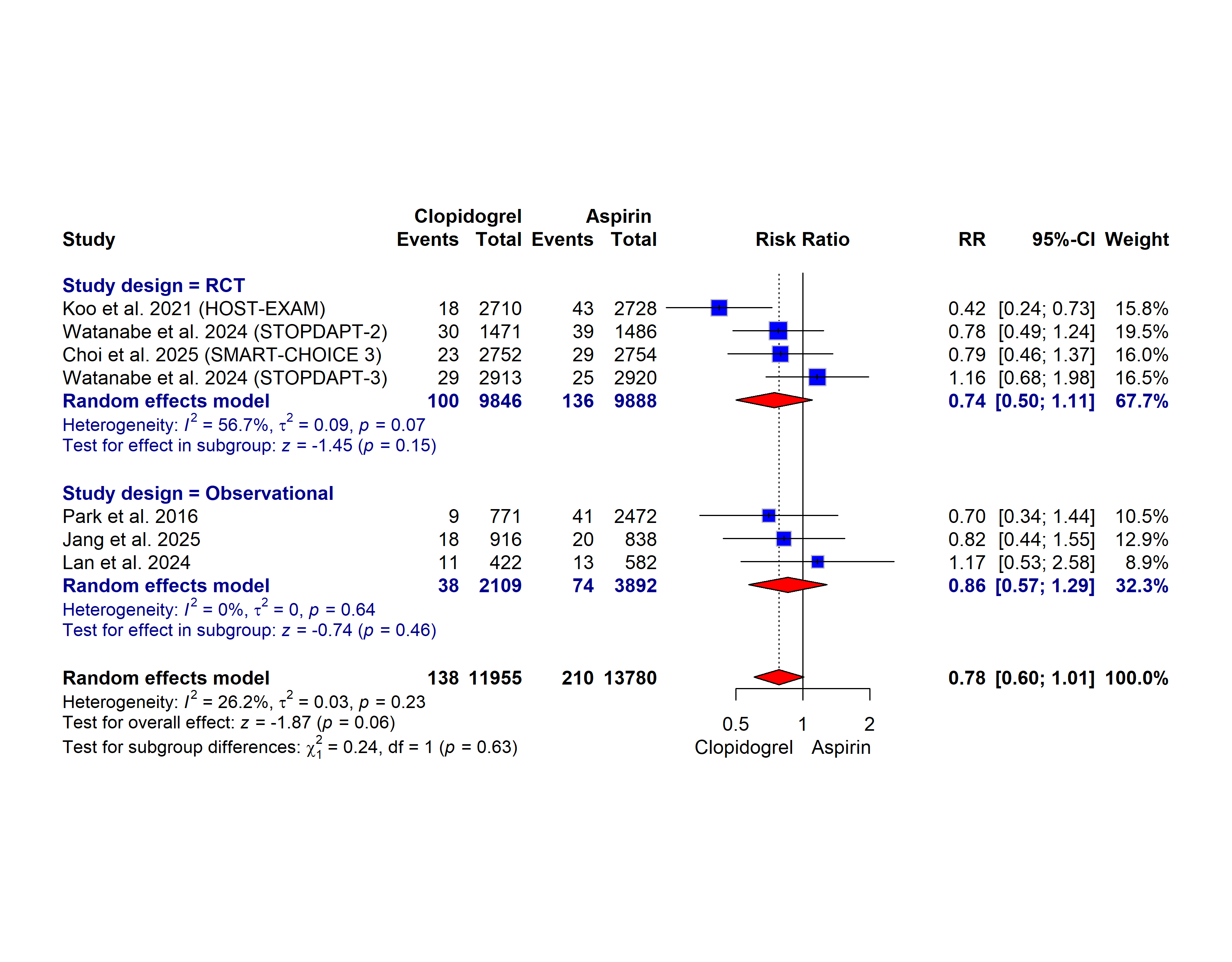


## Supplementary Figure 14: Forest plot for ischemic stroke


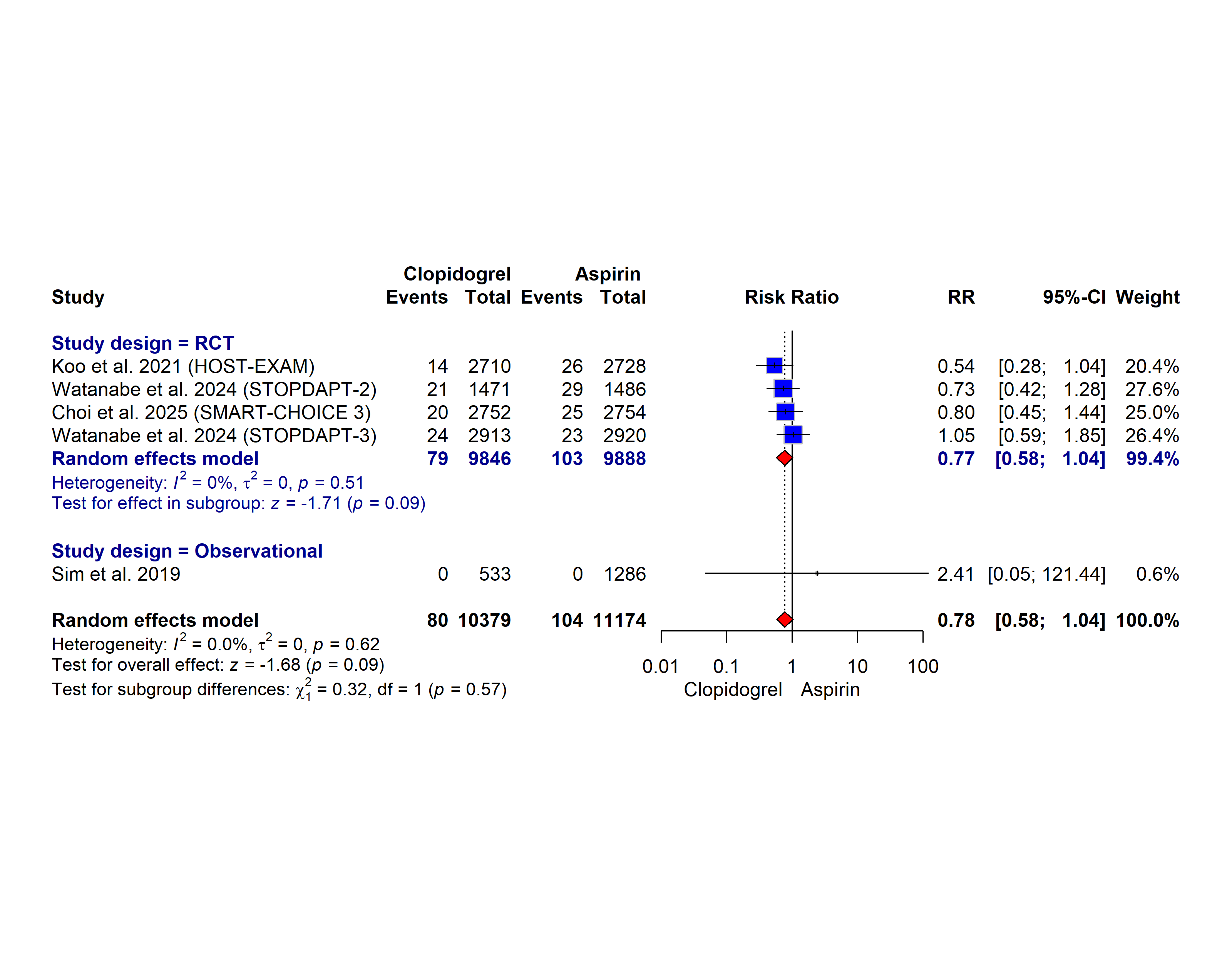


## Supplementary Figure 15: Forest plot for hemorrhagic stroke


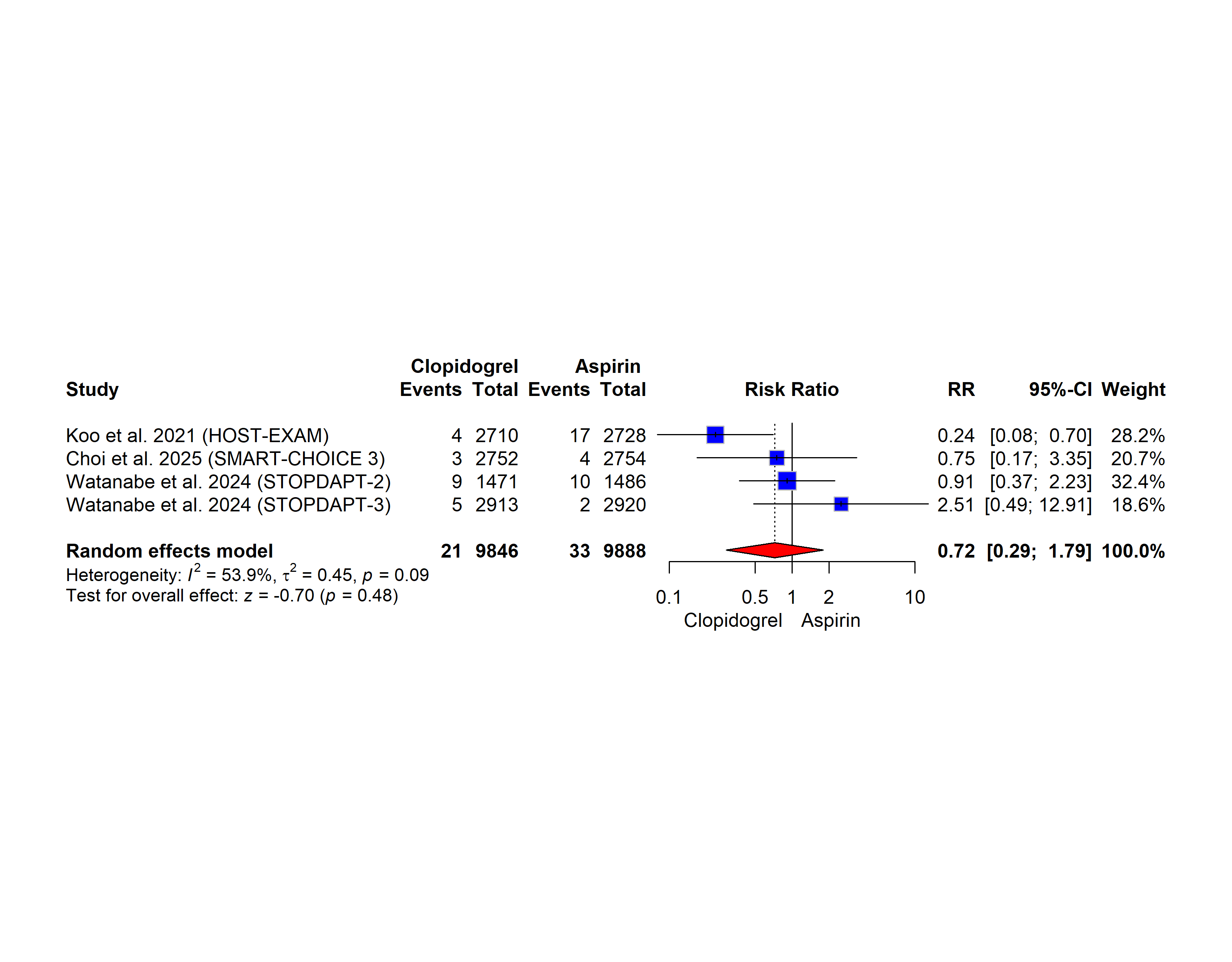


## Supplementary Figure 16: Forest plot for all bleeding


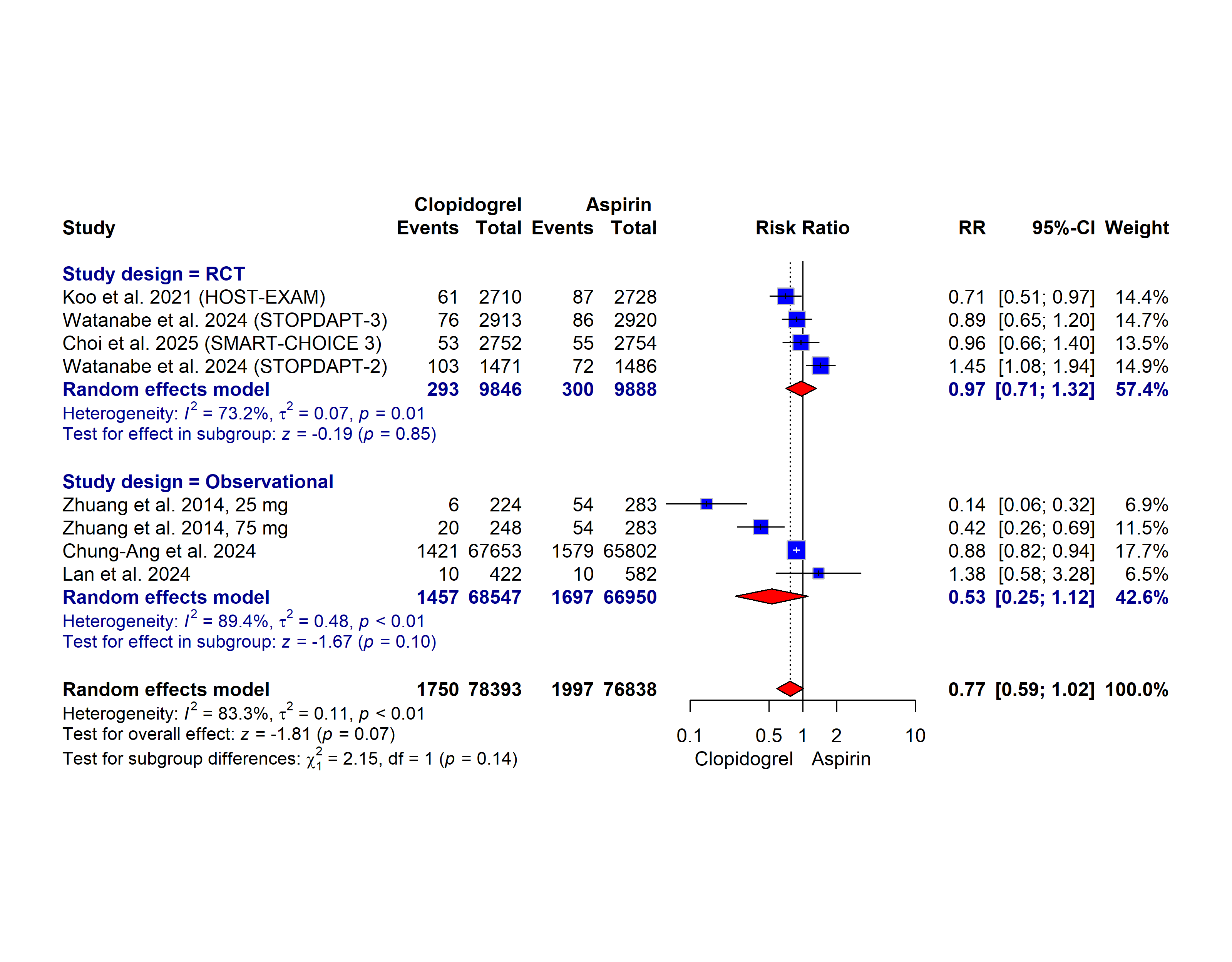


## Supplementary Figure 17: Forest plot for gastrointestinal bleeding


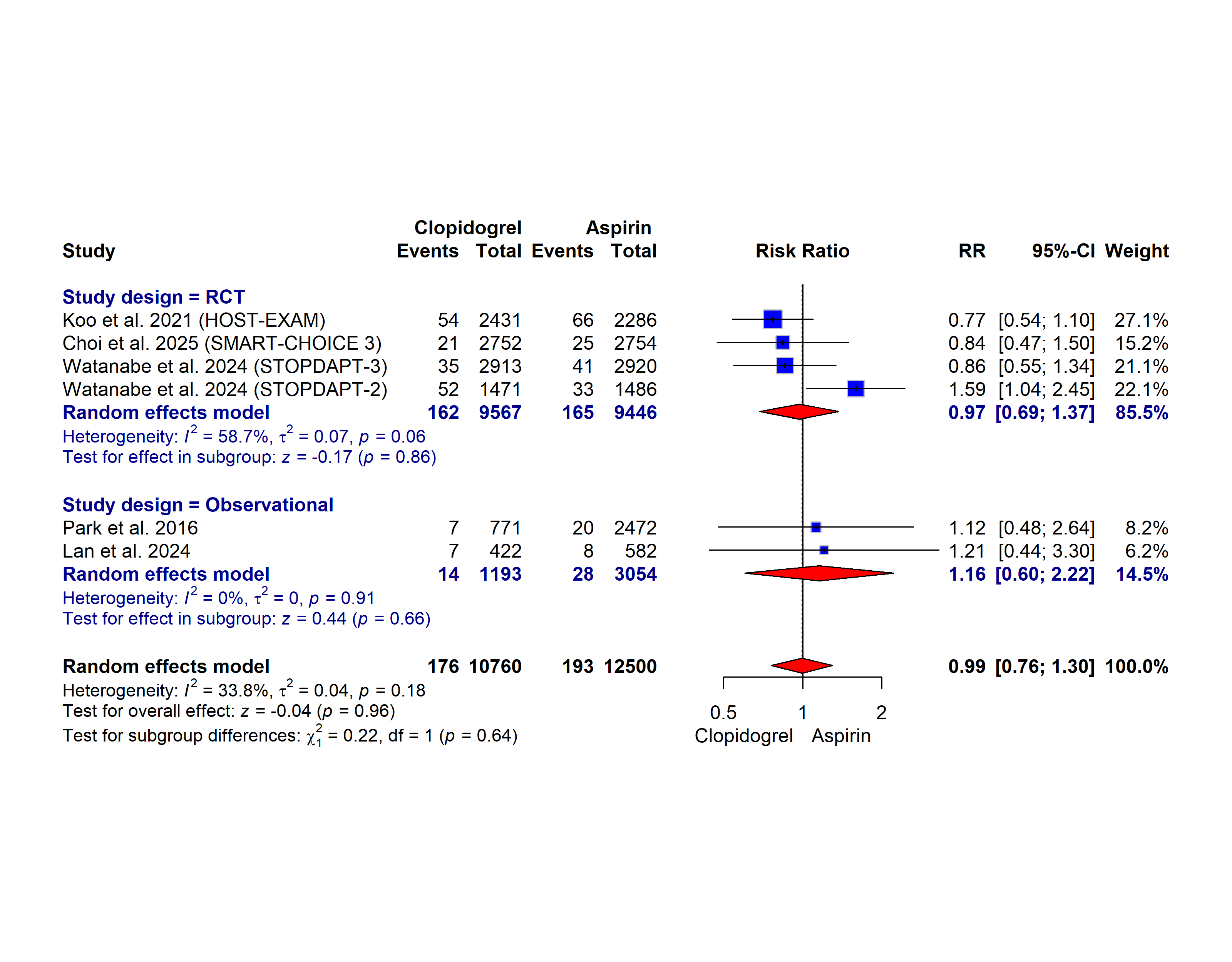


## Supplementary Figure 18: Forest plot for intracranial bleeding


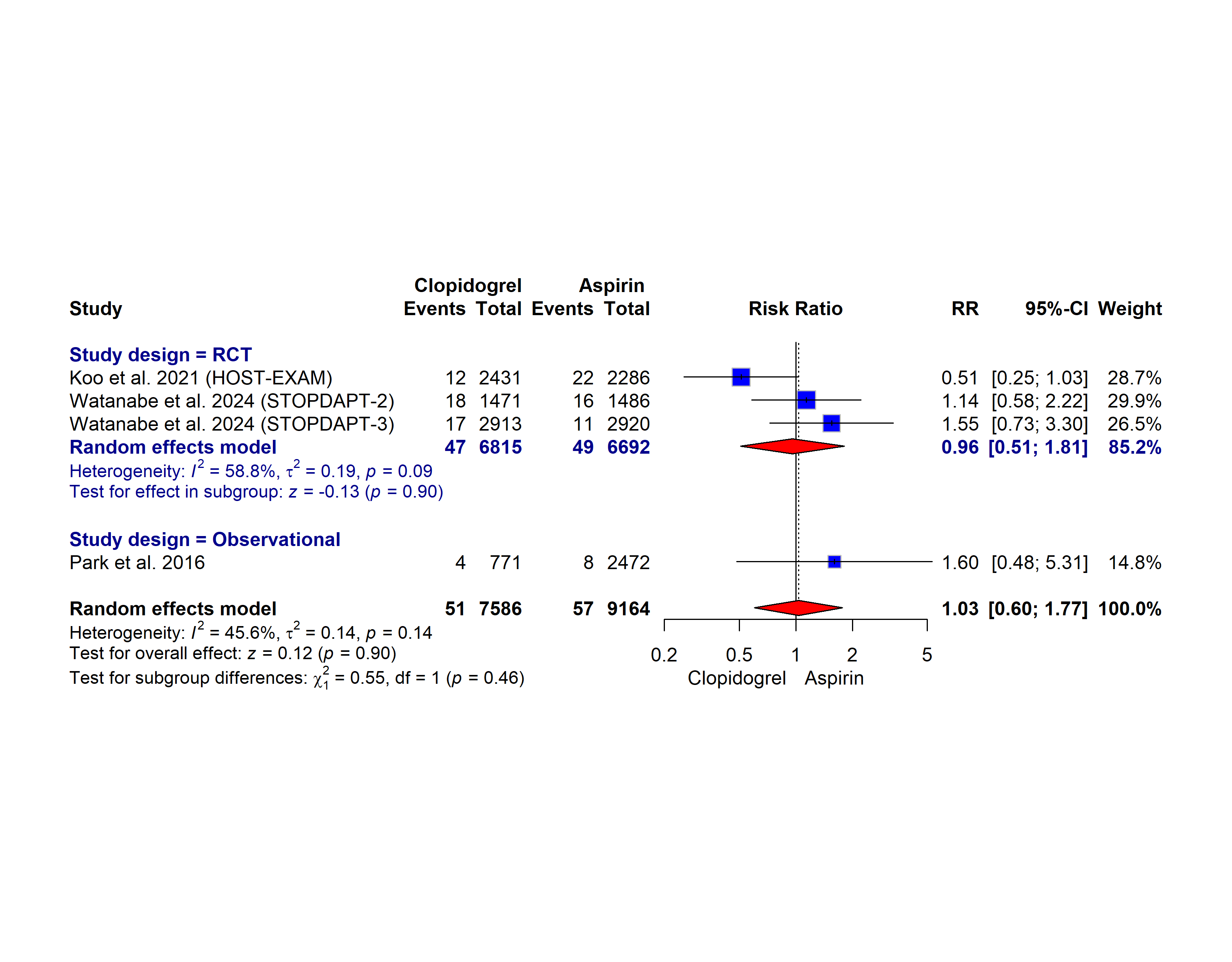


## Supplementary Figure 19: Funnel plot for MACE


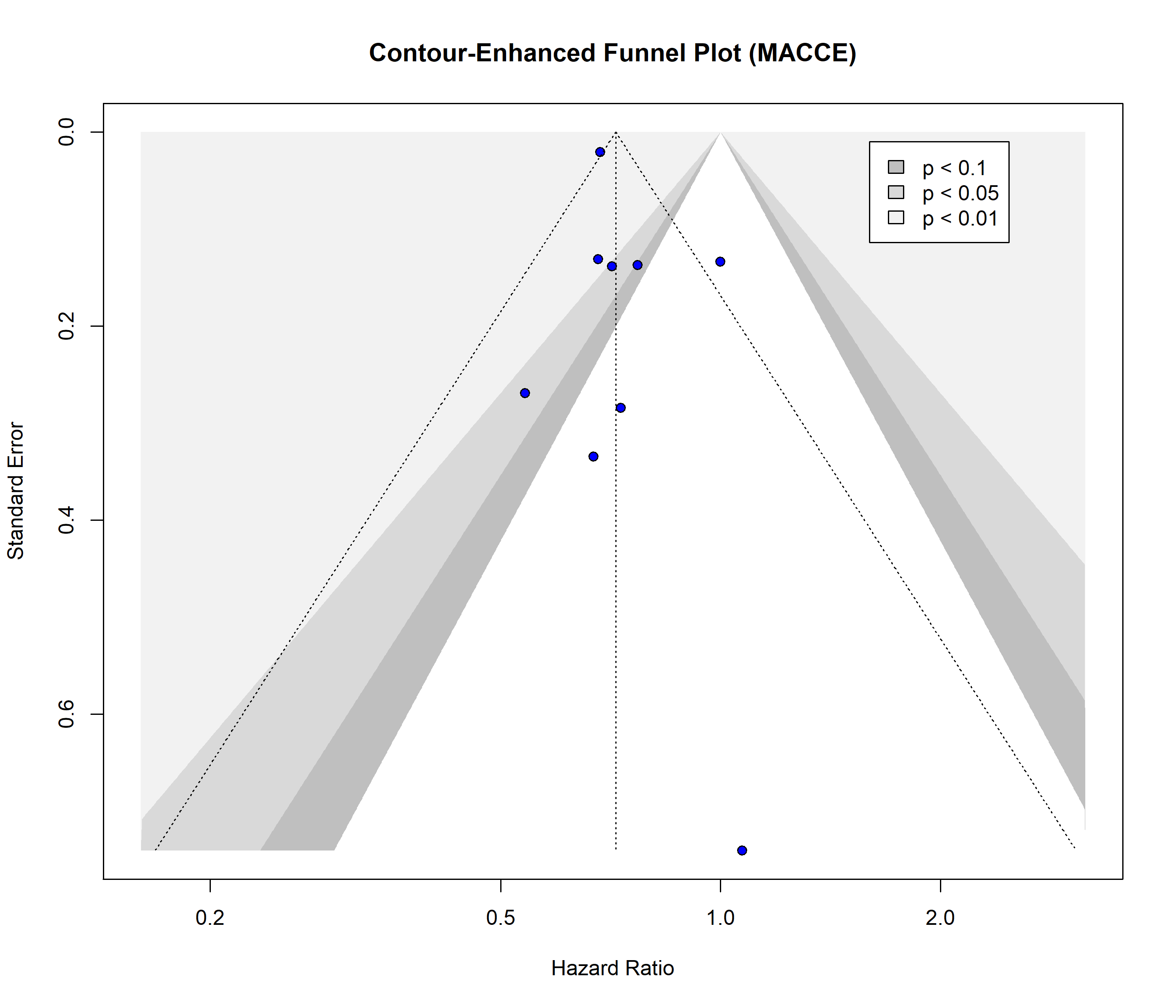


## Supplementary Figure 20: Trim and fill plot for MACE


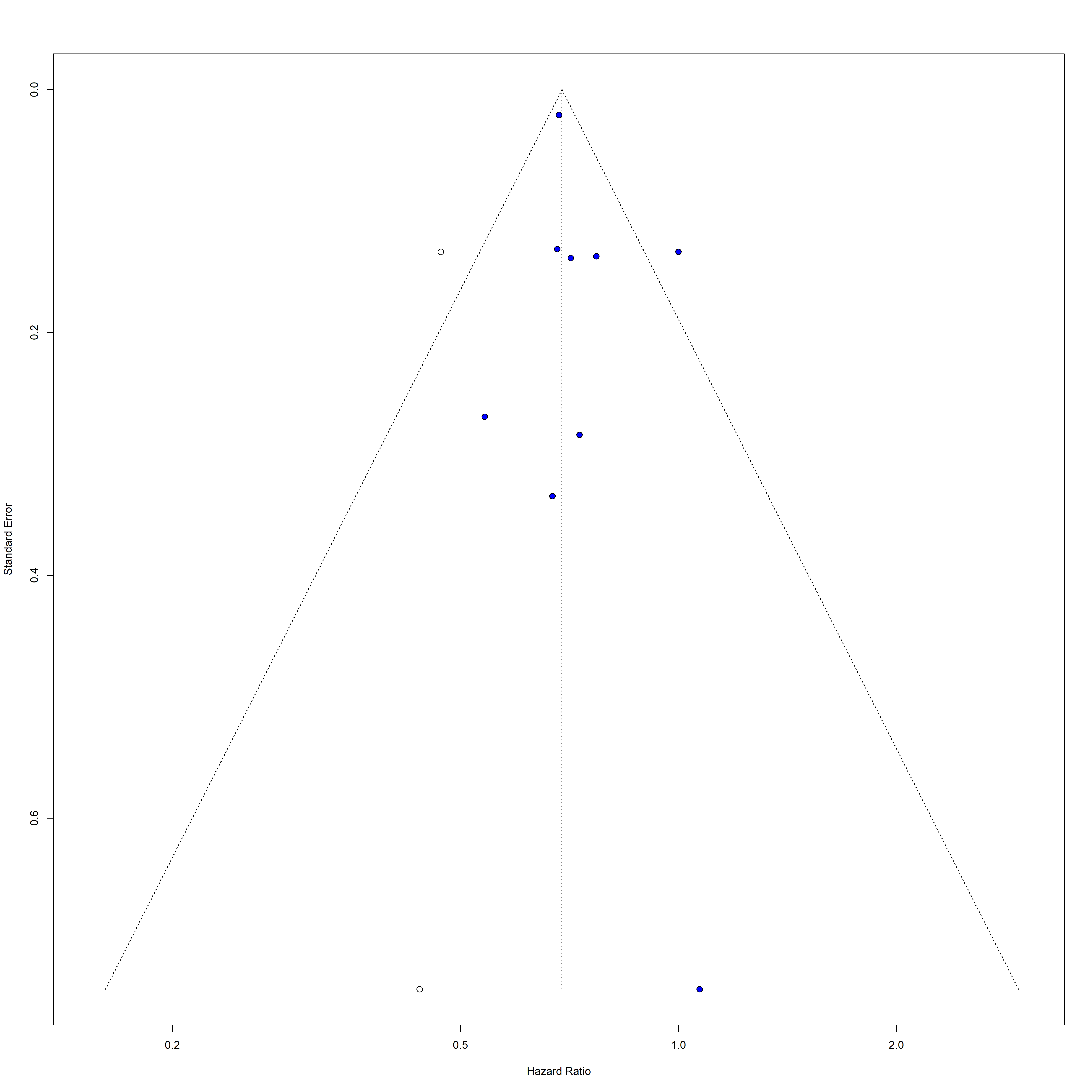


## Supplementary Figure 21: Funnel plot for major bleeding


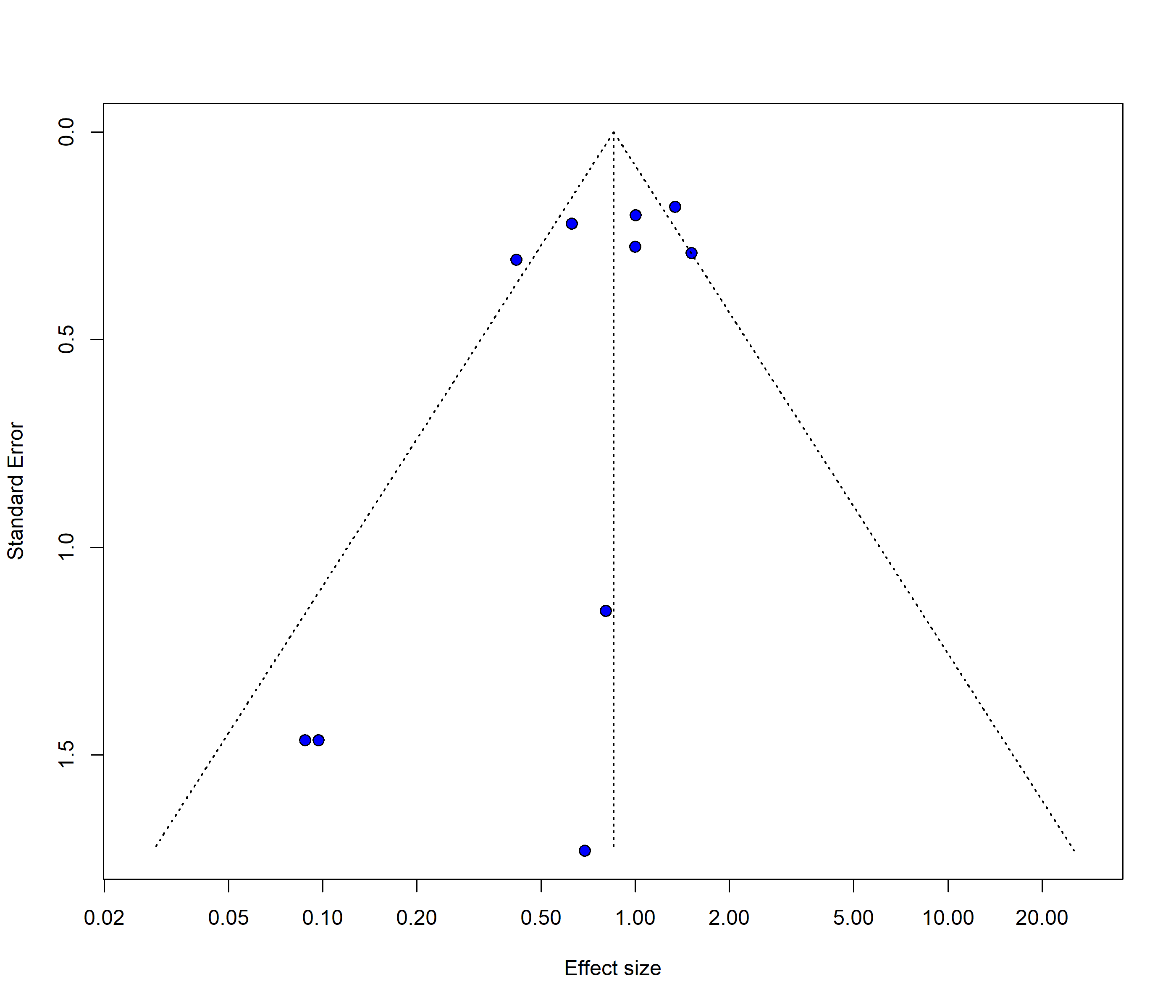


## Supplementary Figure 22: Trim and fill plot for major bleeding


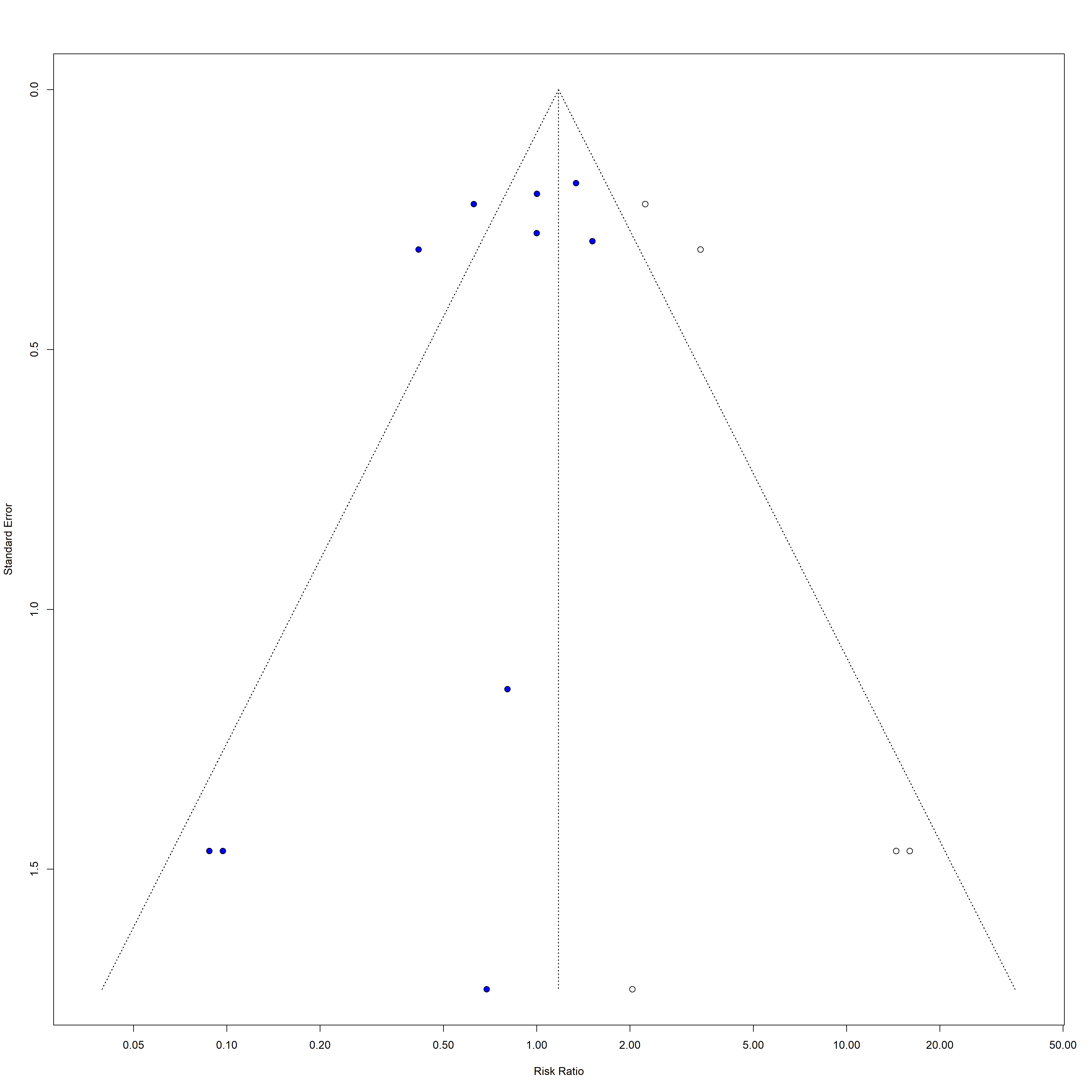


## Supplementary Figure 23: leave-one-out sensitivity analysis for major adverse cardiovascular events (MACE)


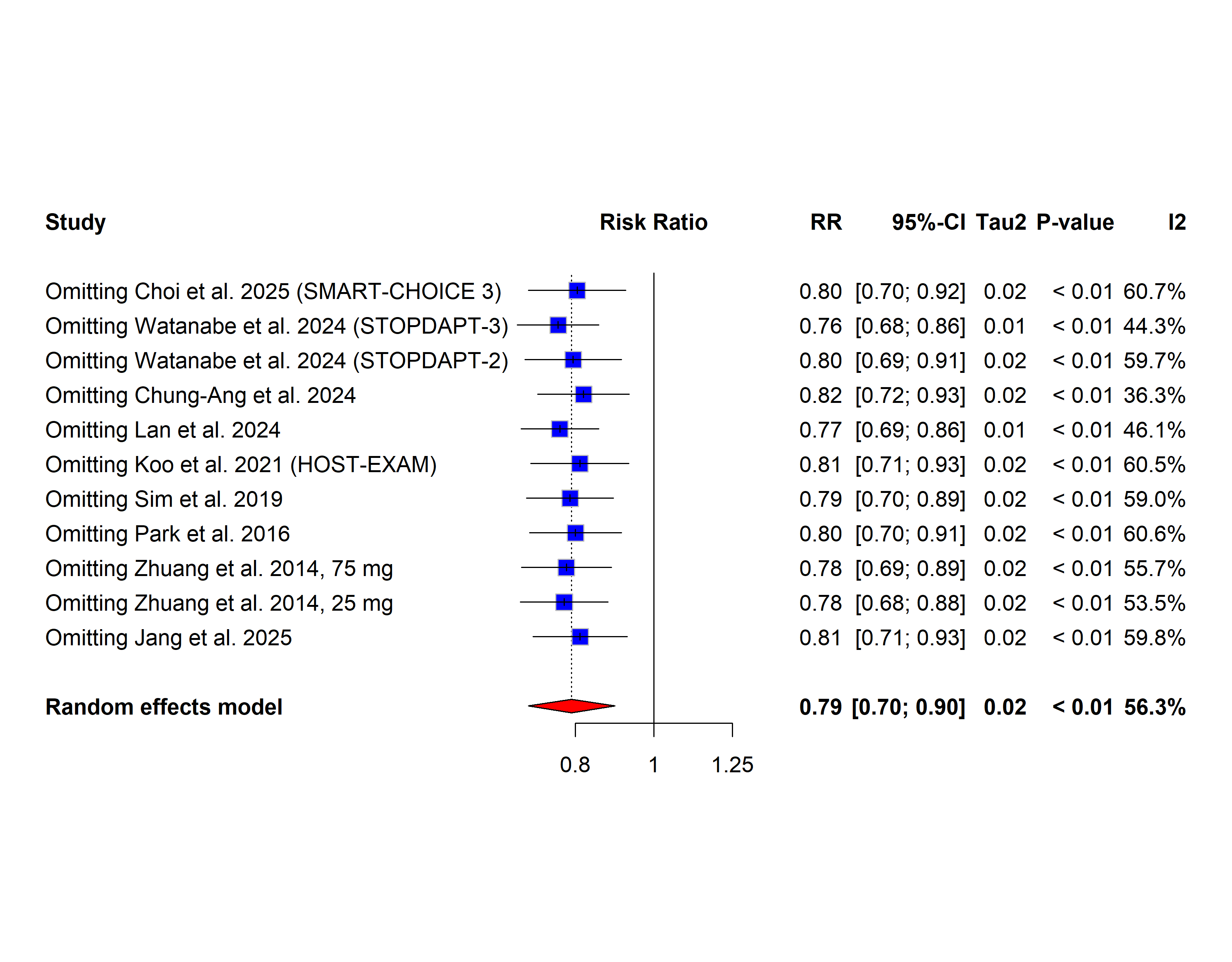


## Supplementary Figure 24: leave-one-out sensitivity analysis for major bleeding


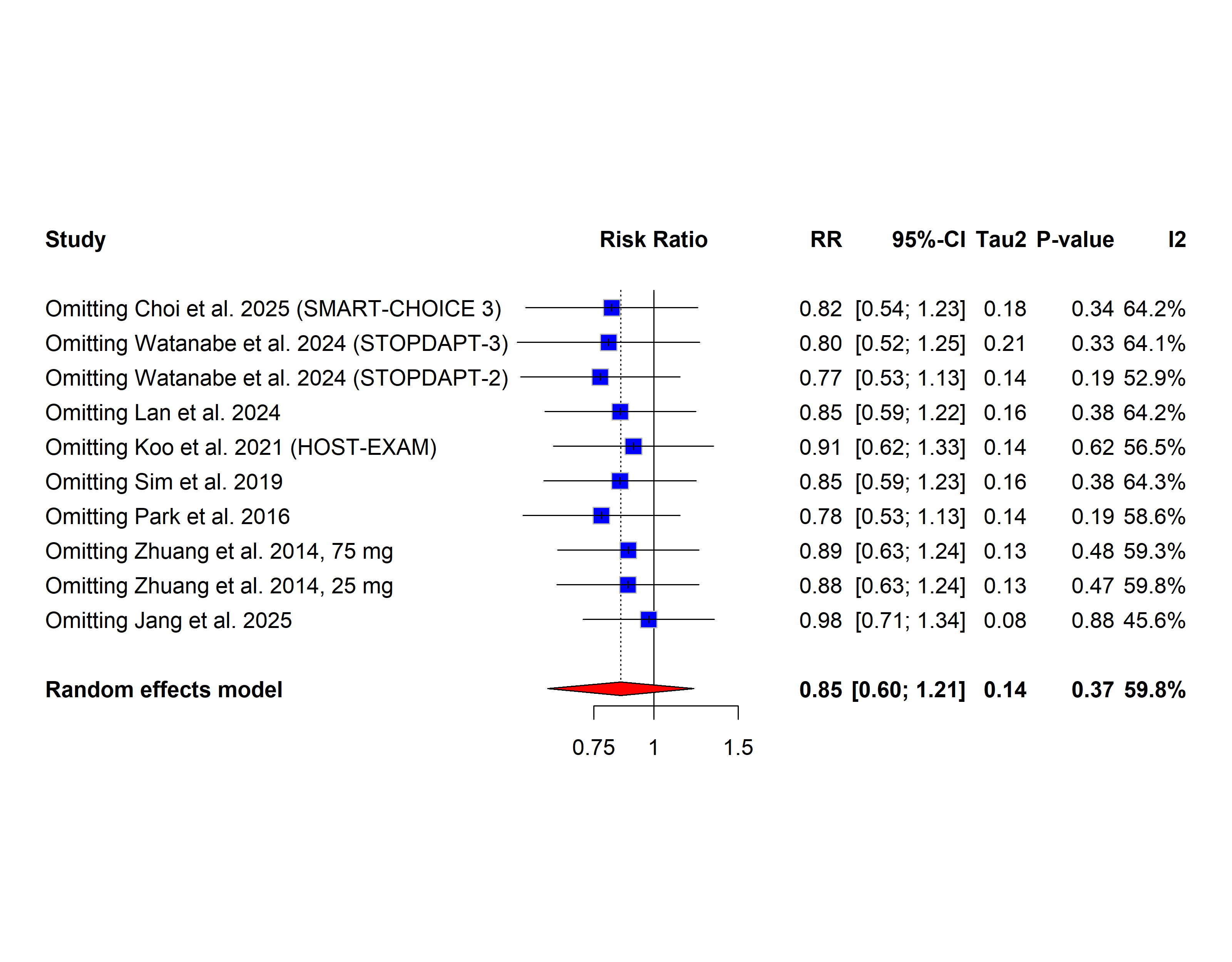


## Supplementary Figure 25: leave-one-out sensitivity analysis for all-cause mortality


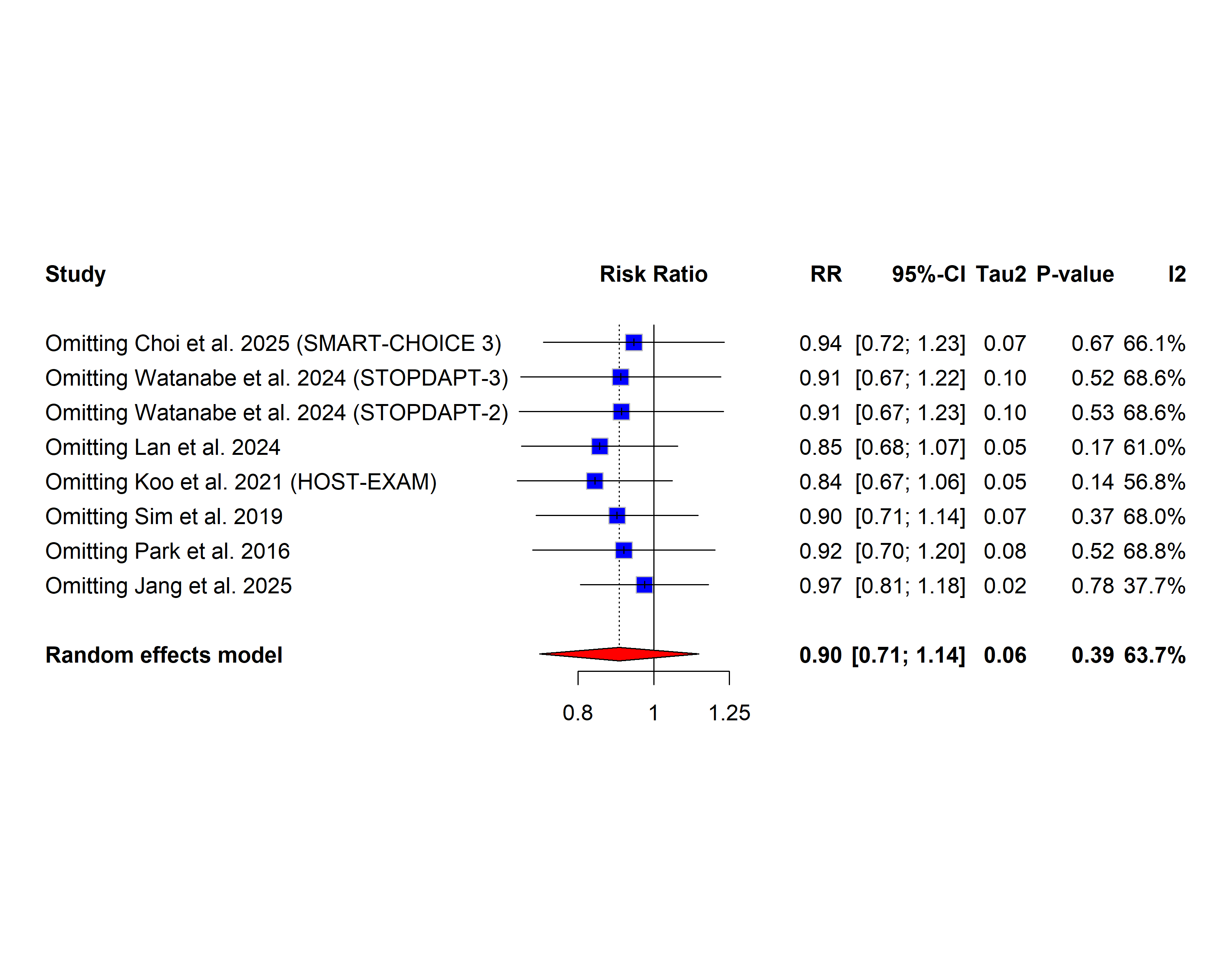


## Supplementary Figure 26: leave-one-out sensitivity analysis for cardiovascular mortality


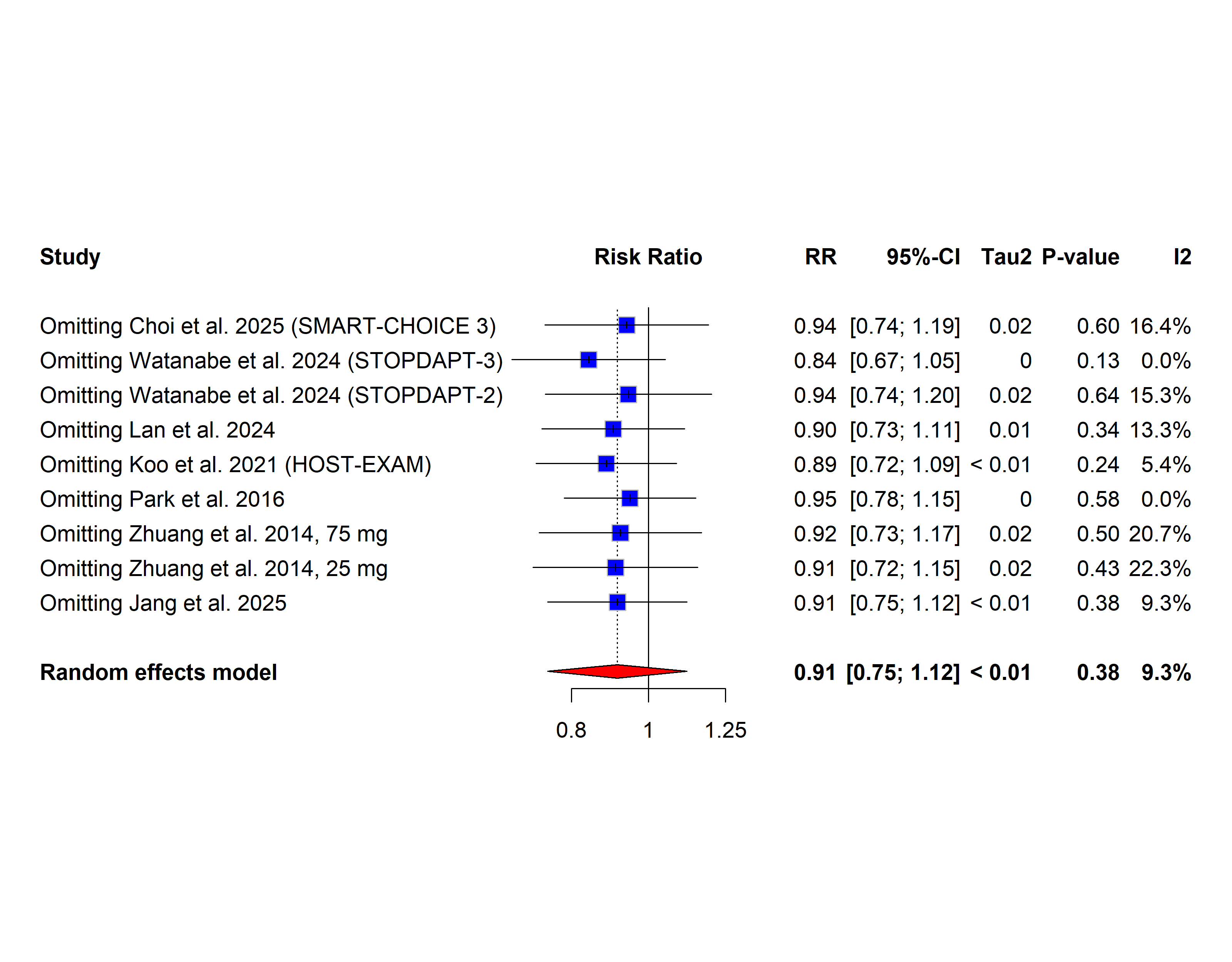


## Supplementary Figure 27: leave-one-out sensitivity analysis for stent thrombosis


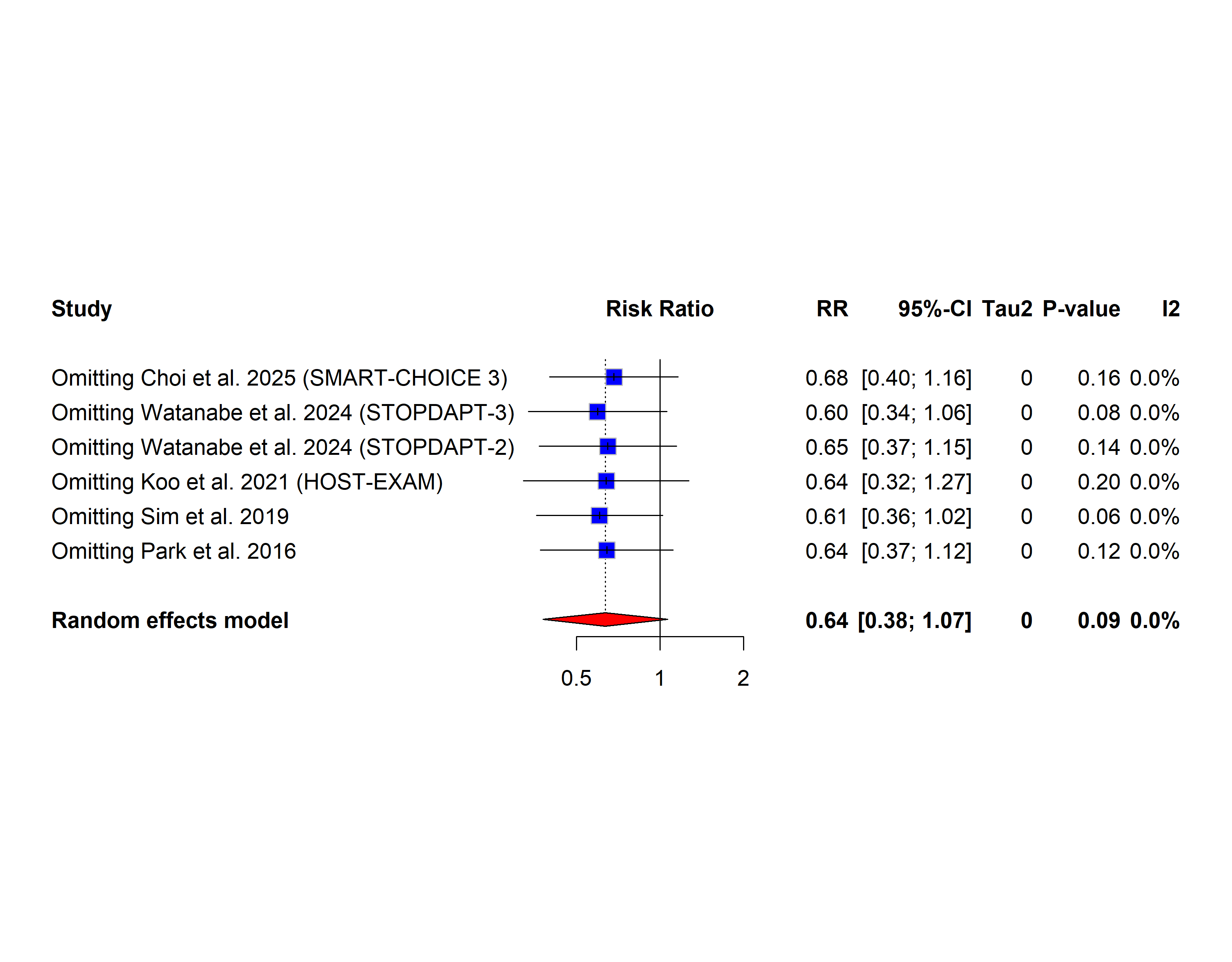


## Supplementary Figure 28: leave-one-out sensitivity analysis for any revascularization


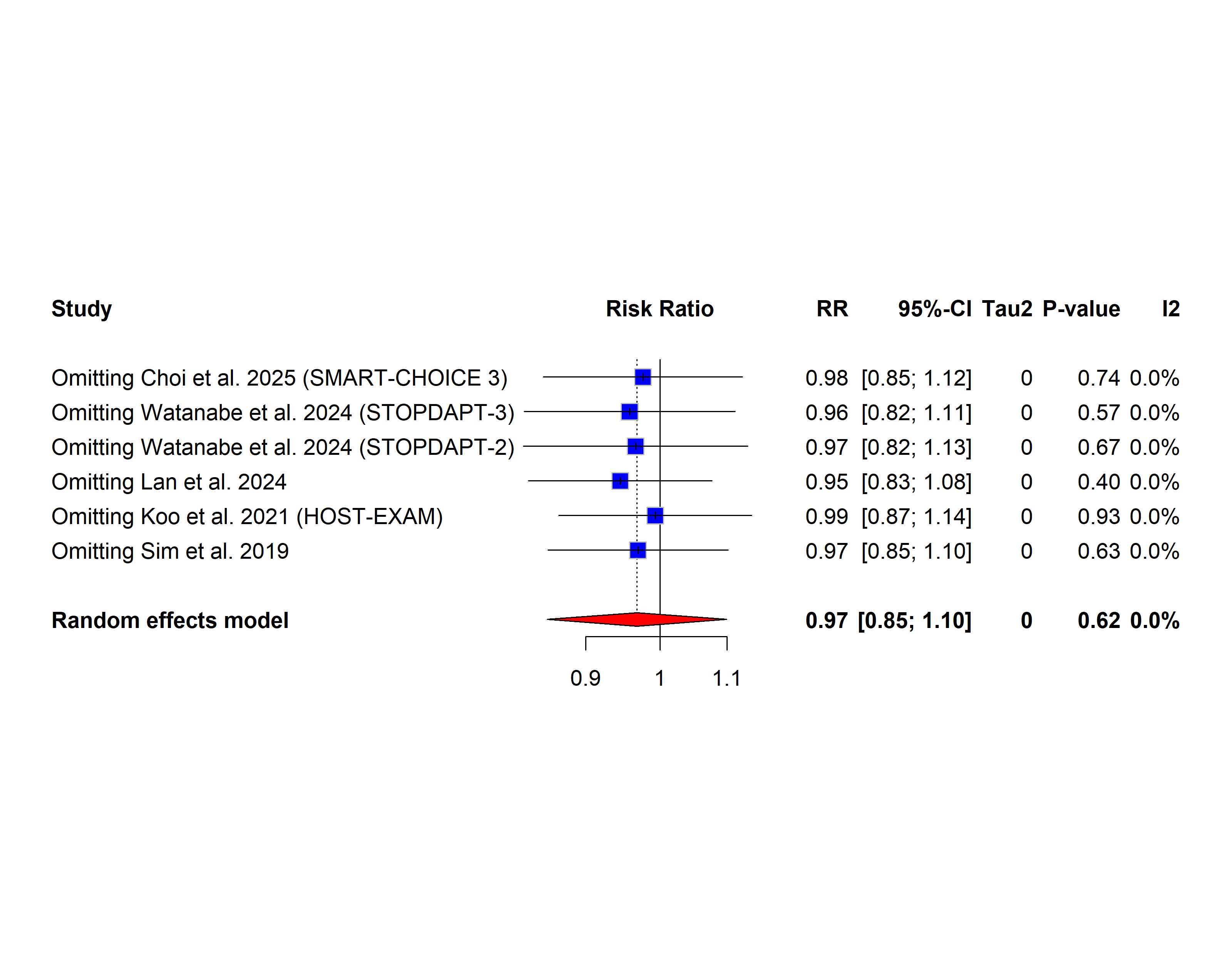


## Supplementary Figure 29: leave-one-out sensitivity analysis for TVR


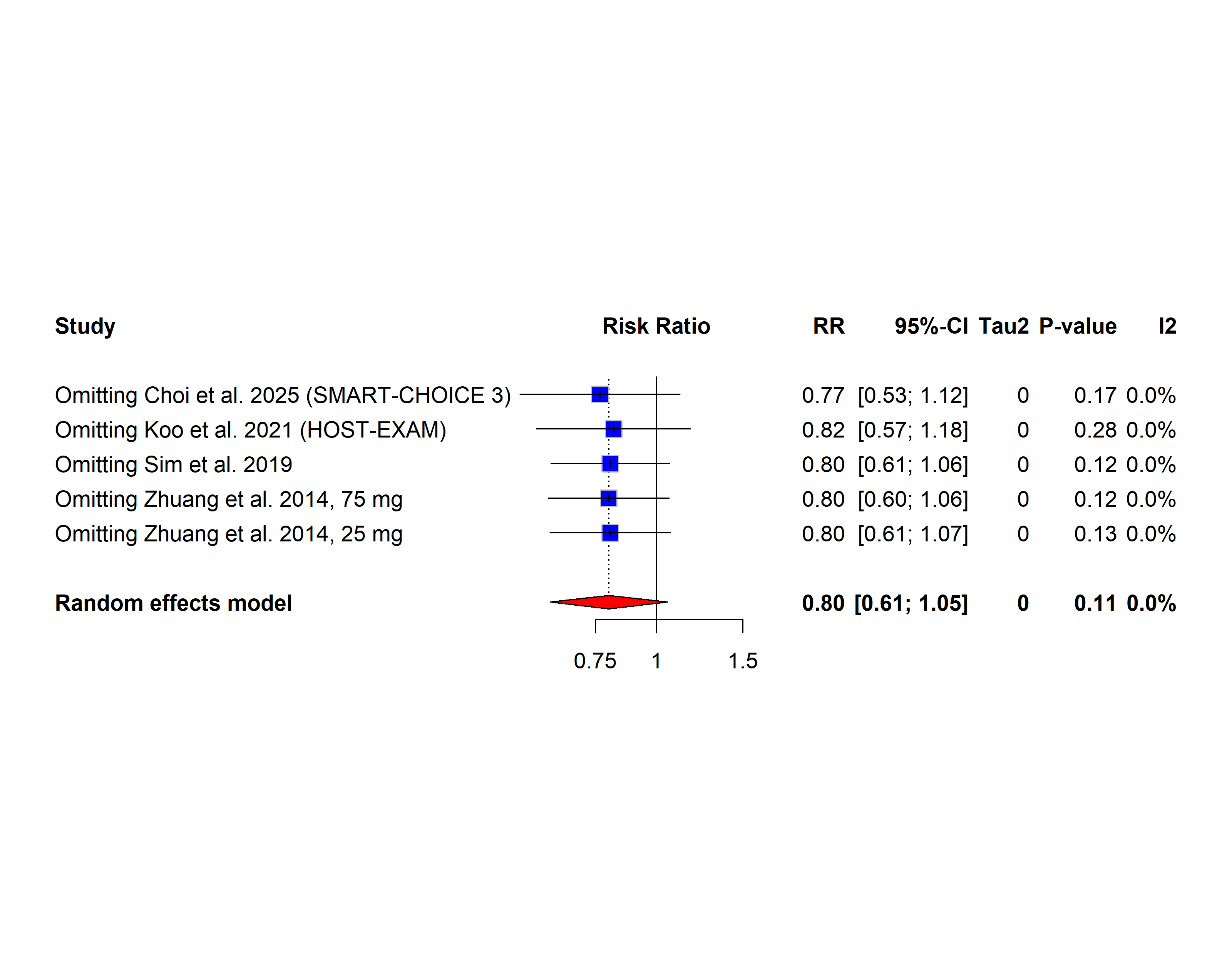


## Supplementary Figure 30: leave-one-out sensitivity analysis for TLR


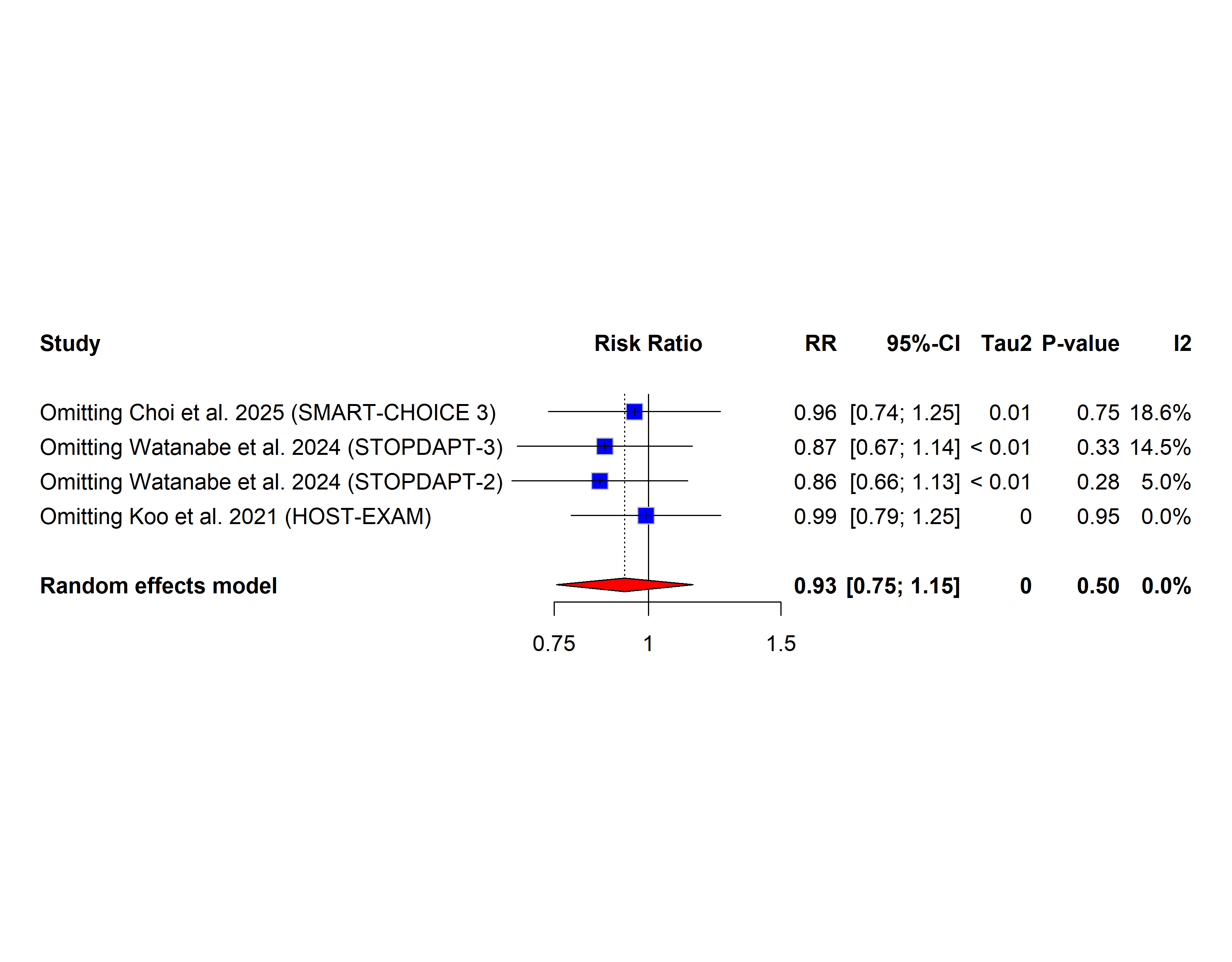


## Supplementary Figure 31: leave-one-out sensitivity analysis for haemorrhagic stroke


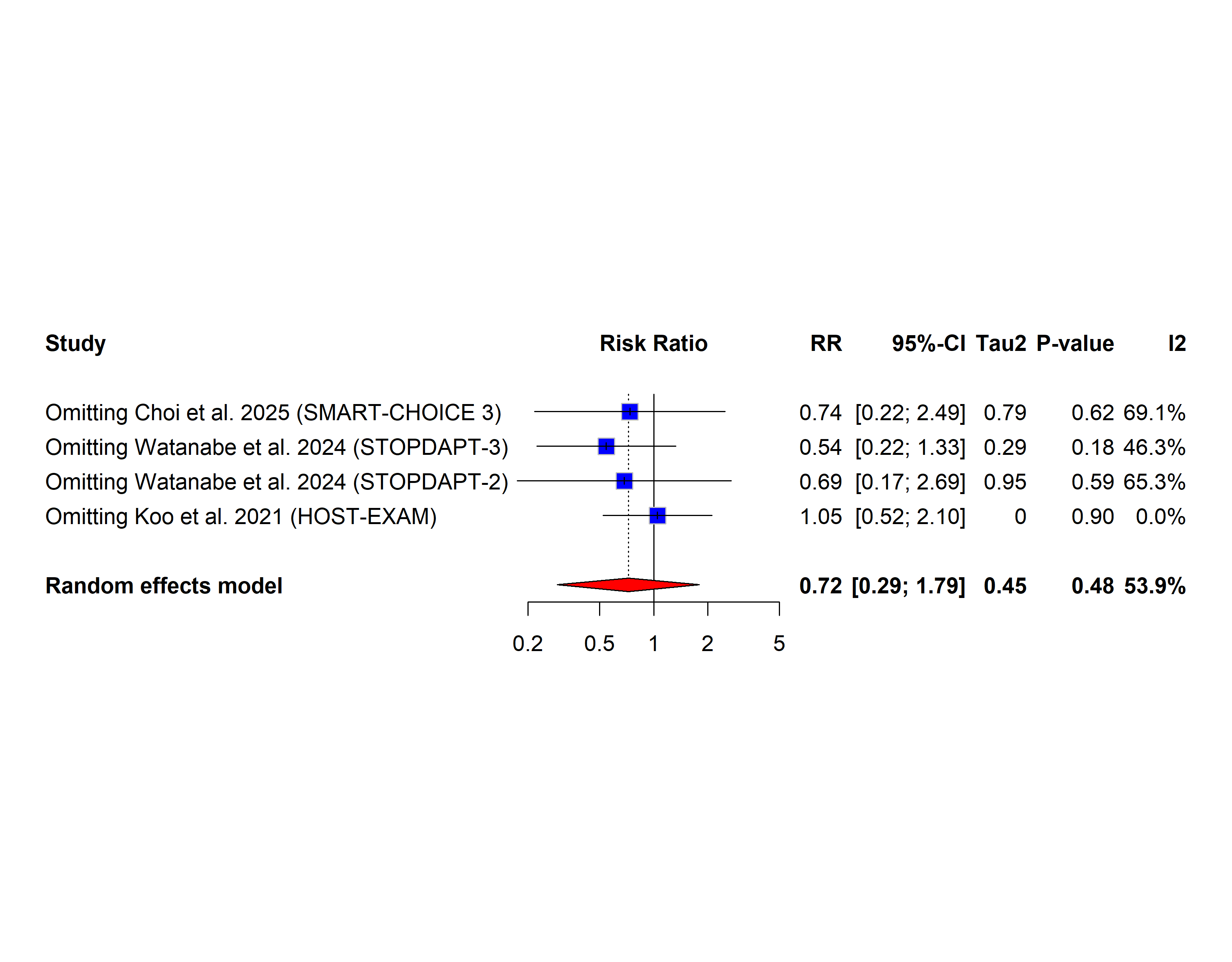


## Supplementary Figure 32: leave-one-out sensitivity analysis for gastrointestinal bleeding


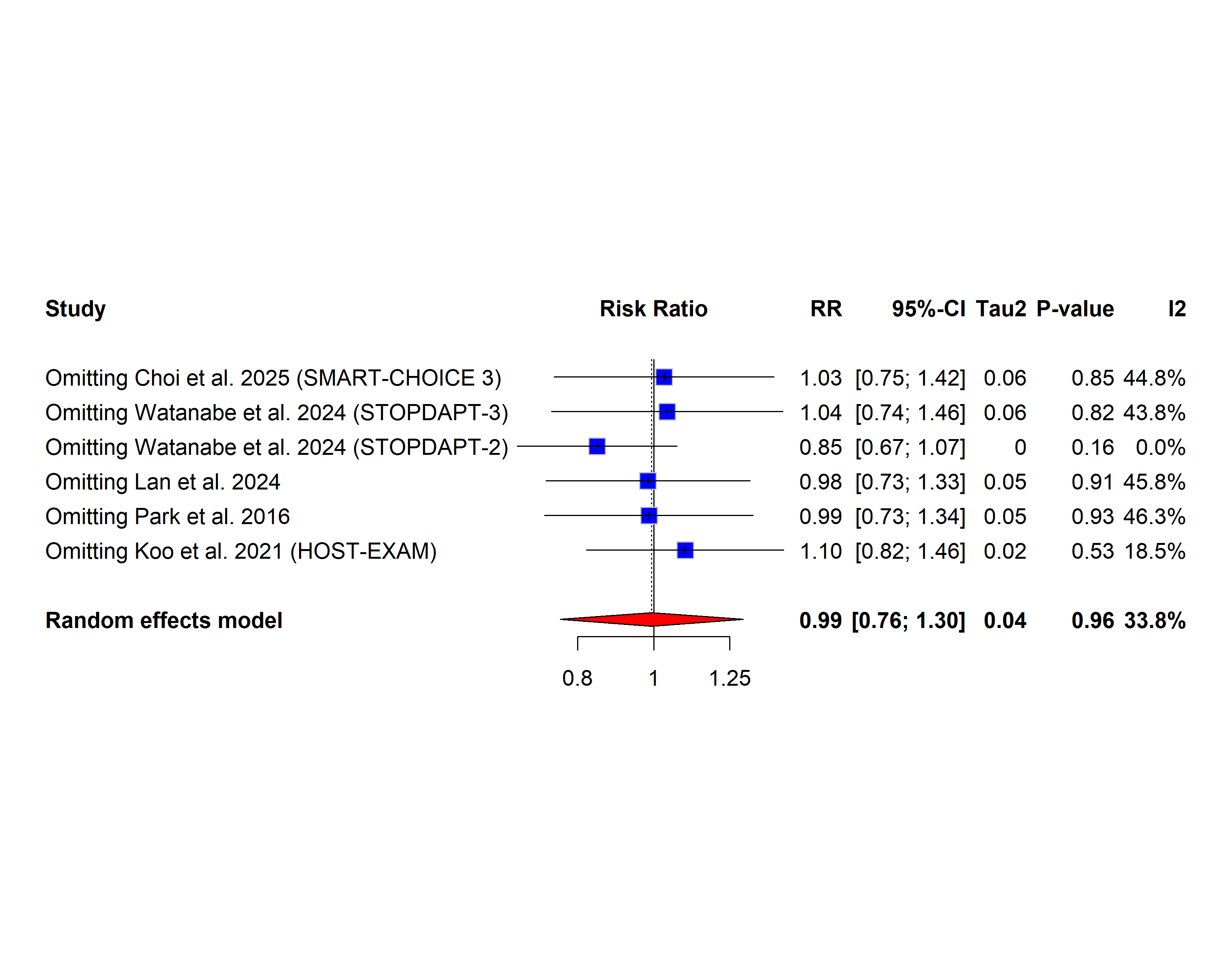


## Supplementary Figure 33: leave-one-out sensitivity analysis for intracranial bleeding


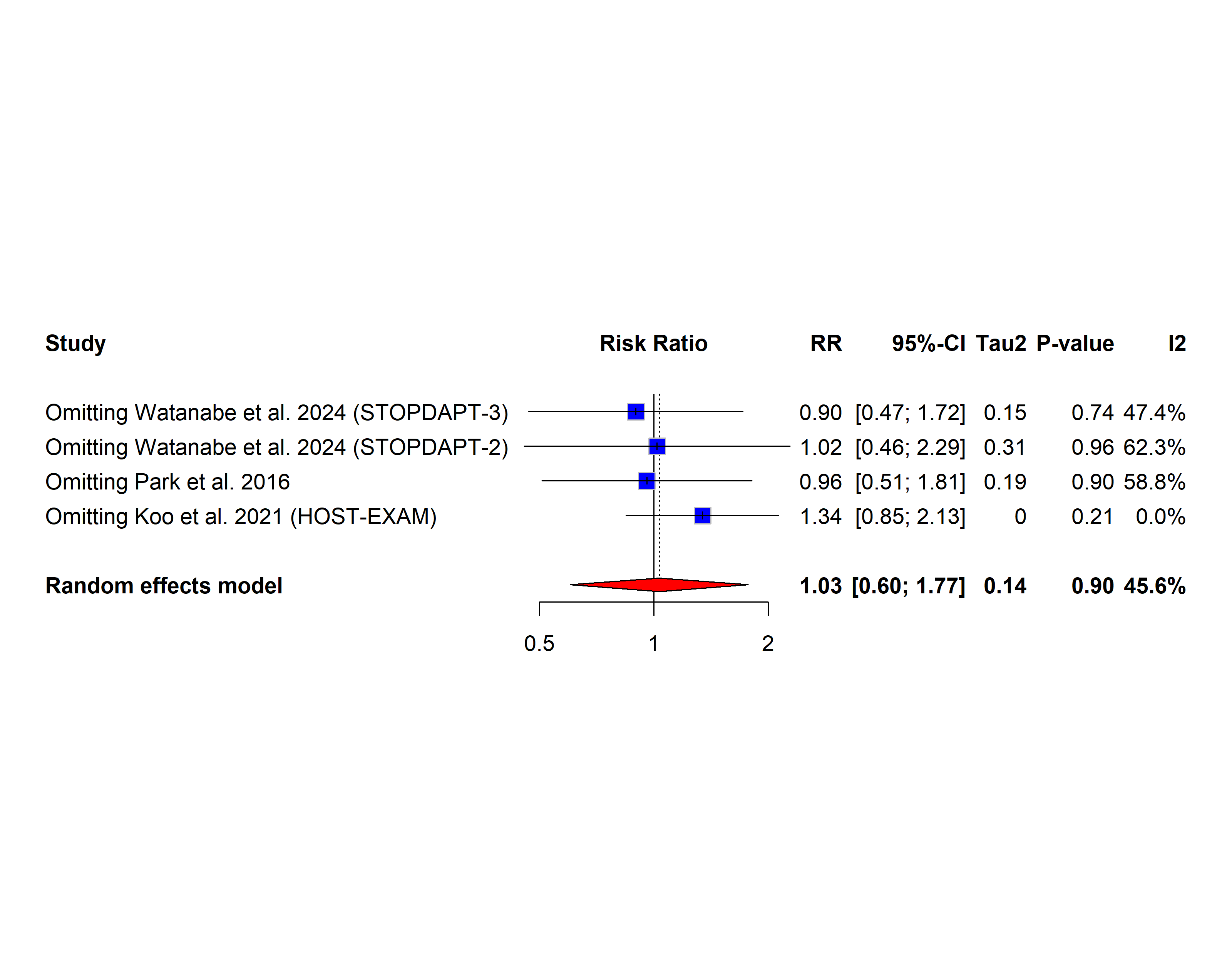


## Supplementary Figure 34: leave-one-out sensitivity analysis for MI


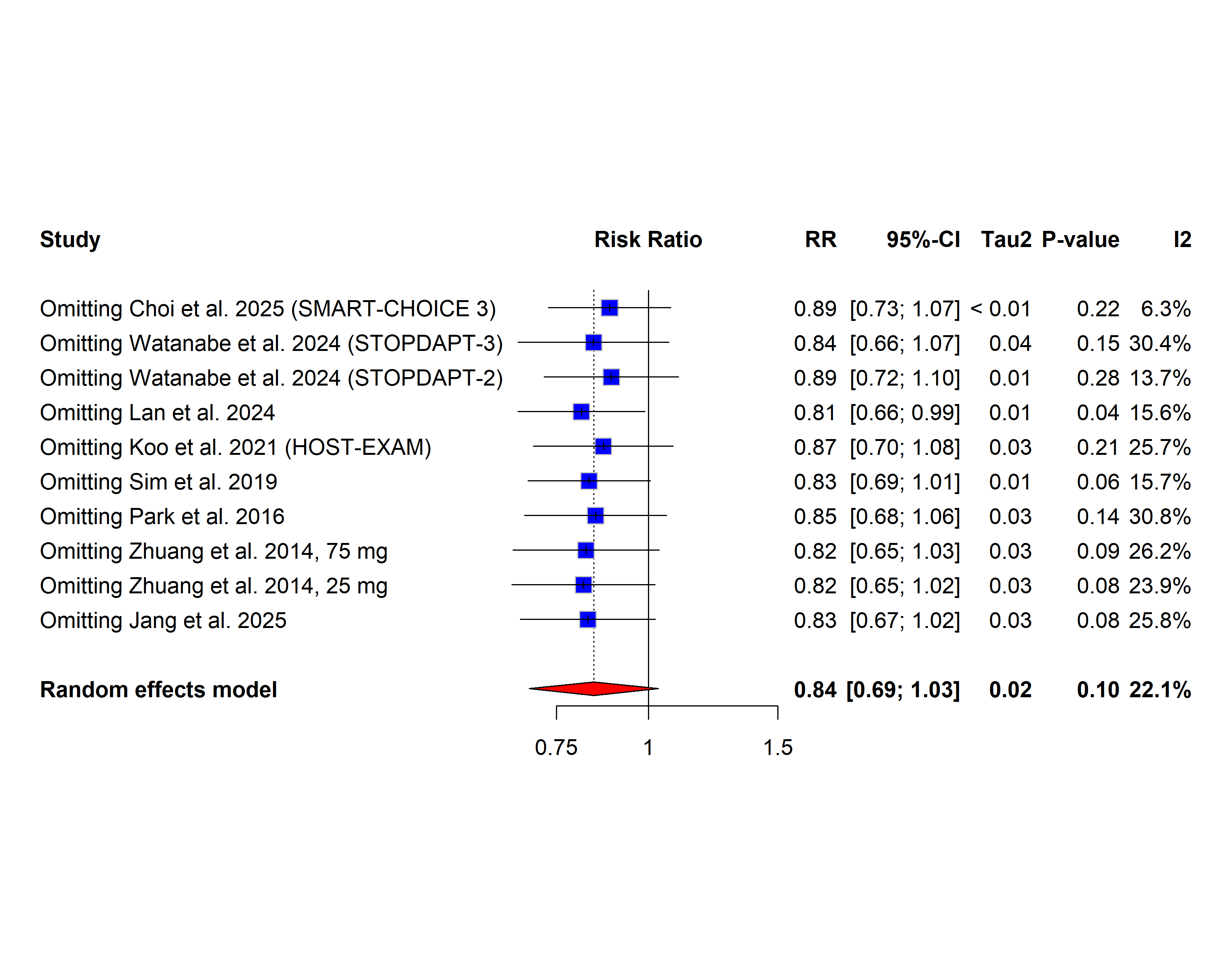


## Supplementary Figure 35: leave-one-out sensitivity analysis for stroke


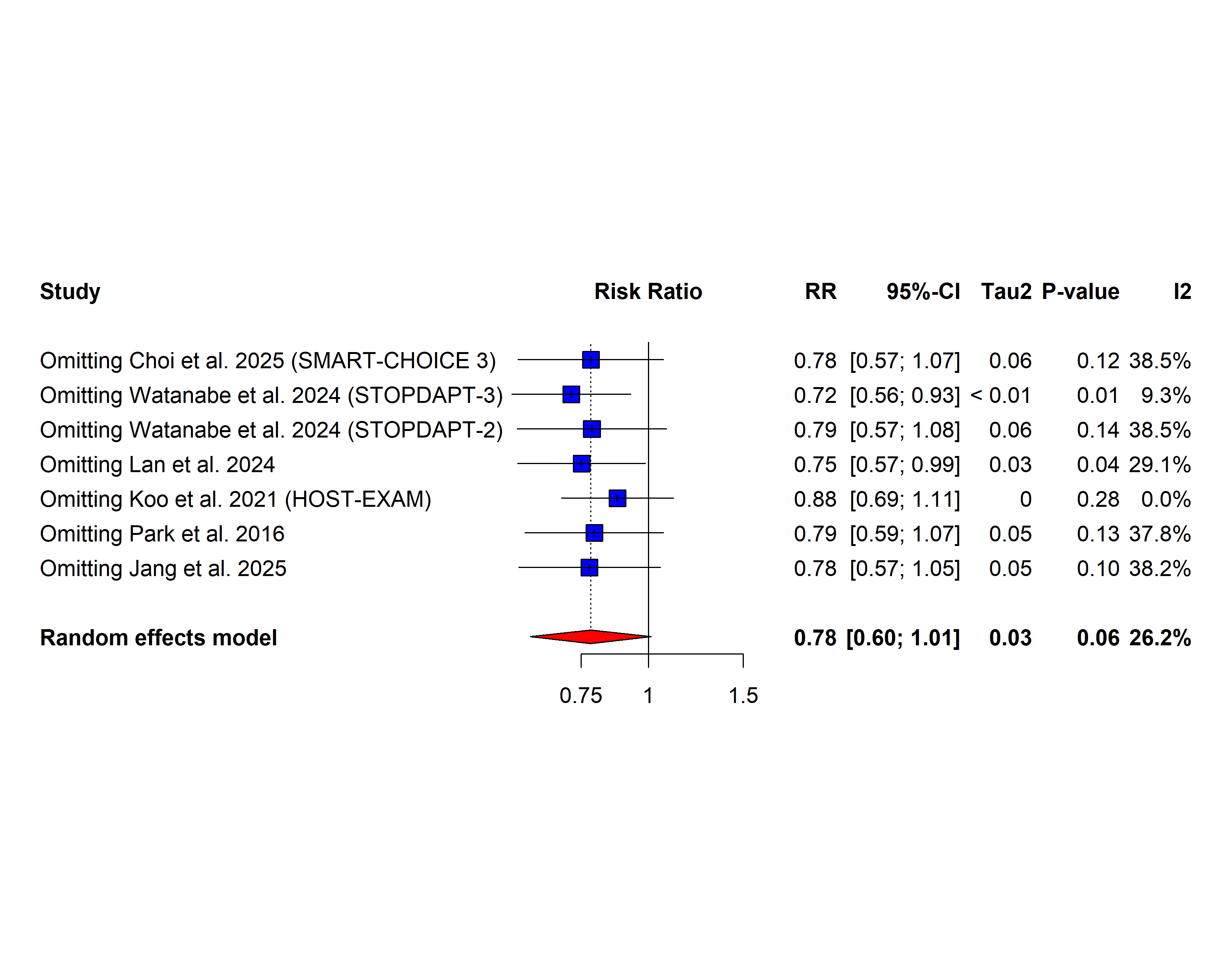


## Supplementary Figure 36: leave-one-out sensitivity analysis for Ischaemic stroke


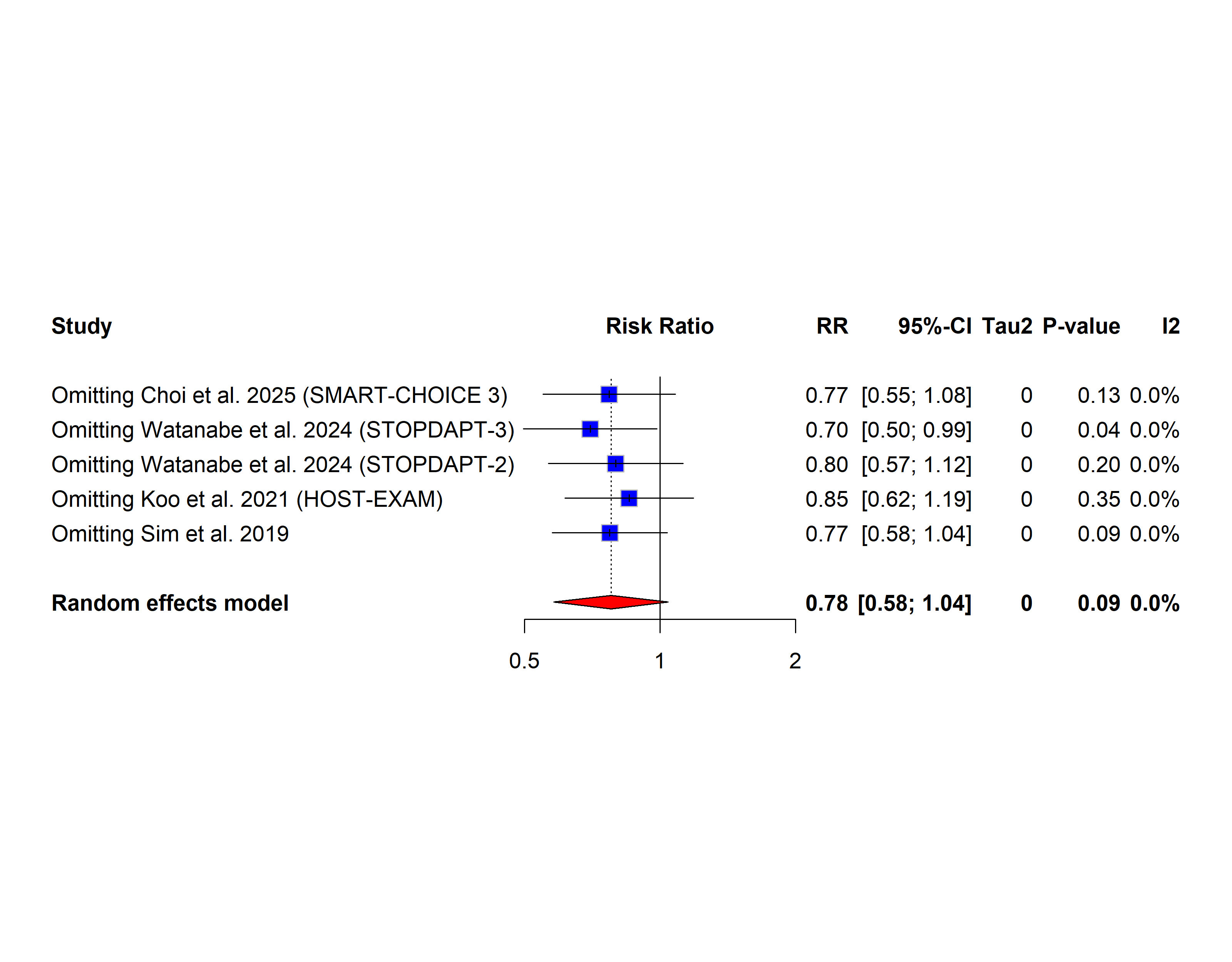


## Supplementary Figure 37: leave-one-out sensitivity analysis for all bleeding


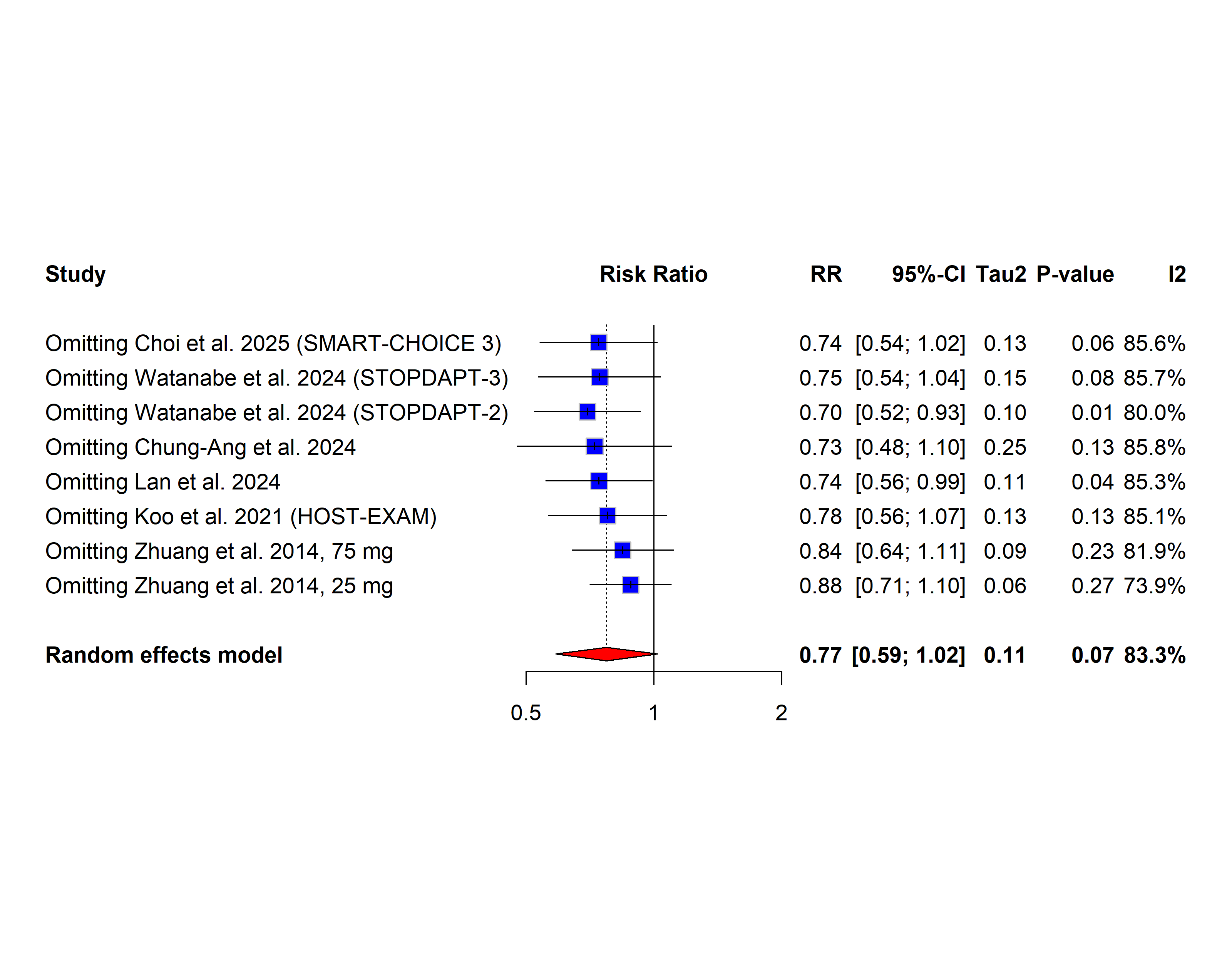


## Supplementary Figure 38: leave-one-out sensitivity analysis for NACE


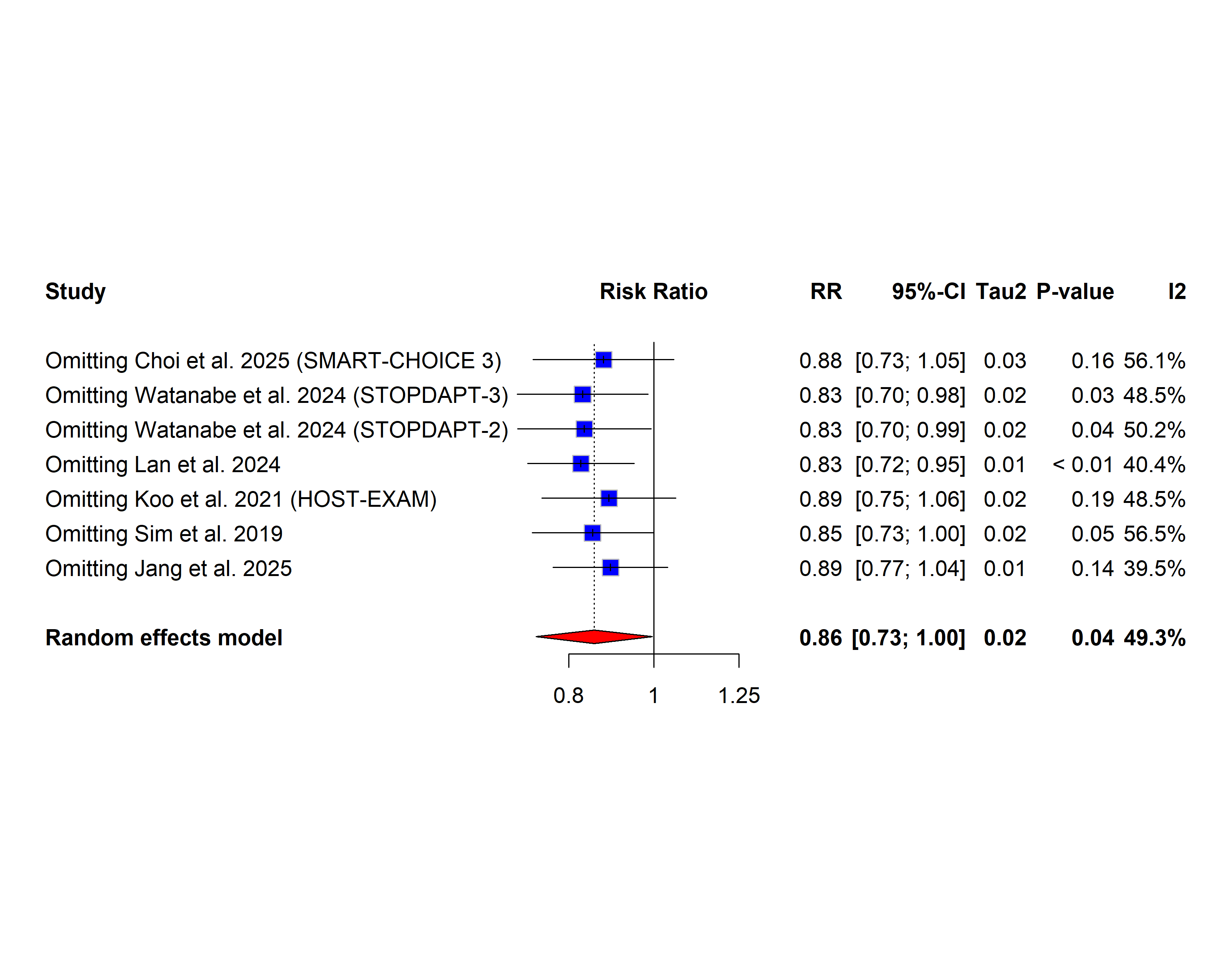


## **Supplementary Figure 39:** Bubble plot of meta-regression of significant covariate (Age) on MACE


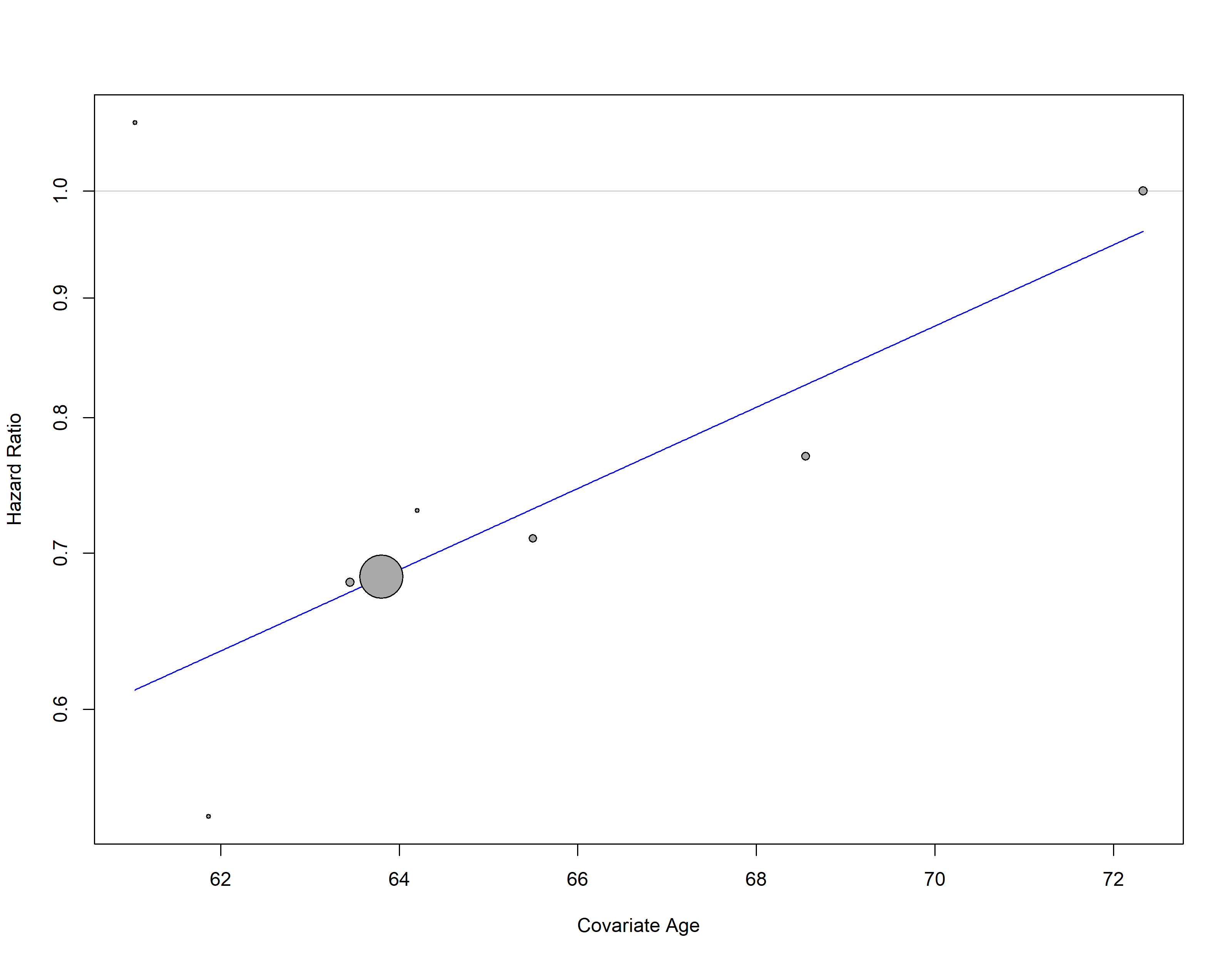


## **Supplementary Figure 40:** Bubble plot of meta-regression of significant covariate (Hypertension) on MACE


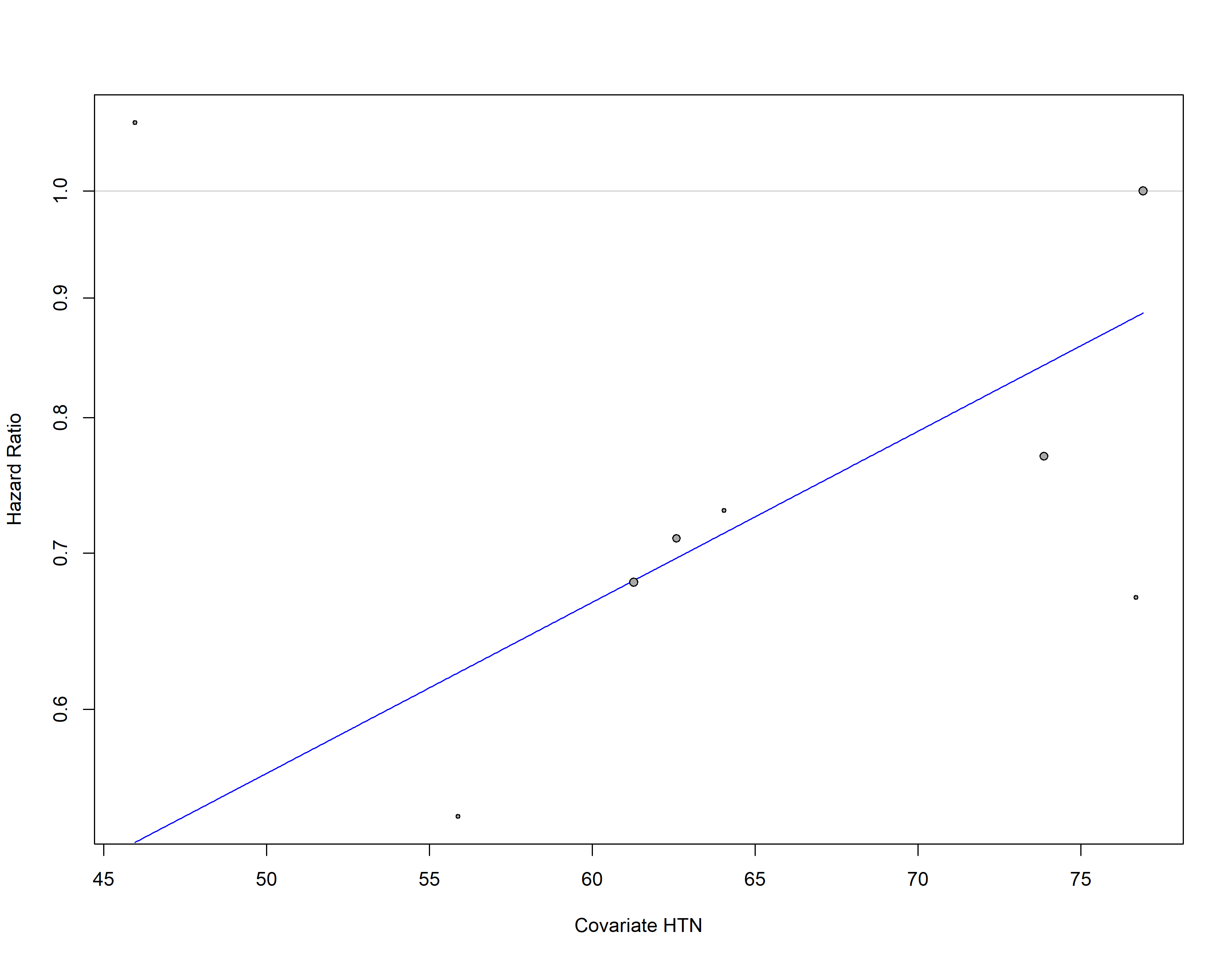


## **Supplementary Figure 41:** Bubble plot of meta-regression of significant covariate (LVEF) on MACE


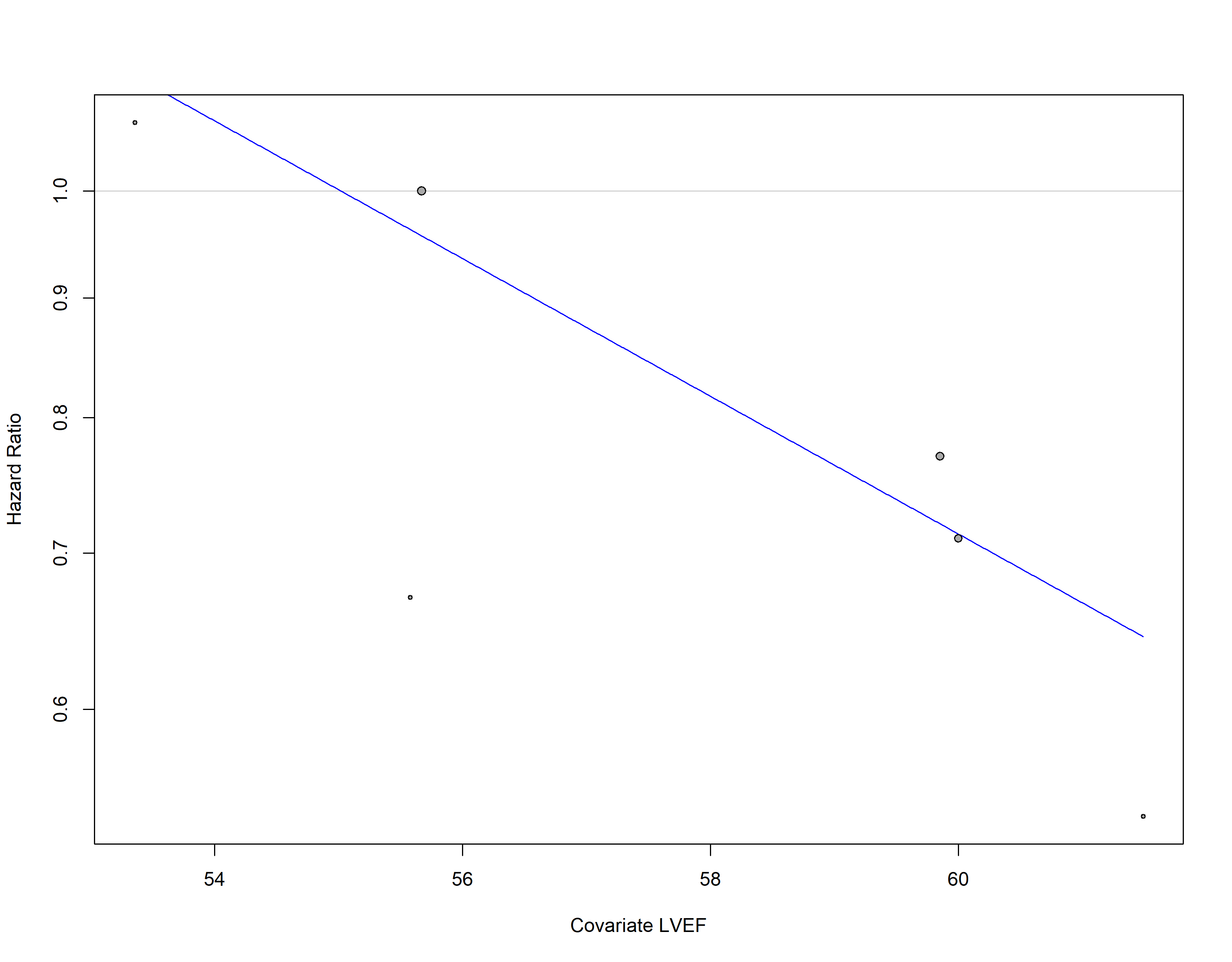


## Supplementary Table 1: Search strategy for each database

| Database | Search strategy | Search field | results |
| --- | --- | --- | --- |
| PubMed | (clopidogrel OR P2Y12 Inhibitor OR plavix) AND (aspirin OR Acetylsalicylic Acid) AND (Percutaneous coronary intervention OR PCI ) AND (post OR after OR Maintenance OR monotherapy OR long-term prevention OR Following OR chronic maintenance) | title, abstract only | 1566 |
| Scopus | (clopidogrel OR “P2Y12 Inhibitor” OR plavix) AND (aspirin OR “Acetylsalicylic Acid”) AND (“Percutaneous coronary intervention” OR PCI ) AND (post OR after OR Maintenance OR monotherapy OR “long-term prevention” OR Following OR “chronic maintenance”) | Article title, abstract, keywords | 10,293 |
| Web of Science | (clopidogrel OR P2Y12 Inhibitor OR plavix) AND (aspirin OR Acetylsalicylic Acid) AND (Percutaneous coronary intervention OR PCI ) AND (post OR after OR Maintenance OR monotherapy OR long-term prevention OR Following OR chronic maintenance) | Topic | 3089 |
| Cochren | (clopidogrel OR P2Y12 Inhibitor OR plavix) AND (aspirin OR Acetylsalicylic Acid) AND (Percutaneous coronary intervention OR PCI ) AND (post OR after OR Maintenance OR monotherapy OR long-term prevention OR Following OR chronic maintenance) | All Text | 1474 |
| Total |  |  | 16422 |

## Supplementary Table 2: Outcome definitions for each study

| **study ID** | **MACE** | **NACE** | **Major bleeding** | **Any bleeding** | **stent thrombosis** | **TVR** | **TLR** | **MI** |
| --- | --- | --- | --- | --- | --- | --- | --- | --- |
| Choi et al. 2025 (SMART-CHOICE 3) | a composite of death from any cause, MI, or stroke | MACE plus BARC type 3 or 5 bleeding | BARC type 3 or 5 bleeding | BARC type 2, 3, or 5 bleeding | according  to the Academic Research Consortium criteria | N.A | N.A | According to the Fourth  Universal Definition of Myocardial Infarction |
| Watanabe et al. 2024 (STOPDAPT-3) | a composite of death from cardiovascular cause, MI, definite stent thrombosis, or ischaemic stroke | A composite of cardiovascular death, MI, definite stent thrombosis, ischaemic stroke, or BARC 3 or 5 bleeding | BARC type 3 or 5, TIMI criteria (intracranial hemorrhage or ≥5 g/dL hemoglobin drop or ≥15% hematocrit drop), or GUSTO criteria (life-threatening bleeding, intracranial hemorrhage, or bleeding causing hypotension requiring intervention) | BARC type 2, 3, or 5 bleeding | Based on the ARC definition | PCI performed in the target vessel or revascularization by CABG, including TLR | PCI was performed in the target lesion (within 5 mm of the stent edges), or CABG performed for restenosis of the target lesion or for treatment of other complications | As classified by ARC However, the sensitivity of troponin is too high for the evaluation of peri-procedural MI, thus CKMB will be used in this situation |
| Watanabe et al. 2024 (STOPDAPT-2) | composite of cardiovascular death, MI, stroke, definite stent thrombosis. | a composite of cardiovascular death, MI, definite stent thrombosis, any stroke, or TIMI major/minor bleeding | When any of the following criteria is met: Intracranial hemorrhage, decrease in hemoglobin to ≥ 5 g/dL decrease in the hemoglobin concentration, and absolute drop in hematocrit to ≥ 15% (Baseline – Onset of the event), and using BARC type 3 or 5 | According to TIMI and BARC bleeding | Based on the ARC definition | PCI performed in the target vessel or revascularization by CABG, including TL | PCI performed in the target lesion (within 5 mm of the stent edges), or CABG performed for restenosis of the target lesion or for treatment of other complications | As classified by ARC: However, the sensitivity is too high for the evaluation with Troponin of the peri-procedural MI, thus CKMB will be used |
| Chung-Ang et al. 2024 | composite of cardiovascular death, MI or stroke. | N.A | N.A | N.A | N.A | N.A | N.A | N.A |
| Lan et al. 2024 | cardiac death, ischemic stroke, or MI. | the composite cardiac death, ischemic stroke, MI, and bleeding BARC type 3 or greater. | BARC: type 3 bleeding or greater. | BARC type ≥ 2 bleeding | N.A | N.A | N.A | Myocardial infarction (MI) was characterized by raised cardiac biomarkers (troponin or the myocardial seg-  ment of creatine kinase exceeding the 99th percentile  of the upper limit of normal) accompanied by ischemic  symptoms or electrocardiogram results suggesting ischemia. |
| Koo et al. 2021, Kang et al. 2024(HOST-EXAM) | cardiac death, non-fatal MI, ischaemic stroke, readmission due to acute coronary syndrome, and definite or probable stent thrombosis | a composite of all-cause death, non-fatal MI, stroke, readmission due to acute coronary syndrome, and major bleeding complications | BARC: type bleeding of at least 3 | BARC type ≥2 bleeding | defined as abrupt onset of an acute coronary syndrome, which is confirmed with angiographic evidence of acute thrombotic occlusion in the stented segment of a coronary artery. | any repeat revascularization procedure (PCI or CABG) involving at least one of the target vessels that were treated in the index procedure | any repeat revascularization procedure (PCI or CABG) at the original lesion of the index procedure any time during the follow-up period | myocardial infarction is diagnosed by detection of an increased level of cardiac biomarkers, preferably cardiac troponins, with at least one value above the 99th percentile of the upper reference limit, accompanied with at least one of the followings: symptoms of myocardial ischemia, ECG changes (ST elevation, left bundle branch block, ST change without ST elevation), and imaging findings suggestive of myocardial infarction (loss of viable myocardium or new regional wall motion abnormality. |
| Sim et al. 2019 | defined as a composite of death from any cause, MI, repeat PCI, stent thrombosis, or ischemic stroke. | composite of death from any cause, MI, repeat PCI, stent thrombosis, ischemic stroke, or TIMI major bleeding | According to TIMI major bleeding | N.A | N.A | a repeat PCI of any segment within the entire major coronary vessel proximal and distal to a target lesion, including the target lesion itself. | N.A | N.A |
| Park et al. 2016 | a composite of cardiac death, MI, or stroke. | N.A | According to the BARC (type 3 or 5 bleeding), or TIMI major bleeding | N.A | N.A | N.A | N.A | N.A |
| Zhuang et al. 2014 | a combined incidence of cardiac death, myocardial infarction, urgent target vessel revascularization | N.A | Major bleeding was defined as intracranial, intraocular, or retroperitoneal hemorrhage, clinically overt blood loss resulting in a decrease in hemoglobin of more than 3 g per deciliter, any decrease in hemoglobin of more than 4 g per deciliter, or transfusion of 2 or more units of packed red blood cells or whole blood | BARC type 2, 3, or 5 bleeding | N.A | N.A | Revascularization was considered clinically driven if prompted  by symptoms or signs consistent with myocardial ischemia or  if lesion diameter stenosis was more than 70% at follow-up. | The definition of myocardial  infarction was development of pathologic Q waves (≥30 ms in  duration and ≥0.1 mV in depth) in ≥2 contiguous precordial  leads or ≥2 adjacent limb leads, or elevation of creatine kinase  isoenzyme MB (CK-MB) ≥2 times the upper limit of normal. |
| Jang et al. 2025 | all-cause death, MI, stroke | all-cause death, MI, stroke, and major bleeding | According to BARC: types 3–5, including overt bleeding with a haemoglobin decrease of at least 3 g/dL, intracranial bleeding, coronary artery bypass grafting (CABG)–related bleeding, and probable or definite fatal bleeding. | BARC type 2, 3, or 5 bleeding | N.A | N.A | N.A | MI after discharge was defined as the presence  of clinical symptoms, MI-associated electrocardiographic  changes, or abnormal imaging findings combined with crea-  tine kinase–myocardial band increment above the upper normal limit or troponin T/I greater than the 99th percentile  of the upper limit of normal, unrelated to an interventional  procedure. |

ACS: acute coronary syndrome, ARC: Academic Research Consortium, BARC: Bleeding Academic Research Consortium, DAPT: Dual Antiplatelet Therapy, HBR: High Bleeding Risk, MACE: Major Adverse Cardiovascular Events, MI: myocardial infarction, N.A.: Not Available, NACE: Net Adverse Clinical Events, PCI: Percutaneous Coronary Intervention, TIMI: Thrombolysis in Myocardial Infarction, TLR: Target Lesion Revascularization, TVR: Target Vessel Revascularization.

## Supplementary Table 3A: Baseline patient characteristics of included studies

| **Study ID** | **groups** | **No. of participants** | **Age (years), mean (SD)** | **Sex (males) N (%)** | **BMI, mean (SD)** | **DM, N (%)** | **Unstable angina, N (%)** | **NSTEMI, N (%)** | **STEMI, N (%)** | **HTN, N (%)** | **Dyslipidemia N (%)** | **Current Smoking, N (%)** | **CKD, N (%)** | **HF, N (%)** | **LVEF, mean (SD)** | **Previous PCI, N (%)** | **Previous CABG, N (%)** | **Stroke, N (%)** |
| --- | --- | --- | --- | --- | --- | --- | --- | --- | --- | --- | --- | --- | --- | --- | --- | --- | --- | --- |
| Choi et al. 2025 (SMART-CHOICE 3) | Clopidogrel | 2752 | 65.67 (11.13) | 2240 (81.4%) | 24.97(2.97) | 1119 (40.7%) | 797 (29%) | 678 (24.6%) | 605 (22%) | 1756 (63.8%) | 1626 (59.1%) | 448 (16.3%) | 242 (8.8%) | N.A | 60 (7.42) | N.A | N.A | 76 (2.8%) |
|  | Aspirin | 2754 | 65.33(11.13) | 2264(82.2%) | 24.93(2.82) | 1128 (41%) | 823 (29.9%) | 652 (23.7%) | 617 (22.4%) | 1690(61.4%) | 1604 (58.2%) | 488 (17.7%) | 260 (9.4%) | N.A | 60 (7.42) | N.A | N.A | 66 (2.4%) |
| Watanabe et al. 2024 (STOPDAPT-3) | Clopidogrel | 2913 | 72.33 (11.87) | 2242 (77%) | 23.63 (3.26) | 1306 (44.8%) | 411 (14.1%) | 561 (19.3%) | 1204 (41.3%) | 2271 (78%) | 1963 (67.4%) | 682 (23.4%) | 1530 (52.5%) | 643 (22.1%) | 55.67 (10.38) | 459 (15.8%) | 56 (1.9%) | 272 (9.3%) |
|  | Aspirin | 2920 | 72.33 (11.87) | 2228 (76.3%) | 23.77 (3.41) | 1319 (45.2%) | 411 (14.1%) | 513 (17.6%) | 1253 (42.9%) | 2215 (75.9%) | 1974 (67.6%) | 700 (24%) | 1488 (51%) | 658 (22.5%) | 55.67 (11.13) | 453 (15.5%) | 66 (2.3%) | 266 (9.1%) |
| Watanabe et al. 2024 (STOPDAPT-2) | Clopidogrel | 1471 | 68.1 (10.9) | 1159 (78.8%) | 24.4 (3.5) | 571 (38.8%) | 185 (12.6%) | 85 (5.8%) | 287 (19.5%) | 1084 (73.7%) | 1096 (74.5%) | 387 (26.3%) | 77 (5.2%) | 106 (7.2%) | 59.9 (10.1) | 360 (24.5%) | 16 (1.1%) | 77 (5.2%) |
|  | Aspirin | 1486 | 69 (10.4) | 1138 (76.6%) | 24.3 (3.5) | 567 (38.2%) | 208 (14%) | 100 (6.7%) | 265 (17.8%) | 1100 (74%) | 1115 (75%) | 308 (20.7%) | 82 (5.5%) | 101 (6.8%) | 59.8 (10.5) | 371 (25%) | 42 (2.8%) | 104 (7.0) |
| Chung-Ang et al. 2024 | Clopidogrel | 67653 | 63.8 (16.3) | N.A | N.A | N.A | N.A | N.A | N.A | N.A | N.A | N.A | N.A | N.A | N.A | N.A | N.A | N.A |
|  | Aspirin | 65802 |  | N.A | N.A | N.A | N.A | N.A | N.A | N.A | N.A | N.A | N.A | N.A | N.A | N.A | N.A | N.A |
| Lan et al. 2024 | Clopidogrel | 422 | 66.38 (11.67) | 321 (76.1%) | 24.8 (3.77) | 129 (30.6%) | 122 (28.9%) | 75 (17.8%) | 158 (37.4%) | 308 (73%) | 88 (20.9%) | 230 (54.5%) | 21 (5%) | N.A | N.A | 84 (20%) | N.A | N.A |
|  | Aspirin | 582 | 62.62(12.44) | 453(77.8%) | 24.96(3.3) | 174(29.9%) | 148(25.4%) | 124(21.3%) | 235(40.4%) | 335(61%) | 107(18.4%) | 339(58.2%) | 18(3.1%) | N.A | N.A | 94 (16.2%) | N.A | N.A |
| Koo et al. 2021 (HOST-EXAM) | Clopidogrel | 2710 | 63·5 (10·7) | 2015 (74·4%) | N.A | 925 (34·1%) | 975 (36·0%) | 526 (19·4%) | 463 (17·1%) | 1664 (61.4%) | 1884 (69.5%) | 545 (20.1%) | 356 (13.1%) | N.A | N.A | N.A | N.A | 120 (4·4%) |
|  | Aspirin | 2738 | 63·4 (10·7) | 2039 (74·7%) | N.A | 935 (34·3%) | 959 (35·2%) | 528 (19·4%) | 470 (17·2%) | 1674 (61.4%) | 1883 (69·0%) | 581 (21·3%) | 337 (12·4%) | N.A | N.A | N.A | N.A | 133 (4·9%) |
| Sim et al. 2019 | Clopidogrel | 533 | 60.9(11.7) | 418 (78.5) | 24.1(3.3) | 110 (20.7) | N.A | N.A | 273 (51.3) | 244 (45.7) | 70 (13.2) | 336 (63.0) | N.A | 10 (0.8) | 53.5 (9.9) | N.A | N.A | 20(3.6) |
|  | Aspirin | 1286 | 61.1(11.7) | 1,006 (78.2) | 24.1(3) | 275 (21.4) | N.A | N.A | 659 (51.2) | 592 (46.0) | 171 (13.3) | 809 (62.9) | N.A | 5 (0.9) | 53.3 (9.5) | N.A | N.A | 50(3.8) |
| Park et al. 2016 | Clopidogrel | 771 | 63.67(11.14) | 570 (73.9) |  | 325 (42.2) | N.A | 241 (31.3) | 83 (10.8) | 497 (64.5) | 258 (33.5) | 174 (22.6) | 79 (10.2) | N.A | 62 (8.9) | 109 (14.1) | 26 (3.4) | 47 (6.1) |
|  | Aspirin | 2472 | 61.3 (11.87) | 1811 (73.3) |  | 834 (33.7) | N.A | 654 (26.5) | 363 (14.7) | 1315 (53.2) | 705 (28.5) | 429 (17.4) | 199 (8.1) |  | 61.33(8.9) | 243 (9.8) | 63 (2.5) | 79 (3.2) |
| Zhuang et al. 2014 | Clopidogrel (75 mg/d) | 248 | 70.1 (6.2) |  | 26.6 (4.7) | N.A | N.A | N.A | N.A | 168 (67.7) | N.A | N.A | 59 (23.8) |  | 61(10) | N.A | N.A | 59 (23.8) |
|  | Clopidogrel (25 mg/d) | 224 | 69.2 (5.7) |  | 25.1 (3.2) | N.A | N.A | N.A | N.A | 149 (66.5) | N.A | N.A | 33 (14.7) |  | 65 (10) | N.A | N.A | 51 (22.8) |
|  | Aspirin | 283 | 68.3 (7.8) |  | 24.9 (4.1) | N.A | N.A | N.A | N.A | 192 (67.8) | N.A | N.A | 42 (14.8) |  | 64(12) | N.A | N.A | 73 (25.8) |
| Jang et al. 2025 | Clopidogrel | 916 | N.A | 514 (56.1) | 302 (32.9) | 538 (58.7) | N.A | N.A | N.A | 707 (77.2) | 563 (61.4) | 188 (20.6) | 609 (66.5) | 115 (12.5) | 55.1 (12.7) | 203 (22.2) | 35 (3.8) | 193 (21.1) |
|  | Aspirin | 838 | N.A | 485 (57.8) | 302 (36.1) | 480 (57.3) | N.A | N.A | N.A | 638 (76.1) | 542 (64.6) | 162 (19.3) | 566 (67.5) | 89 (10.6) | 56.1 (12) | 186 (22.2) | 29 (3.4) | 141 (16.9) |

CKD: Chronic Kidney Disease, DM: Diabetes, HF: Heart failure, HTN: Hypertension, N: number, N.A: Not Available, NSTEMI: Non-ST-Elevation Myocardial Infarction, STEMI: ST-Elevation Myocardial Infarction.

## Supplementary Table 3B: Baseline patient characteristics of included studies

| **Study ID** | **groups** | **Previous history of major bleeding, N (%)** | **High bleeding risk, N (%)** | **Haemoglobin concentration, g/dL, mean (SD)** | **One Vessel CAD N (%)** | **Two Vessel CAD, N (%)** | **Three Vessel CAD, N (%)** | **Left Main Disease, N (%)** | **PCI for CTO, N (%)** | **Number of Treated Lesions, mean (SD)** | **Stent total length, mean (SD)** | **First generation DES (Sirolimus and/or Paclitaxel), N (%)** | **Second Generation DES (Everolimus and/ or zotarolimus and/or biolimus), N (%)** |
| --- | --- | --- | --- | --- | --- | --- | --- | --- | --- | --- | --- | --- | --- |
| Choi et al. 2025 (SMART-CHOICE 3) | Clopidogrel | 15 (0.5%) | 427 (15.5%) | 13.8 (1.7) | N.A | N.A | N.A | N.A | N.A | N.A | N.A | 0 | 100% for 2^nd^ and 3^rd^ generation |
|  | Aspirin | 21(0.8) | 448 (16.3%) | 13.8 (1.7) | N.A | N.A | N.A | N.A | N.A | N.A | N.A | 0 | 100% for 2^nd^ and 3^rd^ generation |
| Watanabe et al. 2024 (STOPDAPT-3) | Clopidogrel | N.A | 1582 (54.3%) | N.A | N.A | N.A | 128 (4.4%) | 173 (5.9%) | 135 (4.6%) | 1.33 (0.74) | 35.67 (20.77) | 0% | 100% |
|  | Aspirin | N.A | 1574(53.9%) | N.A | N.A | N.A | 135(4.6%) | 164(5.6%) | 148(5.1%) | 1.33 (0.74) | 35.67 (20.77) | 0% | 100% |
| Watanabe et al. 2024 (STOPDAPT-2) | Clopidogrel | N.A | 478(32.5%) | N.A | N.A | N.A | N.A | 44 (3%) | 59 (4%) | 1.25 (0.57) | 34.7 (24.3) | 0% | 100% |
|  | Aspirin | N.A | 536(36.1%) | N.A | N.A | N.A | N.A | 40 (2.7%) | 75 (5.1%) | 1.31 (0.64) | 35 (23.4) | 0% | 100% |
| Chung-Ang et al. 2024 | Clopidogrel | N.A | N.A | N.A | N.A | N.A | N.A | N.A | N.A | N.A | N.A | N.A | N.A |
|  | Aspirin | N.A | N.A | N.A |  |  |  | N.A | N.A | N.A | N.A | N.A | N.A |
| Lan et al. 2024 | Clopidogrel | N.A | N.A | N.A | 142 (33.6%) | 93 (22%) | 187 (44.3%) | N.A | N.A | N.A | N.A | N.A | N.A |
|  | Aspirin | N.A | N.A | N.A | 231 (39.7%) | 154 (26.5%) | 197 (33.8%) | N.A | N.A | N.A | N.A | N.A | N.A |
| Koo et al. 2021 (HOST-EXAM) | Clopidogrel | N.A | N.A | 13·7 (1·7) | 1367 (50·4%) | 855 (31·5%) | 488 (18%) | 142 (5·2%) | 257 (9·5%) | 1·3 (0·6) | 36·1 (24·2) | 54 (2%) | 2627 (96.9%) |
|  | Aspirin | N.A | N.A | 13·8 (1·6) | 1376 (50·4%) | 844 (30·9%) | 507 (18·6%) | 130 (4·8%) | 254 (9·3%) | 1·3 (0·6) | 35·7 (23·6) | 52 (1.9%) | 2651 (97.2%) |
| Sim et al. 2019 | Clopidogrel | N.A | N.A | 14.37(1.71) | 287 (53.9) | 160 (30.1) | 71 (13.4) | 14(2.6) | N.A | N.A | 29.1(13.5) | 13 (2.4) | 505 (94.80) |
|  | Aspirin | N.A | N.A | 14.47(1.86) | 690 (53.6) | 390 (30.3) | 175 (13.6) | 32(2.5) | N.A | N.A | 29(13.4) | 32 (2.5) | 1217 (94.6) |
| Park et al. 2016 | Clopidogrel | N.A | N.A | N.A | 301 (39.0) | 293 (38.0) | 177 (23.0) | 391 (50.7) | N.A | 1.33 (0.74) | 35.3 (20.8) | 245 (31.8) | 526 (68.2) |
|  | Aspirin | N.A | N.A | N.A | 1109 (44.9) | 831 (33.6) | 532 (21.5) | 1302 (52.7) | N.A | 1.33(0.74) | 30(19.3) | 1590 (64.3) | 882 (35.7) |
| Zhuang et al. 2014 | Clopidogrel (75 mg/d) | N.A | N.A | N.A | N.A | N.A | N.A | N.A | N.A | 3.2(1.1) | N.A | 223 (90.1) | 25 (9.9) |
|  | Clopidogrel (25 mg/d) | N.A | N.A | N.A | N.A | N.A | N.A | N.A | N.A | 3.1(0.9) | N.A | 200(89.2) | 24 (10.8) |
|  | Aspirin | N.A | N.A |  | N.A | N.A | N.A |  |  | 1.4 (1.2) |  | 254 (89.6) | 29 (10.4) |
| Jang et al. 2025 | Clopidogrel | N.A | N.A | 11.4 (1.7) | N.A | N.A | N.A | 612 (66.8) | 180 (19.6) | N.A | 48.2 (27.5) | 114 (12.4) | N.A |
|  | Aspirin | N.A | N.A | 11.3 (1.9) | N.A | N.A | N.A | 586 (69.9) | 150 (17.9) | N.A | 47.6 (26.25) | 101 (12.1) | N.A |

CAD: Coronary Artery Disease, CTO: Chronic Total Occlusion, DES: Drug-Eluting Stent, N: Number, N.A: Not Available, PCI: Percutaneous Coronary Intervention

## Supplementary Table 4: meta-regression of major adverse cardiovascular events (MACE)

| Variable | Coefficient (95% CI) | P-value | R² (%) |
| --- | --- | --- | --- |
| Mean age | **0.04 (0.01 to 0.07)** | **<0.01** | **100%** |
| Mean BMI | -0.27 (-0.56 to 0.02) | 0.06 | 0% |
| Mean LVEF | **-0.07 (-0.13 to -0.003)** | **0.04** | **100%** |
| Male sex, % | 0.01 (-0.02 to 0.04) | 0.63 | 0% |
| Hypertension, % | **0.02 (0.0004 to 0.03)** | **0.04** | **100%** |
| Dyslipidemia, % | 0.004 (-0.01 to 0.01) | 0.41 | 0% |
| History of stroke, % | 0.02 (-0.02 to 0.06) | 0.32 | 26.90% |
| Current Smokers, % | 0.004 (-0.01 to 0.02) | 0.61 | 100% |
| previous PCI, % | 0.01 (-0.05 to 0.06) | 0.83 | 0% |

BMI: body mass index, LVEF: left ventricular ejection fraction, PCI: Percutaneous Coronary Intervention.

R² (%) = proportion of heterogeneity explained by the covariate

## Supplementary Table 5: Inclusion and Exclusion criteria for each study

| **Study ID** | **Inclusion criteria** | **Exclusion criteria** |
| --- | --- | --- |
| Choi et al.2025 (SMART-CHOICE 3) | Eligible patients were aged ≥19 years, had successful PCI with a drug-eluting stent and standard DAPT duration, no post-PCI cardiovascular events, and at least one high-risk clinical (e.g., prior MI or diabetes) or complex lesion characteristic (e.g., bifurcation, CTO, long lesions, multivessel PCI, multiple stents, in-stent restenosis, severe calcification, or ostial lesions). | long-term treatment with oral anticoagulants; use of DAPT for any reason other than coronary artery disease; use of single antiplatelet therapy at screening; or contraindications to aspirin or clopidogrel. |
| Watanabe et al. 2024 (STOPDAPT-3) | Patients with ACS or high bleeding risk (HBR) undergoing planned PCI with Xience cobalt–chromium everolimus-eluting stents and eligible for 1 month of DAPT with aspirin and a P2Y12 inhibitor were included. | patients who had the clinical event of interest within 30 days, and those who did not receive PCI for the absence of suitable coronary lesions. |
| Watanabe et al. 2024 (STOPDAPT-2) | patients hospitalized for PCI with cobalt-chromium everolimus- eluting stents (Xience series, Abbott Vascular) and able to DAPT with aspirin and a P2Y12 inhibitor for at least 1 month. | N.A |
| Chung-Ang et al. 2024 | N.A | N.A |
| Lan et al. 2024 | Patients who underwent PCI with DES and remained event-free after completing 12 months of DAPT were assigned to aspirin or clopidogrel groups within a ±3-month window around the 12-month follow-up. | prior ischemic or bleeding events, contraindications to aspirin or clopidogrel, use of oral anticoagulants, pregnancy, severe comorbidities (e.g., organ failure, malignancy, psychiatric illness), recent pulmonary embolism or aortic dissection, use of other antiplatelets, and refusal to follow up. |
| Koo et al. 2021, Kang et al. 2024(HOST-EXAM) | Patients aged ≥20 years who had maintained dual or triple antiplatelet therapy for 12 ± 6 months after PCI with DES, had no subsequent clinical events, planned to switch to monotherapy, and provided written informed consent. | hypersensitivity or contraindications to clopidogrel, inability to discontinue current antiplatelet therapy, use of other antiplatelets during follow-up, history of major bleeding (BARC ≥3) leading to antiplatelet discontinuation within 3 months, and bleeding diathesis. |
| Sim et al. 2019 | AMI patients treated with DES, DAPT during hospitalization, Plan to change to antiplatelet monotherapy | Patients who received anticoagulants or other antiplatelet agents such as cilostazol were excluded. Patients who suffered MACE, repeat revascularization, or major bleeding were excluded. |
| Park et al. 2016 | Patients underwent PCI with DES. Patients who were event-free at their 12-month follow-up | Patients received DAPT, warfarin, or antiplatelet therapy other than aspirin or clopidogrel subsequent to the 12-month follow-up after the index procedure. |
| Zhuang et al. 2014 | patients who had undergone PCI, completed 12-month DAPT | The patients who had hypoxic encephalopathy, malignancies, or chronic hemodialysis (HD), were taking warfarin or single antiplatelet therapy, or had been transferred to other hospitals after a successful PCI at the first affiliated hospital of Sun Yat-sen University. |
| Jang et al. 2025 | Consecutive patients at every centre successfully treated with one or more DES approved by the US FDA or CE mark and who were adequately loaded with clopidogrel were eligible for enrolment, regardless of patient or lesion complexity | The occurrence of a major complication during the procedure or before platelet function testing, or if bypass surgery was planned after PCI. Any P2Y12 inhibitor other than clopidogrel PCI strategy other than DES Needed oral anticoagulant (e.g. atrial fibrillation) |

ACS: acute coronary syndrome, DAPT: Dual Antiplatelet Therapy, DES: Drug-eluting stent, HBR: High Bleeding Risk, MACE: Major Adverse Cardiovascular Events, MI: myocardial infarction, N.A.: Not Available, NACE: Net Adverse Clinical Events, PCI: Percutaneous Coronary Intervention, TIMI: Thrombolysis in Myocardial Infarction, TLR: Target Lesion Revascularization, TVR: Target Vessel Revascularization.
